# Mass extinction triggered the early radiations of jawed vertebrates and their jawless relatives (gnathostomes)

**DOI:** 10.1126/sciadv.aeb2297

**Published:** 2026-01-09

**Authors:** Wahei Hagiwara, Lauren Sallan

**Affiliations:** Macroevolution Unit, Okinawa Institute of Science and Technology, Onnason, Kunigamigun, Okinawa 904 0495, Japan.

## Abstract

Most vertebrate lineages are first recorded from the mid-Paleozoic, well after their Cambrian origin and Ordovician invertebrate biodiversification events. This delay has been poorly understood and is usually attributed to sampling and long ghost lineages. We analyzed newly compiled databases of Paleozoic vertebrate occurrences, biogeography, and ecosystems, revealing that the Late Ordovician Mass Extinction (~445 to 443 million years ago) triggered parallel, endemic radiations of jawed and related jawless vertebrates (gnathostomes) in isolated refugia. Postextinction ecosystems hosted the first definitive appearances of most major vertebrate lineages of the Paleozoic “Age of Fishes” (and today), following the loss of ubiquitous stem-cyclostome conodonts, nascent faunas of other gnathostomes, and pelagic invertebrates. Turnover and recovery patterns matched those following climatically similar events like the end-Devonian mass extinction, including a postextinction “gap” with low biodiversity. The prolonged Silurian recovery, and the challenges of oceanic dispersal, likely further delayed the dominance of jawed gnathostomes for millions of years after the first fossil jaws.

## Teaser

A mass extinction 445 million years ago triggered the rapid rise of armored and jawed vertebrates.

## INTRODUCTION

The more than 70,000 living vertebrate species belong to just two clades: the many jawed gnathostomes (tetrapods and “fishes”) and the few jawless cyclostomes (lampreys and hagfishes) (*1*, *2*). Stem vertebrates first appeared more than 520 million years ago (Ma) in famous “Cambrian Explosion” faunas such as Chengjiang and Burgess Shale alongside the early members of other phyla (*2*, *3*). Cyclostomes diversified shortly thereafter in the form of their ubiquitous marine stem members, the “true” conodonts (euconodonts) (*2*–*4*), in line with Cambrian and Ordovician invertebrate biodiversification events (*5*, *6*). Gnathostomes, including the earliest jawed species and jawless stem lineages (i.e., “armored agnathans” or “ostraderms”), are widely assumed to have likewise diversified in the early Paleozoic (*7*, *8*), based on mid-late Cambrian (~521 to 487 Ma) (*3*, *9*) estimated divergence dates for crown vertebrates (*10*) and scattered stem-gnathostome–like and crown-gnathostome–like fossils from the mid-late Ordovician (~471 to 443 Ma) (*2*, *7*, *10*, *11*). However, gnathostomes did not show up in any real abundance, or at all in most regions, until the Silurian (~443 to 420 Ma) (*2*, *8*–*10*, *12*). A 50- to 100-million-year gap is therefore inferred within the record of most gnathostome lineages, particularly among jawed forms. The gap is usually attributed to poor sampling or environmental constraints (*3*, *7*, *8*, *12*), ignoring the global abundance of preservationally similar conodonts and ecologically analogous mobile, pelagic invertebrates (*2*, *10*).

The early Paleozoic “gnathostome gap” was punctuated by global events that were critical milestones in the evolution of marine biodiversity but have received only passing consideration by vertebrate paleontologists (*2*, *8*, *10*, *12*). This includes the double-pulsed Late Ordovician Mass Extinction (LOME; ~445 to 443 Ma) (*9*), a “Big Five” event marked by prolonged global fluctuations in temperature, alterations in ocean chemistry including essential trace elements, sudden polar glaciation, and sea level changes, which drowned or marooned coastal faunas (*13*–*16*). The LOME has been linked to substantial losses and lineage turnover in pelagic and predatory invertebrates (e.g., ammonoids and arthropods), as well as conodonts (*14*). In all these characteristics, the LOME was highly similar to another glaciation-linked event within a longer crisis interval, the end-Devonian mass extinction or Hangenberg Event (EDME; 359 Ma) (*9*), which profoundly devastated vertebrate ecosystems (*2*, *16*–*18*). Following the LOME, the Silurian era (~443 to 420 Ma) (*9*) comprised a 23-million-year recovery interval, marked by reorganization and diversification of mobile invertebrate faunas (*15*), and coincident with the documented first appearance of most major gnathostome clades (*3*, *10*). The EDME was also followed by a similarly prolonged Mississippian (359 to 323 Ma) (*9*) recovery interval notable for the increased diversification and abundance of living gnathostome lineages (*2*, *17*, *18*). The parallels between the Late Ordovician and end-Devonian in terms of drivers and influence on mobile marine animals suggest a possible role for mass extinction in observed Ordovician-Silurian changes in gnathostome diversity.

A major stumbling block in reconstructing early vertebrate diversification has been insufficient and poorly vetted global occurrence databases (*2*, *10*). To determine the relationship between the early Paleozoic gnathostome gap and the LOME, we compiled the most complete record of early to mid-Paleozoic gnathostome occurrences to date. Our database contained 1157 occurrences for 449 gnathostome species stretching from the early Ordovician to the end-Silurian (~487 to 420 Ma) (*9*) and 2546 species records for the Devonian (~420 to 359 Ma) (*9*). As a comparison, we downloaded stem-cyclostome euconodont occurrences from the Paleobiology Database (*19*). We used these records to produce genus-level, stage-binned diversity curves for all vertebrates, 13 major gnathostome groups, and five major geographic regions over the Ordovician-Silurian, as well as species-level curves for vertebrates and gnathostome groups (fig. S3). We also assembled curves for richness within each stage per million years to correct for variations in stage length (see Results). To account for sampling bias and observe regional trends, we sorted gnathostome species occurrences into specific Ordovician-Silurian fossil assemblages based on shared locality and age. We used these assemblage-level data to designate 169 distinct gnathostome “faunas” containing species which coexisted during a set interval in a specific geographic location and shared habitat (*17*). Comparison of our faunas allowed us to detect changes in local species richness within major clades and overall gnathostome faunal composition across time and environments (see Results) (*17*). Next, we tracked gnathostome biodiversity across five major regions to determine trends in biogeography. Last, we recategorized species as jawed or jawless based on the phylogenetic position and diagnosis for their assigned groups to track relative changes in faunal composition over the Ordovician-Silurian and global genus richness over the Ordovician-Devonian (see Results).

## RESULTS

**Fig. 1.**
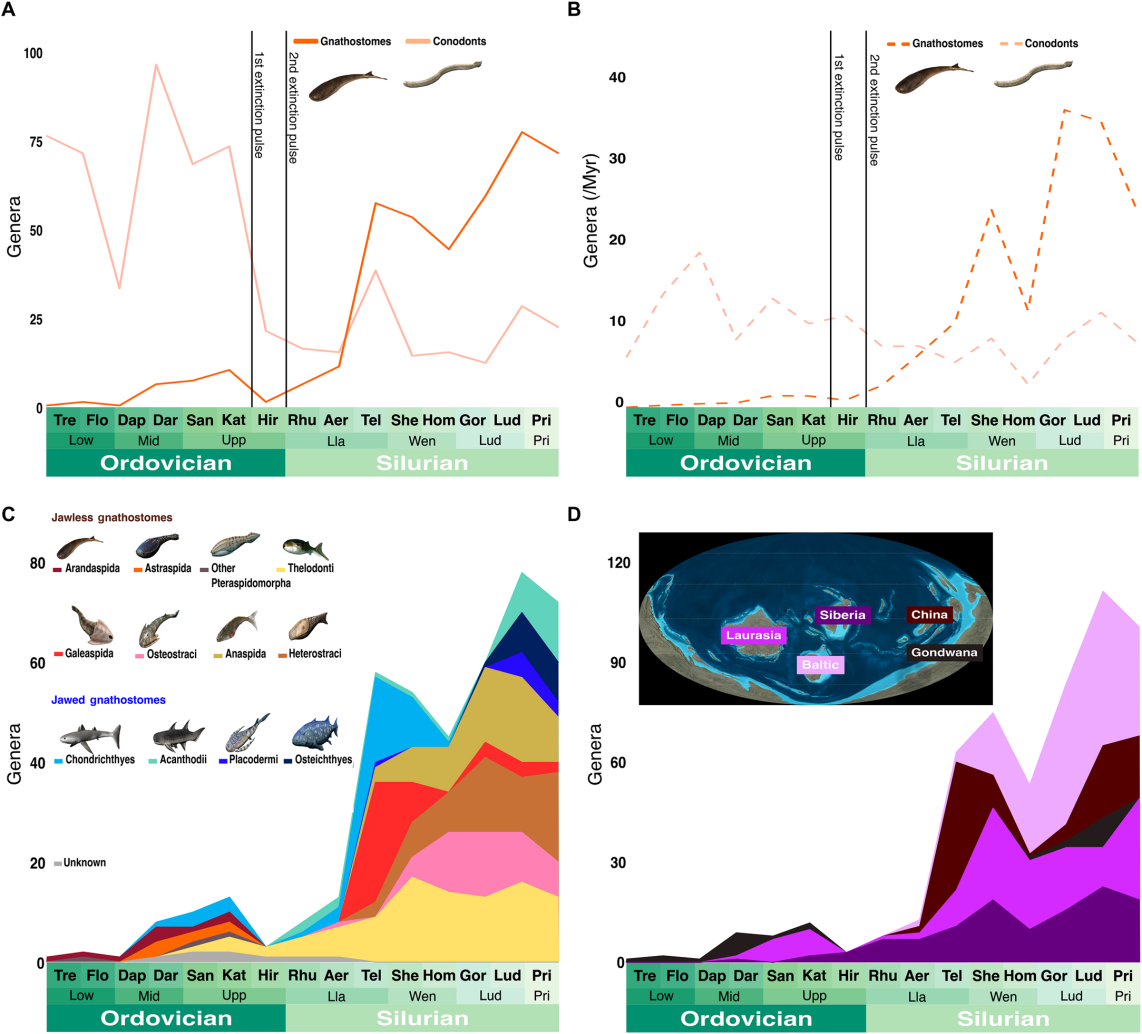
Genus-level diversity curves for gnathostomes from the Ordovician to Silurian. (**A**) Genus-level, stage-binned curves of global taxonomic richness for gnathostomes (*N* = 418) and conodonts (*N* = 613). (**B**) Diversity curves based on richness per million years (Myr) in each stage. (**C**) Diversity curves for genus-level richness per stage in 13 Paleozoic gnathostome classes (fig. S1). (**D**) Diversity curves for stage-binned gnathostome genus-level richness in five major regions (fig. S1). Species-level curves are shown in fig. S3. Paleomap used with permission 2016 Colorado Plateau Geosystems. Vertebrate reconstructions by N. Tamura [used with permission, originally published under a CC BY-SA license (https://creativecommons.org/licenses/by-sa/4.0/deed.en)].

**Fig. 2.**
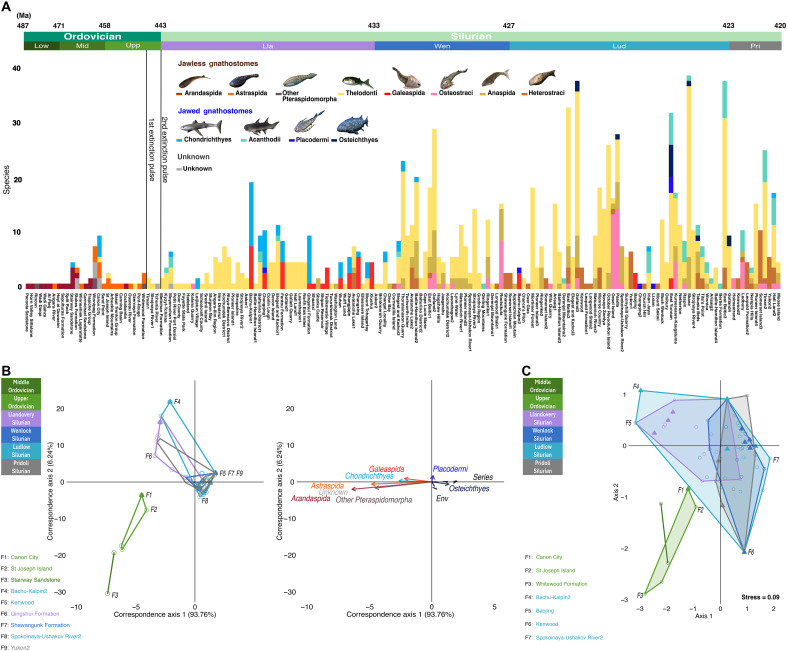
Change in the composition of gnathostome faunas through time. (**A**) Histogram of species-level faunal composition for all 169 Ordovician-Silurian faunas (1156 species-level occurrences) (data S1). Species are binned into 13 major gnathostomes groups as in Fig. 1. The geological timescale (*9*) corresponds to the series-level age of each site as some faunas span multiple stages. (**B**) CCA of group-binned species diversity for a subsample of 101 faunas containing at least three species (1076 species occurrences). Filled triangles indicate marine sites, whereas open circles indicate nonmarine. Left: Fauna ordination plot (*N* = 101), with the most disparate Ordovician and Silurian faunas on each axis indicated (*N* = 9). Sites F5, F7, and F9 shared the same score and thus overlap at the same coordinate. Right: Group ordination plot (*N* = 13). Biplot arrows represent the eight gnathostome groups with the highest contributions to fauna position and two explanatory variables (series or geological time; environment) based on their correlation with taxon distribution. (**C**) NMDS based on gnathostome group species richness at faunas and Bray-Curtis distances, with the seven most disparate Ordovician and Silurian faunas on each axis indicated. Alternative groupings combining Chondrichthyes and Acanthodii shows the same result (figs. S11 to S17)*.* Vertebrate reconstructions by N. Tamura [used with permission, originally published under a CC BY-SA license (https://creativecommons.org/licenses/by-sa/4.0/deed.en)].

First, we found that total-group gnathostome and cyclostome (euconodont) genera exhibited a classic “double wedge” pattern with turnover centered on the two end-Ordovician extinction pulses (Fig. 1A). Conodont stage-binned genus-level curves suggested high richness in the Ordovician and extreme losses over the end-Katian (~445 Ma) (Fig. 1A) (*9*). Time correction blunted some of the LOME impact, suggesting stability and then a muted decline in genus-level diversity over the end-Hirnantian (~443 Ma) (*9*) that continued in the Silurian (Fig. 1B and fig. S3B). This is in line with a prior report of conodont lineage turnover at consistent richness levels during the LOME interval (*14*). Conodont genus richness never recovered to Ordovician peaks but settled below a maximum of 39 genera per stage in the Silurian (Fig. 1A) and ~20 genera per stage thereafter, suggesting a limit that held until their extinction in the Triassic (*6*). In contrast to conodonts, gnathostome genus-level and species-level diversity was relatively low throughout the Ordovician and even lower in time-corrected curves, with a slight increase in the Dapingian to Katian (~469 to 445 Ma) (Fig. 1, A and B, and fig. S3, A, B, and E) (*9*). Gnathostomes suffered apparent losses over the Katian, although this may be also have been a temporal artifact (Fig. 1, A and B). We found that the Silurian featured several rounds of postextinction gnathostome diversification (or recovery) as part of a general increase in global diversity that stretched to the Devonian and followed 3 to 5 million years of very low global richness (Fig. 1 and fig. S3). Gnathostome global richness reached ever higher peaks (Fig. 1, B and C, and fig. S3), and faunas demonstrated greater richness and global homogeneity toward the end of the recovery interval (Fig. 2).

**Fig. 3.**
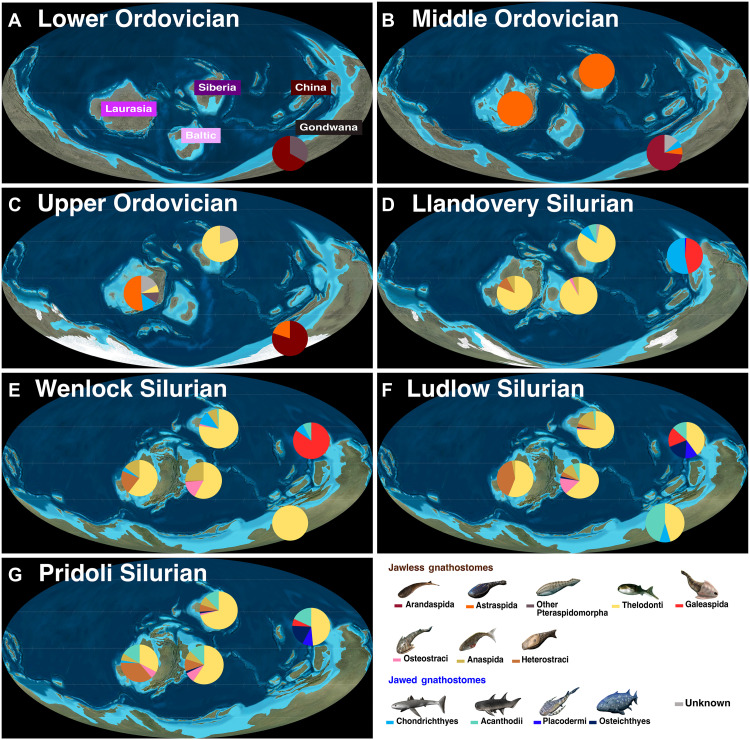
Species-level richness by geological series for 13 gnathostome groups in five Paleozoic geographic regions (Gondwana, Siberian, Laurasia, Baltic, and China). (**A**) Lower Ordovician (~487 to 471 Ma). (**B**) Middle Ordovician (~471 to 458 Ma). (**C**) Upper Ordovician (~458 to 443 Ma). (**D**) Llandovery Silurian (~443 to 433 Ma). (**E**) Wenlock Silurian (~433 to 427 Ma). (**F**) Ludlow Silurian (~427 to 423 Ma). (**G**) Pridoli Silurian (~423 to 420 Ma). Maps used with permission 2016 Colorado Plateau Geosystems Inc. Vertebrate reconstructions by N. Tamura [used with permission, originally published under a CC BY-SA license (https://creativecommons.org/licenses/by-sa/4.0/deed.en)].

To determine whether the Silurian global diversification of gnathostomes resulted from LOME-related causes, we examined our records for Ordovician-Silurian groups, faunas and regions in more detail. On the basis of the composition of our faunas, the apparent increase in Mid-Late/Upper Ordovician gnathostome diversity (Fig. 2A) was mostly driven by three well-sampled intertidal localities in Australia and North America (Stairway Sandstone, Canon City, and Winnipeg) (Figs. 2A and 3C), two of which produce scales that have been attributed to among the earliest-known jawed chondrichthyans (Fig. 2A) (*2*, *3*, *7*, *10*, *20*). There was also an increase in the number of low richness faunas containing members of Ordovician-specific jawless clades with clear habitat restrictions (*10*). Arandaspids (e.g., *Sacabambaspis*) only occurred in intertidal and subtidal areas along Gondwanan coastlines (Fig. 3) (*10*). In contrast, astraspid pieces have been the dominant gnathostome fossils recovered at Mid-Ordovician sites at then-equatorial shallow marine areas of Laurasia and Siberia, joined by thelodont scales in faunas from near the end of the period (Fig. 3) (*21*). The biogeographic division between Northern and Southern continents was reflective of similar separation in invertebrate faunas, linked to intervening deep seas and unfavorable, East-West currents (*22*). Within these areas, the few well-sampled Ordovician gnathostome faunas were highly homogeneous and distinct compositionally from postextinction ecosystems in our ordinations (Fig. 2, B and C). Only the Canon City fauna was positioned close to Silurian localities in our nonmetric multidimensional scaling (NMDS) plot, based on the presence of multiple Chondrichthyes (“shark”) scale-based species alongside Ordovician-only forms like astraspids (Fig. 2C). In contrast, although the earlier Stairway Sandstone from Australia contains a similar chondrichthyan scale taxon (*Tantalepis*) (*20*), this fauna is the most differentiated from Silurian faunas in our canonical correspondence analysis (CCA), due to the high number of resident arandaspids (Fig. 2B).

Any nascent Ordovician gnathostome diversification appears to have been disrupted by the first glaciation pulse at the end of the Katian (~445 Ma) (*9*) (Figs. 1C and 2A). Prior work has found that intrapulse Hirnantian mobile invertebrate and conodont faunas were a diverse mix of Ordovician relicts and diversifying species (*14*). We recovered only three Hirnantian gnathostome faunas from Siberia, consisting of isolated thelodont scales belonging to just one or two form taxa as well as ichthyoliths unattributable to any prior species (Figs. 2A and 3C). The initial glaciation pulse marked the latest appearance of all more robust and armored Ordovician gnathostomes, including astraspids, arandaspids, and other poorly known genera, which have been attributed to Pteraspidimorpha outside the radiation of heterostracans and thus may be relatives of the former two lineages (Fig. 3, A to C). Nearly all of Gondwana exhibited a ~10 to 15 million year interval completely devoid of gnathostome fossils, a span stretching from the entire Hirnantian to the first half of the Silurian, after which richness remained low (Figs. 2A and 3D) (*23*). This is observed even in areas that feature an otherwise robust Paleozoic record and have been subject to intensive sampling, such as Australia (Figs. 1D and 3) (*23*). The gnathostome-depleted region was similar to the maximum extent of glaciation in the LOME (*14*). Although some prior authors have proposed an “Out of Gondwana” circumpolar hypothesis for Silurian gnathostome diversification (*21*), this is now ruled out by apparent extinction-related extirpation.

Following the LOME, gnathostomes exhibited fundamentally changed faunal and biogeographic diversity patterns, which are suggestive of deep losses. The earliest Silurian age, the ~3-million-year Rhuddinian era (~443 to 440.5 Ma) (*9*) of the Llandovery epoch, was coincident with “Talimaa’s Gap,” a term used for a previously inferred but neglected interval of low or missing vertebrate diversity (particularly in Gondwana as above) (*10*, *12*, *23*, *24*). We confirmed this gap quantitatively in our dataset, where Rhuddinian diversity increased only slightly from the Hirnantian (Fig. 1). The few Rhudinnian and early Aeronian (~440.5 to 438.5 Ma) (*9*) age gnathostome-bearing sites produced almost exclusively ichthyolith form taxa (Fig. 2A). Tropical Laurasian and Baltic regions were dominated by thelodonts in line with the nascent faunas of the Hirnantian (Figs. 2A and 3D). In Siberian localities, thelodonts were joined by scales assigned to “chondrichthyan” or “acanthodian” form taxa such as tsunacanthids and elegestolepids, which differed from Ordovician types and disappeared after the Llandovery (Figs. 2A and 3D) (*25*).

All other major gnathostome groups made their first Silurian appearances in the Aeronian or Telychian (~438.5 to 433 Ma) (*9*), 3 to 10 million years after the LOME. This occurred in line with increases in global genus richness (Fig. 1, A and B). Most jawless stem-gnathostome clades, including osteostracans, galeaspids, and heterostracans (excluding the distinct, exclusively Ordovician Pteraspidomorpha taxa noted above), entered the Silurian record in separate regions (Fig. 3D). These groups exhibited initially low levels of richness and abundance ahead of more consistent diversification in the later Silurian (Fig. 1D). For example, although the earliest known osteostracan headshield came from an Aeronian quarry in Baltic Estonia, there was a subsequent gap until the Wenlock (~433 to 427 Ma) (*9*) of the same region (Figs. 2A and 3, D and E). The Silurian emergence of heterostracans was even further delayed as these initially appeared in the late Telychian record of Laurasia in the present Canadian Arctic. This created a 10-million-year gap (or ghost lineage) from last preextinction occurrence of astraspids and other pteraspidomorph genera in the Katian of North America (Fig. 2A). Galeaspids made their first appearance in the Aeronian of China and remained in this isolated region until their extinction in the Devonian (*2*, *10*) (Figs. 1C, 2, and 3, D to F).

Chinese galeaspid-bearing faunas of the Aeronian and early Telychian contained the earliest definitive evidence for jaws and thus permit the earliest identification of jawed gnathostome lineages from body fossil material (*8*, *26*). In our ordinations, such “chondrichthyan-galeaspid” faunas were distinct from faunas of the Ordovician and those from the early Silurian of Laurasia and the Baltic, as well as a thick cluster of late Silurian faunas on correspondence axis 1 in our CCA and axis 2 in our NMDS plot (Fig. 2, B and C). A distinct China/East Asia biome persisted throughout most of the Silurian (Fig. 3, D to F). Our results suggest that the initial diversification of jawed gnathostomes, and the origins of most major lineages, apparently occurred within a distinct refugium (*8*) in the first 10 million years postextinction, even if stem-members first appeared elsewhere before the LOME (*3*, *7*, *10*, *11*). In the early Silurian, China was an equatorial offshoot of otherwise vertebrate poor Gondwana, separated from other continents by a deep sea (Fig. 3). Although Ordovician sediments in China have not yet produced gnathostome material, it is possible that this region hosted taxa found in nearby Australian coastal systems, such as arandaspids and the producers of jawed gnathostome-like scale forms and ichthyoliths (*21*, *23*). Survivors could have given rise to the local endemics in the postextinction interval, after the extirpation of relatives from Australia itself (Figs. 1C and 3D).

**Fig. 4.**
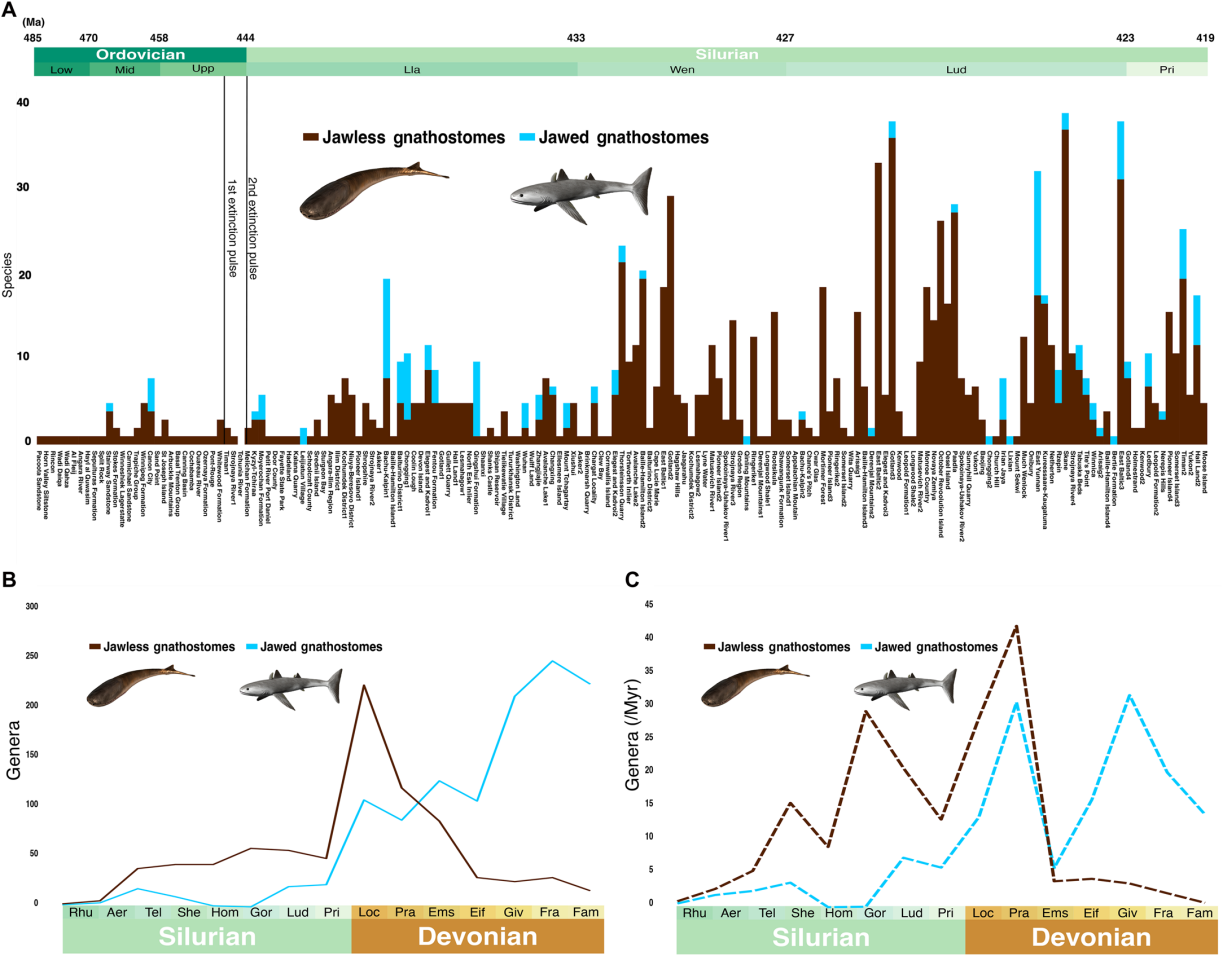
Comparison of jawed and jawless gnathostome diversity trends over the mid-Paleozoic. (**A**) Histogram showing species-level richness for jawed and jawless gnathostomes for faunas from Ordovician to Silurian, using the same dataset as in Fig. 1A excluding “Unknown” species (*N* = 1144 species occurrences). Group assignment based on taxonomic attribution, rather than direct evidence for jaws. (**B**) Global diversity curves for stage-binned genus richness for jawless (*N* = 830) and jawed (*N* = 1188) gnathostome groups for the Silurian and Devonian, with the timescale based on (*9*)*.* (**C**) Global diversity curves for stage-binned genus richness per million years. Vertebrate reconstructions by N. Tamura [used with permission, originally published under a CC BY-SA license (https://creativecommons.org/licenses/by-sa/4.0/deed.en)].

Endemic diversification of jawed gnathostomes within Chinese ecosystems continued throughout the entire recovery interval (Figs. 1, C and D, 2A, and 3, D to G) (*8*). Ongoing geographic isolation in nearshore waters (*10*) likely contributed to an observed positive relationship between South China faunal size and the global richness of jawed gnathostomes for most of the Silurian (Figs. 1, C and D, and 4, B and C). However, divergence within South China faunas was not limited; heterogeneity and regional diversity increased as the recovery proceeded. The initial intervals following mass extinctions typically feature homogeneous “disaster” or “flux” ecosystems dominated by sets of short-lived lineages, which are then superseded by the diversification of groups that define the rest of the recovery interval and beyond (*27*). It is possible that the homogeneous, stem-group chondrichthyan and galeaspid-dominated faunas of the Llandovery of South China fell into this category. These were subsequently replaced by a more diverse fauna of placoderms, osteichthyans, and polyphyletic acanthodians in the later Silurian (Figs. 1C and 3, D to G). A Homerian era gap between these South China faunas may represent either local sampling issues or a regional event, of which there were many in the unstable Silurian (Figs. 1, C and D, and 4B) (*15*).

The spread of most gnathostomes from separate refugia occurred slowly during the Silurian recovery interval. Initially, dispersal over long distances was limited to specific lineages based on morphology (Figs. 2A and 3, D to G) (*10*). During the early-mid Silurian, faunas outside China were dominated by pelagic thelodonts (Figs. 2A and 3D). Gnathostome communities were reestablished in Gondwana by the Wenlock era (Fig. 3E), but samples have consisted entirely of thelodont scales (Fig. 3E). Thelodonts also disrupted the distinctiveness of South China and other East Asia faunas through invasion by the Ludlow (~427 to 423 Ma) (*9*) (Fig. 3F), coincident with the disappearance of older scale-based and spine-based jawed taxa (e.g., mongolepids) and a reduction in galeaspid richness mentioned above (Figs. 1C and 2A). Likewise, streamlined anaspids such as *Birkenia* spread to Wenlock era Siberian and Baltic faunas within a short time after their initial appearances in the late Telychian of Laurasia (Figs. 2A and 3, D and E). In contrast, more environmentally restricted, armored (macromeric) jawless gnathostome lineages (e.g., galeaspids, osteostracans, and heterostracans) (*10*) remained limited to their natal regions for most of the Silurian, reaching adjacent lands only in the latest Silurian or Devonian in line with the formation of Pangaea (*15*), if at all (Fig. 3, D to G).

Most jawed gnathostomes showed limited dispersal throughout the Silurian recovery interval, remaining geographically restricted through the Pridoli (423 to 420 Ma) (*9*) (Figs. 1C and 3, D to G) like the coincident galeaspids and most armored jawless gnathostomes elsewhere. Dispersal among even micromeric (small-scaled, flexible) jawed forms seems to have been delayed substantially relative to jawless gnathostomes such as thelodonts. Jawed gnathostomes were apparently restricted to China and possibly Siberia throughout most of the Llandovery, with species in the latter consisting of a few isolated scales among a plethora of similar thelodont remains (Figs. 2A and 3E). By the Ludlow, Siberian ichthyoliths had been replaced by body fossils of acanthodians and the probable stem-osteicthyan *Andreolepis*, which may have spread from China via prevailing currents (Fig. 3F). Acanthodians and other chondrichthyans moved rapidly to the Baltic following the counterclockwise conveyor belt of currents (*15*, *22*). They also apparently moved against the flow via shallow seas to least one Gondwanan Ludlowera locality (New Guinea; Figs. 2A and 3F). Osteichthyans remained rare outside China throughout the later Silurian, with just one or two species present in scattered Baltic faunas (Figs. 2A and 3G). In contrast, acanthodian diversity increased markedly in Pridoli faunas across all regions including Laurentia, perhaps enabled by the replacement of deep straits with connected shallows as Pangaea formed (Fig. 2G).

## DISCUSSION

The Silurian reorganization and expansion of gnathostome biodiversity appears to have been driven by disruption to existing stable communities during the LOME, including extirpation of incumbents and resultant ecological release, in line with patterns across other mass extinctions (*17*, *27*, *28*). Conodonts and other pelagic victims of the LOME such as ammonoids and arthropods were the most likely candidates for such incumbents (Fig. 1, A and B) (*14*). However, the known ecomorphological diversity of Silurian gnathostomes far outstrips that inferred for preextinction conodonts, which has been based on only two known Paleozoic lamprey-like euconodont body fossils: one recovered just after the initial extinction and turnover pulse for the group (*5*) and the other from the Carboniferous (323 to 299 Ma) (*29*, *30*). This could suggest that reorganization of vertebrate faunas after the LOME went beyond simple replacement (*10*). However, it is also possible that Ordovician vertebrates exhibited higher ecomorphological diversity than realized, particularly given the almost total lack of body fossils and poor preservation potential for such soft-bodied lineages lacking scales or dermal bone (*3*, *7*, *31*). The hagfish-like Hirnantian euconodont *Promissum* was also poorly preserved (*29*), but it could represent a common postextinction body plan, which was carried forward by later ecologically conservative crown-cyclostomes (*4*). In any case, Ordovician conodonts are known to have exhibited high levels of diversity in their feeding traits and lived in a range of environments (*10*, *32*), so it is probable they filled a large set of ecological roles in Ordovician seas.

Ordovician gnathostomes might also have contained a greater diversity of lineages and ecomorphologies that ultimately succumbed to the LOME, rather than being simply early representatives of known Silurian taxa and their associated forms as previously assumed (*3*). North American and Australian Ordovician fossil sites have produced “chondrichthyan” and “gnathostome” scales of indeterminant lineage and rare histology for the Paleozoic (Fig. 2, A and C) (*3*, *7*, *11*, *20*, *21*). These forms have been noticeably divergent from scales found in early Silurian faunas in China and Siberia (*7*, *25*), as well as those found on Silurian body fossils (*23*), despite being made of the same dermal hard tissues. We listed a few Ordovician ichthyolith taxa as “Unknown” because they have combinations of traits that have led to their attribution to different later gnathostome lineages at different times (data S1) and thus may represent stem-group experimentation (*3*). More completely known taxa from Ordovician “armored” jawless gnathostomes, such as arandaspids and astraspids, exhibited a few character states shared by later lineages such as heterostracans, leading to their inclusion in clade-specific phylogenies of “Pteraspidomorpha” (*3*, *10*, *33*, *34*). Yet, these too exhibited many divergent traits and forms, and lack derived characters which define Silurian lineages (*33*, *34*). There is space for an undersampled, unknown diversity of gnathostomes within the widespread occurrence of broken pieces of fossils attributed to pteraspidomorphs in coastal faunas, but these remains have been hard to place within that clade with certainty because of divergence from later taxa (Fig. 2A) (*3*, *10*). Even the few Ordovician thelodont scale-based taxa never occurred again after the second glacial pulse, replaced by more regular Silurian forms such as *Loganellia* (data S1). Therefore, it is likely that there was a fully distinct Ordovician gnathostome fauna, perhaps involving very early members of Silurian lineages and localized diversification in shallow waters (*10*). In any case, all known Ordovician taxa appear to have been eliminated or substantially changed over the LOME, suggesting complete extinction-related turnover within gnathostomes (Fig. 1, A to C).

We found strong evidence for a prolonged postextinction recovery (*15*, *28*), in which most Silurian gnathostome lineages diversified gradually and intermittently during an initial period of otherwise very low global richness. The Llandovery epoch (~443 to 433 Ma) (*9*) featured delayed vertebrate appearances and reappearances in distinct, regionally homogeneous and low diversity postextinction faunas. This pattern is similar to vertebrate diversity during the post-EDME Tournasian (359 to 347 Ma) (Fig. 2) (*2*, *9*, *17*). Given proximity to the end-Hirnantian glaciation event, Talimaa’s Gap is analogous to the “Romer’s Gap” diversity trough following the EDME glaciation and similar intervals after other events (*2*, *17*, *27*). With the exception of Siberia, many early Llandovery faunas occurred in regions that have lacked good Ordovician gnathostome records such as China and the Baltic (Fig. 1, A and B). Likewise, early Silurian sediments near productive Ordovician sites in Gondwana and elsewhere have not yet produced gnathostome samples (Fig. 3, A to D). This suggests extreme biogeographic shifts throughout the Hirnantian and early recovery interval. In line with observations after other mass extinctions (*17*, *28*), there was very low initial diversity and richness in most places during the first 3– to 5 million years of the recovery (Talimaa’s Gap, as above) (*10*) and even the rest of the 10-million-year Llandovery in some areas. Any surviving populations likely fell below the critical mass needed for preservation (*28*). Such low local diversity is supported by the fact that most of our early Llandovery faunas consist of less than five species from only one or two gnathostomes groups.

We observed a high level of endemism in gnathostomes from the very beginning of the Silurian, a stark change from the wide geographic ranges observed for most Ordovician vertebrates. Regional distinctions in faunal composition (Figs. 1D and 3) suggested that ecological reorganization and diversification initially occurred within specific, long-lasting extinction refugia (*35*). This allowed the parallel emergence of novel species in different faunas and perhaps some degree of early experimentation in form. For example, many Rhudinnian faunas contained distinct taxa which do not appear either earlier or later on, such as mongolepid chondrichthyans and specific thelodont genera (Fig. 2A) (*7*, *19*). These may be interpreted as short-lived “disaster taxa” or residents of transient disaster faunas or flux ecosystems (like those noted above in Llandovery South China), both common postextinction phenomena in invertebrate records (*27*, *28*, *36*). We also found that each early Silurian region featured a distinct set of novel benthic forms, such as galeaspids and osteostracans. Such lineages exhibited divergence within highly-restricted nearshore environments (*10*), alongside novel pelagic gnathostomes such as acanthodians and anaspids. Thus, whereas gnathostome diversity remained low for millions of years after the LOME, separate refugia enabled high levels of trait divergence and increasing ecomorphological diversification in endemic areas protected from external competition. The postextinction ecosystems created the template for widespread gnathostome communities in the Devonian “Age of Fishes” and in modern oceans.

## MATERIALS AND METHODS

### Dataset assembly procedure

We compiled a dataset of gnathostome occurrences from the Ordovician-Silurian interval using the descriptive, stratigraphic, and paleoecological literature. The details of sources are provided in data S1, S3, and S4, where we specified which references were used to determine taxonomic classification, age, locality, and environment or habitat. We primarily based taxonomic information on the latest data available, as well as general, updated resources including Sepkoski’s compendium (*6*), *Family-Group Names of Fossil Fishes* (*37*), and the Fossiilid.info database (*38*). As we have found that age assignments in the Paleozoic vertebrate fossil literature are often inexact, based on outdated stratigraphy, and/or list only regional stages (*17*), we have confirmed or changed age ranges using the most recent stratigraphic literature for the fossil-bearing geological unit (formation or member) and/or index fossil or isotopic data when available (data S1, S3, and S4). The final gnathostome dataset included 1576 occurrences, 449 species-level records, and 219 genus-level records from the Ordovician-Silurian interval. For genus-level analyses, we excluded indeterminate records (gen. indet.), whereas they were retained for analyses of faunal composition (see below). We assigned fossil occurrences to 169 distinct gnathostome assemblages (faunas) on the basis of shared localities, geological settings, environment, and age, following the procedure of Sallan and Coates (*17*) (data S1). Similarly, we compiled taxonomic and age information for Devonian occurrences using the same approach (data S5). However, Devonian faunal assignments and Devonian species-level data were not compiled for this study as this interval is too far removed from the LOME to be relevant except in the context of changes in gnathostome global diversity. In addition, we downloaded all available genus-level and species-level occurrences of conodonts (all euconodonts by default as paraconodonts went extinct before this interval) from the Paleontology Database (PBDB) (*19*) All conodont records were downloaded on 30 September 2025 (data S6 and S7). This resulted in a total of 613 stage-binned genus-level records and 1398 species-level records for the Ordovician-Silurian (Figs. 1, A and B, and 3, A and B). We note that many genus-level records lacked assigned species (“species not entered”) as of September 2025, and some genera had no species, despite type species being a requirement for taxonomic validity. This may be due to the inclusion of genus-level information from Sepkoski’s compendium (*6*) and uneven species-level entry efforts. Because of these issues, we augmented our PBDB data with later genus-level stage-binned records from Sepkoski’s compendium (*6*) to fit Silurian diversity trends in the context of the later record for the clade until their end-Triassic extinction (*2*).

### Taxonomic group assignments for occurrences

We classified each gnathostome record in each fauna into 1 of 12 categories representing major gnathostome lineages or groups (*10*) (data S1), based on their assignments in the literature including seven Paleozoic jawless gnathostome classes (Arandaspida, Astraspida, Thelodonti, Galeaspida, Osteostraci, Heterostraci, and Anaspida), one group of additional Ordovician lineages falling outside established groups (“other Pteraspidimorpha”), and four Paleozoic jawed gnathostome classes (“Chondrichthyes,” “Acanthodii,” “Placodermi,” and “Osteichthyes”). In addition, we created an “Unknown” category for taxa whose exact classification remain undetermined, disputed, or controversial in the literature but are distinct from co-occurring species and have valid genus names. Of the other categories, two may be paraphyletic or polyphyletic (other Pteraspidimorpha and Acanthodii) but represent distinct sets of similar species and have been used as historical categories. We explain the usage and assignment of “Unknown,” “other Pteraspidimorpha,” “Acanthodii,” “Chondrichthyes,” and “Placodermi” in further detail below.

We primarily used the “Unknown” category in this study for enigmatic microfossil taxa from the Ordovician and Silurian. This category included *Skiichthys halsteadi* and *Eleochera glossa* from our “Winnipeg Formation” and “Canon City” faunas in the United States (*39*, *40*) (data S1). The former species has been suggested to have affinities with Acanthodii or Placodermi (*8*), but its classification remains uncertain (*41*). The latter species exhibits affinities with Chondrichthyes or Anaspida; however, its precise taxonomic placement has not been determined (*42*). In addition, Ordovician taxa identified from microfossil records and previously attributed to “Chondrichthyes,” such as those in the “Stokes Formation” and “Winnipeg Formation” faunas, were also reassigned to the “Unknown” category. This was due to reconsideration and uncertainty expressed by the same authors that made the original assignments in subsequent papers, as well as doubts expressed by other workers in published sources (*3*, *10*) (data S1). The exceptions are taxa from “Canon City” and *Tantalepis* from the “Stairway Sandstone,” which have been classified as stem-Chondrichthyes continuously in the literature and by the same authors, despite some minor uncertainty (*3*, *10*, *20*, *42*, *43*) (see discussion of “Chondrichthyes” below). *Tesakoviaspis concentrica*, a scale-based taxon recovered from “Tchunia River” and “Moyerochan Formation” in Siberia during the latest Ordovician to earliest Silurian, was also classified as “Unknown.” Although it was previously assigned as Astraspida (*6*), histological evidence has raised doubts about this placement (*42*, *44*). We excluded *Dictyorhabdus priscus* from “Canon City” (*45*, *46*) from the dataset as it has not been confirmed to be a true vertebrate, despite suggestions of a weak affinity with other gnathostomes (*47*, *48*).

“Other Pteraspidomorpha” was used for the Ordovician taxa *Pircanchaspis rinconensis* (*49*), *Pycnaspis splendens*, and *Pycnaspis* sp. cf. *splendens* (*41*), as their placement in relation to Arandaspida and Astraspida remains unresolved, and they may represent distinct groups of Ordovician gnathostomes (see above). Arandaspida, Astraspida, and Heterostraci are here treated separate groups because they have been regarded as subclasses within Pteraspidomorpha (*10*, *37*). In addition, recent histological and phylogenetic work on arandaspids and astraspids has found that these were both sister groups to the exclusively Silurian Heterostraci and has further highlighted the distinctions between Arandaspida and Astraspida (*33*, *34*, *47*). Therefore, we decided to treat these subclasses as distinct groups in line with a previous work (*10*).

“Acanthodii” were previously considered a distinct class and later a polyphyletic set of differentiated clades (e.g., Ischnacanthida, Acanthodida, Gyracanthida, and Climatiida) with affinities to chondrichthyans, stem-gnathostomes, and osteichthyans (*2*, *17*). The discovery of the maxillate placoderm *Entelognathus* changed early gnathostome phylogeny such that acanthodians became a set of stem-chondrichthyan clades with distinct sets of characters (*50*). Although a number of Silurian and Ordovician scale and spine-based taxa have been assigned as stem-“chondrichthyans” without clarification (*7*, *11*, *25*), some of the same authors have also recently assigned early Silurian body fossils as “acanthodian-grade” on the basis of a body plan shared with the older classification and a body covering distinct from isolated scale forms (*25*). All stem-chondrichthyan, non-acanthodian taxa in our dataset lack body fossils, whereas “Acanthodii” seems to represent a diagnostic set of ecologically distinct lineages or a grade of advanced “stem-Chondrichthyans” with both body fossils and diagnostic spines from throughout the Silurian-Permian (*2*, *10*, *17*, *18*).

Given that the ongoing alternative use of “Acanthodii” and “stem-Chondrichthyes” in Ordovician-Silurian taxonomic surveys seems to be based on real differences in ecomorphology and ancestry, we have decided to assign our taxa to each group following their attributions in the literature. That said, it is still possible that some of these distinctions are false given that attribution tends to be based on different kinds of fossils. Furthermore, it is possible that Ordovician scale-based “Chondrichthyes” taxa represent one or several distinct Ordovician lineages given differences with Silurian ichthyoliths assigned to the same group (see Discussion). To understand the influence of these classifications on our results, we have performed supplementary ecological reanalyses using three additional alternative groupings for fossils attributed to total group Chondrichthyes: (i) All Ordovician Chondrichthyes are “unknown,” and Acanthodians and “Chondrichthyes” are separate Silurian groups; (ii) All acanthodians and stem-chondrichthyans are “Chondrichthyes”; and (iii) Ordovician “Chondrichthyes” are unknown, and all acanthodians are “Chondrichthyes.” The results of these alternative analyses are presented in the supplementary results (figs. S11 to S17).

“Placodermi” is a category of jawed stem-gnathostome that has a similar history of usage to that for Acanthodii. Originally, Placodermi was considered to be a monophyletic clade outside the gnathostome crown but is now thought to represent a polyphyletic grade of stem lineages (e.g., Arthrodira, Antiarchi, Rhenanida, Ptyctodontida, Phyllolepida, and others) stretching from the origin of jaws to the origin of the crown (*2*, *17*, *51*). As placoderms have body plans that are conserved within their clades and distinct from most crown lineages, and have relatively few records in the Silurian, we have decided to treat their occurrences as belonging to a single group.

### Age and environmental assignments for faunas

We determined ages and geological stage assignments for our Ordovician-Silurian faunas based on the latest available stratigraphic data from the literature for the geological information taken from the original description or survey. In several cases, this resulted in changes in the estimated age from that reported in the early vertebrate descriptive literature, due to updated stratigraphy (see above). We listed all specific age references for each fauna in data S1. Stratigraphic nomenclature and dates adhered to the “International Chronostratigraphic Chart” (December 2024) (*9*). We assigned the same age to all species occurrences within a particular fauna. We gave each fauna a distinct name using its most commonly recognized designation, typically derived from a nearby settlement or landmark (data S1). In cases where multiple faunas were confirmed in the same general area or from different times within a continuous section, we used the name of the exact formation yielding the specimen. There were cases where the fauna could not be assigned to a single stage, given the existence of boundary-crossing faunas and formations or uncertainty about exact location within a formation spanning multiple intervals. Consequently, when calculating the total richness for each stage, we counted the occurrences within these faunas in multiple stages (Fig. 1, figs. S4 and S5, tables S4 and S6). However, when assigning faunas to a single time interval for the purposes of ordination, we chose the stage with the longer duration according to the “International Chronostratigraphic Chart” (*9*) (Figs. 2 and 4A and tables S1 and S2).

We verified the locality and geological information for faunas using the same approach as that for age determination, following Sallan and Coates (*17*). In addition, to interpret the distribution of Paleozoic gnathostomes, we assigned each fauna to one of five distinct geographic regions based on its locality: “Gondwana,” “Siberia,” “Laurasia,” “Baltic,” and “China” (Figs. 1 to 3 and figs. S5 and S6C). We subsequently converted these categorical region labels to numeric values (1 to 5) for use in statistical analyses such as CCA and NMDS (see below).

We used evidence from the geological literature or coincident invertebrate faunas to estimate the environment of our faunas (data S1). We used Benthic Assemblage Zones as a coding scheme spanning from fresh water (BA0) to open ocean (BA6), following the procedure of Sallan *et al.* (*10*). The Benthic Assemblage Zones for many of our faunas were first determined by Boucot and Janis (*52*) or in Sallan *et al.* (*10*). For other faunas without known habitat assignments, we referred to the literature as described in the supplemental dataset (data S1). After we assigned each fauna a Benthic Assemblage Zone (data S1), we then simplified these into three environmental categories: “fresh water,” “brackish,” and “marine.” Faunas assigned as only BA0 were treated as “fresh water,” whereas those in which the fauna expanded into marine waters as well as BA0 were assigned as “brackish.” All other BA values were treated as “marine.” These classifications were also subsequently converted into numeric values (1 to 3) for use in statistical analyses (Fig. 2, B and C; figs. S6B, S7, S12, and S15; and tables S8 and S13). For some statistical analyses, we combined the first two categories in a single “nonmarine” category (Fig. 2, B and C, and fig. S6B).

### Datasets used for statistical analyses of faunal composition

After compiling the raw occurrence and faunal data in data S3 and S4, we compiled the details for each fauna in data S1, which represented our master list of Ordovician-Silurian faunas. Details include age, formation, locality, environment, the resident species assigned to each major gnathostome group, and sources (data S1). On the basis of the age and taxonomic information for our faunas, we first counted species-level occurrences within each stage, whereas we gave special attention to faunas that could not be precisely assigned to a single geological stage, either because of boundary crossing or lack of resolution for the exact age of the fossil-bearing units in the geological literature to date (table S1). We identified a total of 68 of 169 faunas as covering multiple stages and listed these as such in data S1. We counted species occurrences in multistage faunas separately in each relevant time interval for the purposes of analyses requiring time binning. We also counted taxonomic richness at the genus level for each included time interval (table S4); these data are highlighted in red in data S3 and S4. We counted 68 multistage faunas as occurring within each of their stages to calculate the number of faunas per stage in table S5.

For analyses and comparisons of faunal composition, we first created a matrix with the faunas as rows and the major gnathostome groups as columns. Because of the quality of the fossil record, we input the number of species within groups at each fauna, rather than the absolute abundance or occurrences within species as is more routine in modern ecological analyses (*17*, *53*). In using this approach, we followed the justifications laid out by Sallan and Coates (*17*). For example, changes in relative species richness within groups across faunas are reflective of changes in the population levels and relative diversity of those groups given long enough time intervals (*17*). In addition, unlike for the invertebrate fossil record, workers do not routinely record abundances for vertebrate fossils at Paleozoic localities and do not record each field sample as a separate collection (*2*, *17*), making it difficult to reconstruct faunas based on proportions of samples or perform subsampling analyses to control for collection effort. We included our faunal matrix of species within larger taxonomic groups at each fauna as data S2.

We used our faunal matrix as the basis for generating histograms showing richness within major gnathostome groups in faunas from the Ordovician to the Silurian periods (Figs. 2A and 4A). In addition, we created an expanded matrix containing variables such as age, environment, and regional information, which is presented as data S8, to perform ordination analyses and other statistical tests of the relationships between these variables and relative richness within gnathostome groups. This second matrix excludes faunas from data S2 with less than three resident species to ensure robustness in the subsequent analysis, following the reasoning of Sallan and Coates (*17*). We used the matrix in data S8 as the basis for generating all of the multivariate ecological analyses conducted in this paper such as Shannon-Wiener index assessment, hierarchical clustering, CCA, NMDS, factor analysis (FA), one-tailed analysis of similarity (ANOSIM), and similarity percentage (SIMPER), described in detail below (Fig. 2B, figs. S3 to S17, and tables S7 to S15) (*17*, *52*).

### Simple visualization methods

In this study, we used four basic data visualization methods to observe changes in taxon richness through time: stage-binned genus-level diversity curves (Figs. 1 and 4, B and C, and figs. S4 and S5), stage-binned species-level diversity curves (fig. S3), species-level faunal histograms (Figs. 2A and 4A), and regional species-level pie charts (Fig. 3). All of these were generated on the basis of the datasets provided in the Supplementary Materials (tables S1 to S6 and data S1 to S7). For our main diversity curves and interpretations, we used genus-level data in keeping with the standard practices of paleobiological diversity curves, the use of such in mass extinction studies, as well as large fossil databases (*6*, *10*, *17*, *54*). The use of genus-level curves helps avoid uncertainty and noise from changing taxonomic practices as genus-level assignments are more robust to the biases in sampling and naming biases (*55*–*58*). Paleontologists and morphological taxonomists generally diagnose genera based on distinct combinations of characters. As a result, genus names are more stable through time such that fossil morphology–based genera are more comparable with living genera with molecular support (*58*–*60*). For our dataset of gnathostome occurrences, the preferential use of genus-level data for diversity curves also allowed the inclusion of genus-level occurrences that do not have species assignments, a common issue in the Paleozoic given the lack of taxonomic work for some groups of gnathostomes relative to the number of available specimens (*2*). As noted above, there are larger gaps in the conodont species-level data in the PBDB (*19*) such that some valid genera lack any species records in the database as of September 2025 and many genus-level occurrences are marked as “no species entered” (data S6 and S7). This may be because PBDB conodont genus-level records are built on the core of Sepkoski’s compendium (which lacked species assignments) (*6*), because species have not been reported in the literature, or because species attributions at specific localities are unclear as with gnathostomes. In any case, it means the conodont genus-level data are likely less biased by data availability.

We generated genus-level diversity curves for the following groups across four comparisons: (i) Ordovician-Silurian gnathostomes (*N* = 418) versus conodonts (*N* = 613) (Fig. 1, A and B), (ii) 13 groups of Ordovician-Silurian gnathostomes (Fig. 1C and fig. S4), (iii) Ordovician-Silurian gnathostomes in five geographic regions (Fig. 1D and fig. S5), and (iv) Silurian-Devonian jawless gnathostomes (five groups) versus jawed gnathostomes (four groups) (Fig. 4, B and C). We calculated taxonomic richness within each group in each stage at the genus level to create the diversity curves (table S4). For our conodont versus gnathostome and jawed versus jawless comparisons, we also calculated per million-year diversity rate within stage bins by dividing the raw genus-level richness by the duration of each stratigraphic stage, as defined in the “International Commission on Stratigraphy” (Figs. 1B and 4C and figs. S4, C and D, and S5, C and D) (*9*). This helped correct the overall diversity curves and overcome some of the effects of time and sampling differences. As above, in cases where the best age estimate for a fauna spanned multiple stages, we counted resident taxa once in each stage bin. The above issues with the use of species-level data aside, given that our gnathostome occurrence compendium is original to this study and we mark species within faunas, we generated species-level curves for gnathostomes, gnathostome groups, and conodonts for comparison with our genus-level patterns (fig. S3, A to D). In our diversity curve plots, we added black lines at the beginning and end of the Hirnantian to indicate mass extinction/glaciation pulses during Ordovician (*14*).

We generated two histograms to estimate diversity trends across faunas through time using our matrix of number of species within each of 13 groups at each fauna (Fig. 2A), which we then recategorized into jawed and jawless gnathostomes based on the characteristics of their assigned group (Fig. 4A). For the jawed-jawless gnathostome fauna histogram, we omitted species within the Unknown category. On the *x* axis, we arranged all faunas (*N* = 169) in loose chronological order based on their estimated ages (data S1). Here, we assigned faunas spanning multiple stages to the one with the longer duration based on the “International Chronostratigraphic Chart” (*9*) while we ordered faunas of the same age alphabetically. The *y* axis represented species-level richness within each fauna (range 1 to 52) (Fig. 2A).

We used our faunal data and matrix to create pie charts for species-level diversity within each era for five regions to infer rough biogeographic patterns and dispersal (Fig. 3) and test hypotheses such as the “Out of Gondwana” model (*21*, *24*, *61*, *62*). In our main figures, each chart was placed on a era-specific paleomap (2016 Colorado Plateau Geosystems) (*63*) near the center or main gnathostome fossil-bearing area of each paleocontinent, also representing one of the five major Paleozoic regions described by Blakey (*63*).

### Multivariate ecological methods

To compare the overall diversity of faunas within each stage, we calculated three diversity indices (species count, Shannon-Wiener diversity, and taxonomic richness) based on species occurrences within stages and within faunas (table S7) (*53*, *64*). The Shannon-Wiener index assesses both richness and evenness within each fauna, whereas taxonomic richness does not consider evenness (*53*).

We visualized and analyzed the species per group composition of each fauna using multivariate ecological methods and ordinations (*53*). As above, faunas containing only one or two species were omitted. The remaining dataset of 101 faunas was analyzed using R (version 4.4.2) (*65*) with the vegan package (version 2.6-10) (*66*) and PAST (version 1.0.6) (*67*). The applied multivariate methods included hierarchical clustering, CCA, NMDS, FA, ANOSIM, and SIMPER (*52*). We performed all ecological analyses using raw species counts or presence-absence within groups as low absolute diversity for some faunas made it difficult to use relative species diversity without introducing bias (data S8).

To determine whether faunas clustered by age, environment, or region, we used hierarchical clustering analysis to construct a dendrogram based on a distance or similarity matrix, without predefined grouping (*24*). The dendrograms were generated using the hclust function of stats package in R, using the average linkage clustering (UPGMA) method (*68*). Clustering was based on Bray-Curtis distances calculated via the vegdist function in the vegan package (*52*, *65*). We color coded sites according to age, environment, and region (fig. S6).

CCA allows us to visualize the distribution of faunas without requiring a priori grouping. This ordination analysis detects gradients in faunas based on associations between site composition and habitat or other variables such as time. It also allows taxonomic units and faunas to be plotted together, showing the influence of specific groups on the distribution of sites (*53*, *68*). Previous studies (*2*, *10*, *17*) found a relationship between vertebrate faunal composition and habitat depth and showed that CCA can detect real and statistically significant differences between faunas across time. Thus, we used two explanatory variables, geological series and environment, for CCA, which we generated by using the *cca* function in the *vegan* package in R (Fig. 2B and figs. S7, S11, S12, and S15). In the taxon ordination plot (Fig. 2B, right), the direction of the two explanatory variables is shown as black dashed arrows (biplot arrows). In our main figures, we highlighted only the eight taxonomic groups with the highest contributions to the ordination to enhance figure clarity, whereas all we omitted the biplot arrows for all other taxa from the plot for simplicity.

We used a nonconstrained ordination method, NMDS, to visualize the distribution of faunas as this analysis does not assume a gradient or require environmental or other age variables unlike CCA. It also does not assume a normal distribution of samples unlike principal components analysis (PCA) (*53*, *68*). This method visualizes the dataset in a reduced dimensional space while preserving the rank order relationships within the data and is calculated from a dissimilarity matrix. Initially, we applied Bray-Curtis abundance distance and Kulczynski presence-absence similarity metrics to assess differences in the faunal dataset spanning the Ordovician to Silurian periods (Fig. 2C and figs. S8, S9, S11, S13, S14, S16, and S17). In addition, we conducted comparisons between successive series intervals: Middle Ordovician and Upper Ordovician, Upper Ordovician and Llandovery (Silurian), Llandovery and Wenlock (Silurian), Wenlock and Ludlow (Silurian), and Ludlow and Pridoli (Silurian) (fig. S7). We performed these analyses using the *metaMDS* function of the *vegan* package in R (*66*). We calculated the ordination in two dimensions (*k* = 2), with trymax set to 100 to ensure convergence to a stable solution. A random seed was set [set.seed(123)] to ensure reproducibility. We monitored the stress values to assess the quality of the ordination.

We used FA to identify key taxa contributing to faunal differentiation through visualization and detect breaks across the mass extinction following prior usage by Raup and Sepkoski (*17*, *53*) (figs. S10 and S11 and table S9). This method allows us to identify hidden common factors that drive the variation among observed taxa. By focusing on the covariance structure, FA highlights the underlying patterns that influence the data, rather than merely accounting for all the variance observed in the dataset (*52*, *53*). The analysis follows a similar procedure to PCA but with a fundamental distinction that will be discussed below. Whereas PCA seeks to identify principal components that account for the maximum variance within a dataset by considering the total variance, FA is specifically designed to extract common factors based solely on the covariance structure among variables (*17*, *53*). This approach enables us to unveil latent factors that may influence the distribution of taxa, helping to interpret the ecological processes driving community differentiation. We performed FA using PAST (*67*).

To determine whether differences or similarities between faunas from different geological series shown in our ordinations were statistically significant, we used ANOSIM using pairwise comparisons of all series (*17*, *53*). This analysis uses permutations of samples within two bins to determine an R statistic showing the degree of similarity or difference among the compared populations within those bins and the significance of the same. We used 1 million permutations for each pairwise comparison between all gnathostome faunas from each Ordovician-Silurian geological series, generating *P* values (α = 0.05) and R statistics (table S10). We used SIMPER, which compares the relative contribution of taxonomic groups to differences between groups of sites, to interpret the results of ANOSIM and to quantify the relative contribution of taxonomic groups to observed differences between intervals (tables S11 and S12). We applied ANOSIM by using the *anosim* function in the *vegan* package in R, and SIMPER was generated by using PAST (*65*–*67*). All data and detailed technical results are included in the Supplementary Materials.

## Supplementary Materials

### The PDF file includes:

Supplementary Text Figs. S1 to S17 Tables S1 to S14 Legends for data S1 to S8 References
