# Supplementary material for: Mass extinction triggered the early radiations of jawed vertebrates and their jawless relatives (gnathostomes)
Source: Sci Adv. 2026 Jan 9;12(2):eaeb2297. doi: 10.1126/sciadv.aeb2297 (PMC12787538; doi:10.1126/sciadv.aeb2297)
Supplement: Supplementary file 2 — Data S1 to S8 [file sciadv.aeb2297_data_s1_to_s8.zip › Supplementary Data Files/Data_S1_Faunalist Final.docx]

**A. Ordovician sites**

**1. Pacoota Sandstone**
**Stratigraphy/Location.** Pacoota Sandstone Formation, Amadeus Basin/ Western end of Gardiner Range, on southern limb of Gardiner Range Anticline, Central Australia. **Age.** Tremadocian–Floian.
**Environment.** BA1
**Taxa.**
Arandaspida: Porophoraspis sp..
**Reference.** (1), (2), (3), (4)
**Age References.** (2), (3), (4)
**Env References.** (1)
**Taxa References.** (1)

**2. Horn Valley Siltstone**
**Stratigraphy/Location.** Horn Valley Siltstone Formation, Amadeus Basin/ Eastern Gardiner Range, Areyonga Creek Section, Northern Territory, Australia. **Age.** Early Floian.
**Environment.** BA1–2
**Taxa.**
Arandaspida: Porophoraspis sp..
**Reference.** (1), (2), (3)
**Age References.** (3)
**Env References.** (2)
**Taxa References.** (1) **3. Rincon**
**Stratigraphy/Location.** Pircancha Formation/ Rincon, Dscuela Seccional de Puesto Grande, Tarija, Bolivia.
**Age.** Floian.
**Environment.** BA1–2
**Taxa.**
Other Pteraspidomorpha: Pircanchaspis rinconensis.
**Reference.** (3), (5), (6)
**Age References.** (3)
**Env References.** (6)
**Taxa References.** (5)

**4. Wadi Daiqa**
**Stratigraphy/Location.** Amdeh Formation Am4, DX3A/ Wadi Daiqa, Oman. **Age.** Latest Dapingian?–Early Darriwilian.
**Environment.** BA1–2
**Taxa.**
Arandaspida: Sacabambaspis sp..
**References.** (3), (6), (7)
**Age References.** (7)
**Env References.** (7)
**Taxa References.** (3), (6)

**5. Wadi Qahza**
**Stratigraphy/Location.** Amdeh Formation Am5/ Wadi Qahza, Oman.
**Age.** Llanvirn Darriwilian. (8)
**Environment.** BA1–2 (8)
**Taxa.** (6), (7)
Arandaspida: Sacabambaspis sp..
**References.** (6), (7), (8)
**Age References.** (8)
**Env References.** (8)
**Taxa References.** (6), (7)

**6. Hayl al Quwasim**
**Stratigraphy/Location.** Amdeh Formation Am5/ Hayl al Quwasim, Oman.
**References.** (7), (8) **Age.** 462–463 Ma, Darriwilian. (7), (8)
**Environment.** BA1–2 (7), (8)
**Taxa.** (7), (8)
Arandaspida: Sacabambaspis sp..
**References.** (7), (8)
**Age References.** (7), (8)
**Env References.** (7), (8)
**Taxa References.** (7), (8)

**7. Al Fleij**
**Stratigraphy/Location.** Amdeh Formation Am5/ Al Fleij, Oman.
**References.
Age.** 458–460 Ma, Late Darriwilian.
**Environment.** BA1–2? 2–3?
**Taxa.**
Arandaspida: Sacabambaspis sp..
**References.** (7)
**Age References.** (7)
**Env References.** (7)
**Taxa References.** (7)

**8. Stairway Sandstone**
**Stratigraphy/Location.** Stairway Sandstone, Amadeus Basin/ James ‘B’ Anticline, Mt Watt, Mt Charlottea and Maloney Hill, Northern Territory, Australia. **Age.** Early–Middle Darriwilian.
**Environment.** BA1–2
**Taxa.**
Arandaspida: Arandaspis prionotolepis, Arandaspis sp., Porphoraspis crenulata, Porphoraspis sp..
Chondrichthyes: Tantalepis gatehousei.
**References.** (1), (3), (9), (10), (11), (12), (13)
**Age References.** (3)
**Env References.** (11), (12)
**Taxa References.** (1), (9), (10), (13)

**9. Angara River**
**Stratigraphy/Location.** Mamyry Formation/ Angara River, Irkutsk Basin of Siberia, Russia.
**References.**  **Age.** Late Darriwilian.
**Environment.** BA1–2
**Taxa.**
Astraspida: Kodinskaspis angarensis.
**References.** (14)
**Age References.** (14)
**Env References.** (14)
**Taxa References.** (14)

**10. Sepulturas Formation**
**Stratigraphy/Location.** Sepulturas Formation/ West of Alto del Condor, Jujuy Province, Argentina. **Age.** Late Darriwilian.
**Environment.** BA2
**Taxa.**
Arandaspida: Sacabambaspis janvieri.
**References.** (3), (14)
**Age References.** (3)
**Env References.** (14)
**Taxa References.** (14) **11. Split Rock**
**Stratigraphy/Location.** Rowena Formation/ Split Rock, Mutawintji National Park, NSW, Australia. **Age.** Darriwilian.
**Environment.** BA0–1?
**Taxa.**
Arandaspida: Arandaspis sp.
**References.** (3), (15), (16)
**Age References.** (3)
**Env References.** (16)
**Taxa References.** (15) **12. Winneshiek Lagerstätte**
**Stratigraphy/Location.** St. Peter Formation/ Winneshiek Lagerstätte, Decorah, Iowa, USA. **Age.** Darriwilian.
**Environment.** BA1
**Taxa.**
Astraspida: Astraspis sp..
**References.** (17), (18)
**Age References.** (17)
**Env References.** (18)
**Taxa References.** (17)

**13. Stokes Formation**
**Stratigraphy/Location.** Stokes Formation, Amadeus Basin/ Areyonga Creek, Northern Territory, Australia. **Age.** Late Darriwilian.
**Environment.** BA1–2
**Taxa.**Arandaspida: Sacabambaspis sp..
Astraspida: Apedolepis tomlinsonae.
Unknown: Areyongalepis oervigi, Indet.
**References.** (1), (2), (3), (11), (12), (13), (19)
**Age References.** (2), (3), (11)
**Env References.** (11), (12)
**Taxa References.** **(1), (13), (19)**

**14. Carmichael Sandstone**
**Stratigraphy/Location.** Carmichael Sandstone, Amadeus Basin/ Johnny's Creek Anticline, Northern Territory, Australia. **Age.** Sandbian.
**Environment.** BA2 (12)
**Taxa.** (1)
Arandaspida: Sacabambaspis sp..
**References.** (1), (2), (3), (12)
**Age References.** (2), (3)
**Env References.** (12)
**Taxa References.** **(1)**

**15. Trapiche Group**
**Stratigraphy/Location.** La Cantera Formation/ Sierra de Villicum, San Juan Province, Argentina. **Age.** Sandbian.
**Environment.** BA1–2
**Taxa.**
Arandaspida: Sacabambaspis janvieri.
**References.** (3), (6), (20), (21)
**Age References.** (3)
**Env References.** (6)
**Taxa References.** **(20), (21)**

**16. Winnipeg Formation**
**Stratigraphy/Location.** Winnipeg Formation/ Bighorn Mountains, Northern Central Wyoming; Shell Pine Unit No.1, Wibaux County, Williston Basin, Eastern Montana, USA; Murphy E Poplar 1, Roosevelt County, Williston Basin, Eastern Montana, USA. **Age.** Sandbian–Katian.
**Environment.** BA1–2
**Taxa.**
Other Pteraspidomorpha: Pycnaspis splendens, Pycnaspis cf. splendens.
Astraspida: Astraspis desiderata, Eriptychiidae americanus, Eriptychiidae sp.
Unknown: Eleochera glossa, Shark-like scalemorph, Skiichthys halsteadi.
**References.** (12), (13), (22), (23), (24), (25), (26), (27)
**Age References.** (25)
**Env References.** (12)
**Taxa References.** **(13), (22), (23), (24)**

**17. Sand Point**
**Stratigraphy/Location.** Lowville Formation/ Sand Point, Ontario, Canada.
**References.**  **Age.** Sandbian–Katian.
**Environment.** BA1–2
**Taxa.**
Astraspida: Astraspis sp..
**References.** (28), (29)
**Age References.** (28)
**Env References.** (29)
**Taxa References.** **(28)**

**18. St Joseph Island**
**Stratigraphy/Location.** Gull River Formation/ St Joseph Island, Ontario, Canada. **Age.** Sandbian–Katian.
**Environment.** BA1
**Taxa.**
Astraspida: Astraspis desiderata, Eriptychius sp..
Thelodonti: Larolepis darbyi.
**References.** (24), (30), (31), (32)
**Age References.** (24)
**Env References.** (32)
**Taxa References.** (30), (31)

**19. Canon City**
**Stratigraphy/Location.** Harding Formation/ Canon City, Colorado, USA.
**Age.** Sandbian–Katian. (24),
**Environment.** BA0–2? (12),
**Taxa.**
Astraspida: Astraspis desiderata, Astraspis sp., Eriptychiidae americanus.
Thelodonti: Indet.
Chondrichthyes: Canyonlepis smithae, Solinalepis levis, Tezakia hardingensis, Indet.
Unknown: Eleochera glossa, Skiichthys halsteadi.
**References.** (12), (13), (24), (26), (27), (33), (34), (35), (36), (37), (38), (39), (40), (41), (42)
**Age References.** (24), (34), (35)
**Env References.** (12), (35), (39)
**Taxa References.** **(13), (24), (26), (27), (33), (36), (37), (38), (40), (42), (42)**

**20. Arbuckle Mountains**
**Stratigraphy/Location.** Viola Limestone/ Arbuckle Mountains, Southern Oklahoma.
**Age.** Katian.
**Environment.** BA2–3
**Taxa.**
Astraspida: Eriptychiidae americanus.
**References.** (12), (24), (43), (44), (45), (46)
**Age References.** (24)
**Env References.** (12), (45), (46)
**Taxa References.** (43), (44)

**21. Basal Trenton Group**
**Stratigraphy/Location.** Basal Trenton beds/ La Malbaie, on the north shore of the St. Lawrence River in Quebec, Canada.
**Age.** Katian.
**Environment.** BA1
**Taxa.**
Astraspida: Astraspis sp..
**References.** (47), (48)
**Age References.** (48)
**Env References.** (48)
**Taxa References.** (47)

**22. Canning Basin**
**Stratigraphy/Location.** Nibil Formation/ Subsurface Kidson 1 core, Western Australia.
**Age.** Katian.
**Environment.** BA1–2
**Taxa.**
Astraspida: Richieichthys nibili.
**References.** (2)
**Age References. (2)**
**Env References. (2)**
**Taxa References. (2)**

**23. Cochabamba**
**Stratigraphy/Location.** Anzaldo Formation/ Cochabamba Province, Bolivia.
**Age.** Katian.
**Environment.** BA1–2
**Taxa.**
Arandaspida: Sacabambaspis janvieri.
**References.** (3), (49), (50), (51)
**Age References. (3)**
**Env References. (3)**
**Taxa References. (49), (50), (51)**

**24. Ouareau River**
**Stratigraphy/Location.** Upper-most Pamelia Formation/ Ouareau River, Quebec, Canada.
**Age.** Katian.
**Environment.** BA1
**Taxa.**
Astraspida: Astraspis sp. or desiderata?
**References.** (48), (52)
**Age References. (48)**
**Env References. (48)**
**Taxa References. (52)**

**25. Ozernaya Formation**
**Stratigraphy/Location.** Upper Ozernaya Formation/ Strojnaya River, October Revolution Island, Russia.
**Age.** Katian.
**Environment.** BA1–2
**Taxa.**
Thelodonti: Stroinolepis maenniki.
**References.** (53), (54)
**Age References. (53)**
**Env References. (54)**
**Taxa References. (53)**

**26. Pont-Rouge**
**Stratigraphy/Location.** Lower Leray Formation–Lowville Formation/ Pont-Rouge, Quebec, Canada.
**Age.** Katian.
**Environment.** BA1
**Taxa.**
Astraspida: Astraspis sp. or desiderata?
**References.** (48), (52)
**Age References. (48)**
**Env References. (48)**
**Taxa References. (52)**

**27. Whitewood Formation**
**Stratigraphy/Location.** Whitewood Formation, Bighorn Group/ Sheep Mountain, Spearfish Canyon, Black Hills region, South Dakota, USA.
**Age.** Katian.
**Environment.** BA1–2
**Taxa.**
Astraspida: Astraspis sp., Astraspis desiderata.
Other Pteraspidomorpha: Pycnaspis cf. splendens.
**References.** (12), (22), (25)
**Age References. (25)**
**Env References. (12)**
**Taxa References. (22)**

**28. Timan1**
**Stratigraphy/Location.** Khoreiver and Muker Formation/ Timan-Pechora, Russia.
**Age.** Katian–Hirnantian.
**Environment.** ?
**Taxa.**
Thelodonti: Sandivia augusta, Sandivia melnikovi.
**References.** (31), (53), (55), (56)
**Age References. (53), (56)**
**Env References. ?**
**Taxa References. (31), (55)**

**29. Strojnaya River**
**Stratigraphy/Location.** Strojnaya Formation/ Strojnaya River, October Revolution Island, Russia.
**Age.** Hirnantian.
**Environment.** BA1–2
**Taxa.**
Thelodonti: Stroinolepis maenniki.
**References.** (53), (54)
**Age References. (53)**
**Env References. (54)**
**Taxa References. (53)**

**30. Tchunia River**
**Stratigraphy/Location.** Unknown/ Tchunia (Tchunku) River, Nizhniaya (Silurian of Russian Federation), Siberian Platform.
**Age.** Hirnantian–Llandovery (Silurian).
**Environment.** ?
**Taxa.**
Unknown: Tesakoviaspis concentrica.
**References.** (57)
**Age References. (57)**
**Env References. ?**
**Taxa References. (57)**

**B. Llandovery Sites 

1. Melichan Formation**
**Stratigraphy/Location.** Melichan Formation, Niuya-Beresovo District, Russia.
**Age.** Llandovery (Rhuddanian), Khaastyrian, Moyerocanian, and Agidyan Regional Stages.
**Environment.** BA0–1
**Taxa.**
Thelodonti: Loganellia asiatica, Loganellia sibirica.
**References.** (58), (59), (60), (61)
**Age References. (58), (61)**
**Env References. (60)**
**Taxa References. (59), (61)**

**2. Kyzyl-Tchiraa**
**Stratigraphy/Location.** Alash and Kyzyl-Tchiraa (Charaa) Horizon, Elegest Formation/ Kyzyl-Tchiraa, Tuva, Russia.
**Age.** Llandovery (Rhuddanian–Aeronian), Alash and Kyzyl-Tchiraa Regional Stages.
**Environment.** BA1–3?
**Taxa.**
Thelodonti: Loganellia asiatica, Loganellia sibirica, Talimaalepis kadvoiensis.
Acanthodii: Tchunacanthus obruchevi.
**References.** (57), (58), (61), (62), (63), (64)
**Age References. (58), (61), (63)**
**Env References. (58), (63)**
**Taxa References. (57), (62), (64)**

**3. Moyerochan Formation**
**Stratigraphy/Location.** Moyerochan Formation/ Moyero River, Moyerocanian, Krasnoyarsk Territory, Russia.
**Age.** Llandovery (Rhuddanian and Aeronian).
**Environment.** BA1–3?
**Taxa.**
Thelodonti: Angaralepis moskalenkoae, Loganellia asiatica, Loganellia sibirica.
Acanthodii: Lenacanthus priscus, Tchunacanthus obruchevi.
Chondrichthyes: Elegestolepis conica.
Unknown: Tesakoviaspis concentrica.
**References.** (53), (57), (63), (65), (66), (67)
**Age References. (63)**
**Env References. (63)**
**Taxa References. (53), (57), (65), (66), (67)**

**4. Petit River Port Daniel**
**Stratigraphy/Location.** Petit River Port Daniel/ Chaleur Bay, Clemville, Quebec, Canada.
**Age.** Llandovery (Aeronian and Rhuddanian).
**Environment.** BA1–
**Taxa.**
Thelodonti: Valyalepis crista.
**References.** (68)
**Age References. (68)**
**Env References. (68)**
**Taxa References. (68)**

**5. Door County**
**Stratigraphy/Location.** Hendricks Formation/ Door County, Wisconsin, USA.
**Age.** Llandovery (Aeronian).
**Environment.** BA1
**Taxa.**
Thelodonti: Valyalepis crista.
**References.** (68), (69)
**Age References. (69)**
**Env References. (69)**
**Taxa References. (68)**

**6. Fayette State Park**
**Stratigraphy/Location.** Hendricks Formation, Burnt Bluff Group/ Fayette State Park, Michigan, USA.
**Age.** Llandovery (Aeronian).
**Environment.** BA1
**Taxa.**
Thelodonti: Zuegelepis potanus.
**References.** (69)
**Age References. (69)**
**Env References. (69)**
**Taxa References. (69)**

**7. Hadeland**
**Stratigraphy/Location.** Upper Saelabonn Formation/ Bjellum, Hadeland, Oslo, Norway.
**Age.** Llandovery (Middle-Aeronian).
**Environment.** BA1–
**Taxa.**
Thelodonti: Loganellia cf. aldridgei.
**References.** (70)
**Age References. (70)**
**Env References. (70)**
**Taxa References. (70)**

**8. Kalana Quarry**
**Stratigraphy/Location.** Jogeva Beds/ Kalana Quarry, Estonia.
**Age.** Llandovery (Aeronian), Raikkula Regional Stage.
**Environment.** BA1–3
**Taxa.**
Osteostraci: Kalanaspis delectabilis.
**References.** (71)
**Age References. (71)**
**Env References. (71)**
**Taxa References. (71)**

**9. Leijiatun Village**
**Stratigraphy/Location.** Rongxi Formation/ Shiqian–Tunping section at Leijiatun Village, Guizhou Province, China.
**Age.** Llandovery (Late Aeronian).
**Environment.** BA1–2
**Taxa.**
Chondrichthyes: Fanjingshania renovate, Qianodus duplicis.
**References.** (72), (73), (74) **Age References.** (72), (73)
**Env References. (74)**
**Taxa References.** (72), (73)

**10. Schoolcraft County**
**Stratigraphy/Location.** Byron Formation, Burnt Bluff Group/ Schoolcraft County, Michigan, USA.
**Age.** Llandovery (Aeronian).
**Environment.** BA1
**Taxa.**
Thelodonti: Zuegelepis potanus.
**References.** (69)
**Age References. (69)**
**Env References. (69)**
**Taxa References. (69)**

**11. Sturgeon Bay**
**Stratigraphy/Location.** Hendricks Formation, Burnt Bluff Group/ Sturgeon Bay, Wisconsin, USA.
**Age.** Llandovery (Aeronian).
**Environment.** BA1–
**Taxa.**
Thelodonti: Zuegelepis potanus.
**References.** (69)
**Age References. (69)**
**Env References. (69)**
**Taxa References. (69)**

**12. Srednii Island**
**Stratigraphy/Location.** Golomyannyj Formation/ Srednii Island, Sedova Archipelago, Russia.
**Age.** Llandovery (Aeronian).
**Environment.** BA1
**Taxa.**
Thelodonti: Loganellia aldridgei, Loganellia matura, Loganellia sibirica.
**References.** (53), (75)
**Age References. (53)**
**Env References. (75)**
**Taxa References. (53)**

**13. Angara-Ilim Region**
**Stratigraphy/Location.** Raasokha Formation?/ Angara-Ilim, Irkutsk Territory, Russia.
**Age.** Llandovery (Telychian and Aeronian).
**Environment.** ?
**Taxa.**
Thelodonti: Loganellia aldridgei, Loganellia asiatica, Loganellia matura, Loganellia scotica, Loganellia sibirica, Loganellia sp. cf. scotica.
**References.** (12), (53), (65), (66), (76), (77)
**Age References. (12), (66), (76), (77)**
**Env References. ?**
**Taxa References. (53), (65), (66)**

**14. Ilim District**
**Stratigraphy/Location.** Raasokha and Talikit Formations/ Ilim District, Russia.
**Age.** Llandovery (Aeronian–Telychian), Moyerocanian, Agidyan, and Khaastyrian Regional Stages.
**Environment.** BA1–3
**Taxa.**
Thelodonti: Angaralepis moskalenkoae, Loganellia asiatica, Loganellia sibirica, Loganellia sp. indet., Talimaalepis kadvoiensis.
**References.** (58), (60), (61)
**Age References. (58), (61)**
**Env References. (60), (61)**
**Taxa References. (58), (61)**

**15. Kochumdek District**
**Stratigraphy/Location.** Kulinna, Kochumdek, and Razvilka Formations/ Lower Tchunku River, Kochumdek District, Pretunguska, Russia.
**Age.** Llandovery (Telychian and Aeronian), Khaastyrian, Moyerocanian and Agidyan Regional Stages.
**Environment.** BA0–1
**Taxa.**
Thelodonti: Angaralepis moskalenkoae, Loganellia aldridgei, Loganellia asiatica, Loganellia matura, Loganellia scotica, Loganellia sibirica, Loganellia sp. cf. L. scotica, Loganellia sp. indet.
**References.** (53), (58), (61), (65), (66), (67), (76), (77), (78)
**Age References. (58),** (61), (66), (76), (77),
**Env References. (58),** (61)
**Taxa References. (53),** (61), (65), (66), (67), (78)

**16. Niuya-Beresovo District**
**Stratigraphy/Location.** Utakan Formations/ Niuya-Beresovo District, Russia.
**Age.** Llandovery (Aeronian–Telychian), Agidyan Regional Stage.
**Environment.** BA0–1
**Taxa.**
Thelodonti: Angaralepis moskalenkoae, Loganellia aldridgei, Loganellia matura, Loganellia scotica, Loganellia sibirica, Loganellia sp. cf. L. scotica.
**References.** (53), (58), (61), (65), (66), (67)
**Age References. (53), (58), (66)**
**Env References. (58)**
**Taxa References. (53), (58), (65), (66), (67)**

**17. Pioneer Island**
**Stratigraphy/Location.** Golomyannyj Formation/ Pioneer Island, Severnaya Zemlya Archipelago, Russia.
**Age.** Llandovery (Aeronian–Telychian).
**Environment.** BA1–2
**Taxa.**
Thelodonti: Paralogania klubovi.
**References.** (53), (54)
**Age References. (53)**
**Env References. (54)**
**Taxa References. (53)**

**18. Shropshire**
**Stratigraphy/Location.** Minsterley, Purple Shales, and Venusbank Formation/ Shropshire, UK.
**Age.** Llandovery (Aeronian and Telychian).
**Environment.** BA3–
**Taxa.**
Thelodonti: Loganellia aldridgei, Loganellia matura, Loganellia scotica, Loganellia sibirica, Loganellia sp. cf. L. scotica.
**References.** (12), (53), (65), (66), (67), (79), (80)
**Age References. (53), (66), (79), (80)**
**Env References. (12)**
**Taxa References. (53), (65), (66), (67), (79)**

**19. Strojnaya River**
**Stratigraphy/Location.** Golomyannyj Formation/ Strojnaya River, October Revolution Island, Severnaya Zemlya Archipelago, Russia.
**Age.** Llandovery (Aeronian-Telychian).
**Environment.** BA1–2
**Taxa.**
Thelodonti: Loganellia aldridgei, Loganellia matura, Loganellia sibirica.
**References.** (53), (54)
**Age References. (53)**
**Env References. (54)**
**Taxa References. (53)**

**20. Qingshui Formation**
**Stratigraphy/Location.** Qingshui Formation/ Lixi Town, Wuning County, Jiangxi Province and Gumushan, Wuhan, Hubei Province, South China.
**Age.** Llandovery (Lower Telychian).
**Environment.** BA1
**Taxa.**
Galeaspida: Jiangxialepis retrospina.
Chondrichthyes: Eosinacanthus shanmenensis, Indet Fin spine morphology1, Indet Fin spine morphology2, Indet Fin spine morphology3, Neosinacanshus planispinatus, Sinacanthus sp., Sinacanthus triangulates, Sinacanthus wuchangenis, Tarimacanthus bachuensis.
**References.** (81), (82), (83)
**Age References. (81)**
**Env References. (82)**
**Taxa References. (81), (83)**

**21. Asker**
**Stratigraphy/Location.** Vik Formation/ Gjettum Station, Asker, Oslo, Norway.
**Age.** Llandovery (Middle–Late Telychian).
**Environment.** BA2–
**Taxa.**
Thelodonti: Thelodus sp., Thelodus parvidens.
**References.** (70), (84)
**Age References. (84)**
**Env References. (84)**
**Taxa References. (70)**

**22. Bachu-Kalpin1**
**Stratigraphy/Location.** Tataaiertage (Tataertag), Yimugantawu and Kezirtag Formations/ Bachu-Kalpin, Tarim Platform, Xinjiang, China.
**Age.** Llandovery (Telychian).
**Environment.** BA0–2
**Taxa.**
Galeaspida: Changxingaspis nianzhongi, Kapinolepis taimensis, Hanyangaspis sp., Jiangxialepis retrospina, Jiaoyu imperfectus, Nanjianaspis kalpinensis, Nanjianaspis sp. cf. zhangi, Pseudoduyunaspis bachuensis.
Chondrichthyes: cf. Elegestolepis, cf. Mongolepis rozmanae, Neosinacanthus planispinatus, Neosinacanthus sp., Neosinacanthus sp.2, Rongolepis cosmetica, Sinacanthus sp., Sinacanthus triangulates, Sinacanthus wuchangensis, Tarimacanthus bachuensis, Xinjiangichthys pluridentatus, Xinjiangichthys tarimensis.
**References.** (74), (82), (85), (86), (87), (88), (89), (90), (91)
**Age References. (74), (88), (91)**
**Env References. (74), (82)**
**Taxa References. (82),** (85), (86), (87), (89), (90), (91)

**23. Baillie-Hamilton Island**
**Stratigraphy/Location.** Cape Phillips Formation/ Baillie-Hamilton Island, Nunavut, Canada.
**Age.** Llandovery (Telychian).
**Environment.** BA5
**Taxa.**
Thelodonti: Loganellia scotica.
**References.** (12), (92)
**Age References. (92)**
**Env References. (12)**
**Taxa References. (92)**

**24. Balturino District**
**Stratigraphy/Location.** Balturino Formation/ Balturino District, Russia.
**Age.** Llandovery (Telychian), Moyerocanian, Khaastyrian, and Agidyan Regional Stages.
**Environment.** BA1–2
**Taxa.**
Thelodonti: Loganellia asiatica, Loganellia indet., Loganellia sibirica, Talimaalepis kadvoiensis, Talimaalepis rimae.
Acanthodii: Tchunacanthus sp., Tchunacanthus sp. indet.
Chondrichthyes: Mongolepis rozmani, Teslepis jucunda, Udalepis sp.
**References.** (53), (57), (58), (60), (61), (63)
**Age References. (58), (60), (63)**
**Env References. (58), (60), (63)**
**Taxa References. (53), (57), (61)**

**25. Chongqing1**
**Stratigraphy/Location.** Upper Xiushan and Huixingshao Formations/ Chongqing, Hunan, China.
**Age.** Llandovery (Telychian).
**Environment.** BA0–3
**Taxa.**
Galeaspida: Eugaleaspis xiushanensis, Miaojiaaspis dichotomus, Yongdongaspis littoralis.
Chondrichthyes: Chenolepis asketa, Rongolepis cosmetica, Shenacanthus vermiformi, Shiqianolepis hollandi, Sinacanthus wuchangenis, Xinjiangichthys pluridentatus, Xinjiangichthys sp.
Placodermi: Xiushanosteus mirabilis.
**References.** (26), (74), (93), (94), (95), (96), (97), (98), (99)
**Age References. (74), (94),**
**Env References. (97), (99)**
**Taxa References. (26), (74), (93), (95), (96), (98)**

**26. Coolin Lough**
**Stratigraphy/Location.** Killbride Formation/ Coolin Lough, Ireland.
**Age.** Llandovery (Telychian).
**Environment.** BA0–1
**Taxa.**
Thelodonti: Loganellia aldridgei, Loganellia asiatica, Loganellia scotica, Loganellia sibirica, Loganellia sp. cf. L. scotica.
**References.** (65), (66), (67), (76)
**Age References. (76)**
**Env References. (65)**
**Taxa References. (65), (66), (67)**

**27. Devon Island**
**Stratigraphy/Location.** Cape Phillips Formation/ Cape Phillips, Devon Island, Nunavut, Canada.
**Age.** Llandovery (Telychian).
**Environment.** BA5
**Taxa.**
Thelodonti: Loganellia aldridgei, Loganellia asiatica, Loganellia scotica, Loganellia sibirica, Loganellia sp. cf. L. scotica.
**References.** (12), (65), (66), (67), (76), (77)
**Age References. (76), (77)**
**Env References. (12), (65)**
**Taxa References. (65), (66), (67)**

**28. Elegest and Kadvoi**
**Stratigraphy/Location.** Angatchi (Angachi), Akchalym, Dashtygoi Horizon, Elegest Formation/ Elegest and Kadvoi Localities, Tuva, Russia.
**Age.** Llandovery (Aeronian–Telychian).
**Environment.** BA1–3?
**Taxa.**
Thelodonti: Loganellia aldridgei, Loganellia asiatica, Loganellia scotica, Loganellia sibirica, Loganellia sp. cf. L. cuneata, Loganellia sp. cf. L. scotica, Loganellia tuvaensis, Talimaalepis kadvoiensis, Talimaalepis rimae.
Acanthodii: Tchunacanthus obruchevi.
Chondrichthyes: Elegestolepis sp., Udalepis sp.
**References.** (12), (61), (62), (63), (65), (66), (67), (76), (77)
**Age References. (61), (63), (76), (77)**
**Env References. (12), (61), (63)**
**Taxa References. (62), (65), (66), (67)**

**29. Fentou Formation**
**Stratigraphy/Location.** Fentou Formation/ Hubei, Anhui, and Jiangsu Provinces, China.
**Age.** Llandovery (Telychian).
**Environment.** BA1–2
**Taxa.**
Galeaspida: Geraspis rara, Hanyangaspis chaohuensis, Hanyangaspis pani, Hanyangaspis sp., Latirostraspis caohuensis.
Chondrichthyes: Neosinacanshus planispinatus, Neosinacanshus sp.2, Sinacanthus sp., Sinacanthus triangulates.
**References.** (12), (74), (96), (100), (101)
**Age References. (74)**
**Env References. (12)**
**Taxa References. (74), (96), (100), (101)**

**30. Gotland1**
**Stratigraphy/Location.** Lower Visby Formation/ Gotland, Sweden.
**Age.** Llandovery (Telychian).
**Environment.** BA4–5
**Taxa.**
Thelodonti: Loganellia aldridgei, Loganellia asiatica, Loganellia scotica, Loganellia sibirica, Loganellia sp. cf. L. scotica.
**References.** (65), (66), (67), (102), (103)
**Age References. (102), (103)**
**Env References. (102)**
**Taxa References. (65), (66), (67), (103)**

**31. Gullett Quarry**
**Stratigraphy/Location.** Wych Formation/ Gullett Quarry, Worcestershire, UK.
**Age.** Llandovery (Telychian).
**Environment.** BA4
**Taxa.**
Thelodonti: Loganellia aldridgei, Loganellia asiatica, Loganellia scotica, Loganellia sibirica, Loganellia sp. cf. L. scotica.
**References.** (12), (65), (66), (67)
**Age References. (65), (66)**
**Env References. (12)**
**Taxa References. (65), (66), (67)**

**32. Hall Land1**
**Stratigraphy/Location.** Lafayette Bugt Formation/ Hall Land, Greenland.
**Age.** Llandovery (Telychian).
**Environment.** BA1–
**Taxa.**
Thelodonti: Loganellia aldridgei, Loganellia asiatica, Loganellia scotica, Loganellia sibirica, Loganellia sp. cf. L. scotica.
**References.** (12), (65), (66), (67), (104), (105), (106)
**Age References. (65), (66), (104)**
**Env References. (12), (106)**
**Taxa References. (65), (66), (67), (104), (105)**

**)33. Lesmahagow**
**Stratigraphy/Location.** Kip Burn and Patrick Burn Formation, Priesthill Group/ Lesmahagow, Lanarkshire, Midland Valley, Scotland.
**Age.** Llandovery (Telychian).
**Environment.** BA2–
**Taxa.**
Thelodonti: Loganellia aldridgei, Loganellia asiatica, Loganellia scotica, Loganellia sibirica, Loganellia sp. cf. L. scotica.
**References.** (65), (66), (76), (104), (105), (107), (108)
**Age References. (65), (66), (76), (104), (105), (107), (108)**
**Env References. (12)**
**Taxa References. (65), (66), (104), (105), (107), (108)**

**34. North Esk Inlier**
**Stratigraphy/Location.** Reservoir Formation (Gutterford Burn Formation)/ North Esk Inlier, Pentland Hills, Midland Valley, Scotland.
**Age.** Llandovery (Telychian).
**Environment.** BA0
**Taxa.**
Thelodonti: Loganellia aldridgei, Loganellia asiatica, Loganellia scotica, Loganellia sibirica, Loganellia sp. cf. L. scotica.
**References.** (12), (65), (66), (67), (76), (79)
**Age References. (65), (66), (76), (79)**
**Env References. (12)**
**Taxa References. (65), (66), (67), (79)**

**35. Shaanxi**
**Stratigraphy/Location.** Wuxiahe (Wujiahe) Formation/ Shaanxi, China.
**Age.** Llandovery (Telychian).
**Environment.** BA3–5?
**Taxa.**
Galeaspida: Hanyangaspis guodingshanensis.
**References.** (74), (109), (110), (111)
**Age References. (74), (109), (111)**
**Env References. (74), (110)**
**Taxa References. (74)**

**36. Shanks Castle**
**Stratigraphy/Location.** Ceratiocaris Bed, Kip Burn Formation/ Shanks Castle, Logan Water, Lanarkshire, Midland Valley, Scotland.
**Age.** Llandovery (Telychian).
**Environment.** BA0–1
**Taxa.**
Anaspida: Birkenia sp., Jamoytius kerwoodi.
**References.** (12), (107), (112), (113)
**Age References. (107), (112), (113)**
**Env References. (12)**
**Taxa References. (107), (113)**

**37. Shipan Reservoir**
**Stratigraphy/Location.** Xikeng Formation/ Shipan Reservoir, Jiangxi, China.
**Age.** Llandovery.
**Environment.** BA1–2
**Taxa.**
Galeaspida: Rumporostralis shipanensis.
**References.** (74), (114), (115)
**Age References. (74)**
**Env References. (74), (115)**
**Taxa References. (74), (114)**

**38. Tielikewatie Village**
**Stratigraphy/Location.** Tataertag Formation/ Tielikewatie Village, Kalpin County, Xinjiang, China.
**Age.** Llandovery (Telychian).
**Environment.** BA1–2
**Taxa.**
Galeaspida: Microphymaspis pani, Platylomaspis serratus, Platycarpaspis tianshanensis, Xiyuaspis zhangi.
**References.** (86), (88), (89), (90), (116), (117), (118)
**Age References. (86), (88), (89), (90), (117), (118)**
**Env References. (88)**
**Taxa References. (86), (89), (116)**

**39. Tururkhansk District**
**Stratigraphy/Location.** Omnutakh Formation/ Tururkhansk District, Russia.
**Age.** Llandovery (Telychian, Agidyan Regional Stage).
**Environment.** BA0–1
**Taxa.**
Thelodonti: Talimaalepis rimae.
**References.** (58), (61)
**Age References. (61)**
**Env References. (58)**
**Taxa References. (61)**

**40. Washington Land**
**Stratigraphy/Location.** Kap Tyson Outcrop, Hauge Bjerge and Lafayette Bugt Formations/ Peary Land Group, Washington Land, Greenland.
**Age.** Llandovery (Telychian).
**Environment.** BA1–
**Taxa.**
Anaspida: Birkenia?.
**References.** (106), (113)
**Age References. (113)**
**Env References. (106)**
**Taxa References. (113)**

**41. Wuhan**
**Stratigraphy/Location.** Guodingshan Formation/ Wuhan, Hubei, China.
**Age.** Llandovery.
**Environment.** BA1–2, 3?
**Taxa.**
Galeaspida: Geraspis rara, Hanyangaspis guodingshanensis.
Chondrichthyes: Neosincanthus planispinatus, Sinacanthus triangulates, Sinacanthus wuchangenis.
**References.** (67), (83), (119), (120), (121)
**Age References. (67), (83), (119), (120),**
**Env References. (121)**
**Taxa References. (67), (119)**

**42. Wulff Land**
**Stratigraphy/Location.** Thors Fjord Member, Wulff Land Formation/ Wulff Land, Greenland.
**Age.** Llandovery (Telychian).
**Environment.** BA1–
**Taxa.**
Thelodonti: Loganellia scotica.
**References.** (105), (106)
**Age References. (105)**
**Env References. (106)**
**Taxa References. (105)**

**43. Zhangjiajie**
**Stratigraphy/Location.** Rongxi Formation/ Zhangjiajie, Hunan, China.
**Age.** Llandovery (Telychian).
**Environment.** BA1–2
**Taxa.**
Galeaspida: Dayongaspis hunanensis, Konoceraspis grondoculus, Konoceraspis sp.
Chondrichthyes: Neosinacanshus sp., Neosinacanshus sp.2, Sinacanthus sp.
**References.** (74), (96), (120), (122)
**Age References. (74)**
**Env References. (74)**
**Taxa References. (96), (120), (122)**

**44. Xiushui**
**Stratigraphy/Location.** Xikeng Formation/ Xiushui, Jiangxi, China.
**Age.** Llandovery–Wenlock (Telychian–Sheinwoodian).
**Environment.** BA1–2
**Taxa.**
Galeaspida: Rumporostralis xikengensis, Sinogaleaspis shankouensis, Sinogaleaspis zhejiangensis, Xiushuiaspis jiangxiensis, Xiushuiaspis ganbeiensis.
**References.** (74), (81), (114), (123), (124)
**Age References. (74), (124)**
**Env References. (74)**
**Taxa References. (74), (81), (114), (123)**

**45. Avalanche Lake1**
**Stratigraphy/Location.** Road River and Whittaker Formations, Delorme Group/ Avalanche Lake, Mackenzie Mountains, Canada.
**Age.** Llandovery (Telychian).
**Environment.** BA2–4?
**Taxa.**
Anaspida: Birkenia elegans.
Thelodonti: Archipelepis bifurcata, Archipelepis turbinata, Lanarkia sp. cf. horrida, Pezopallichthys ritchiei.
Heterostraci: Ariaspis sp., Athenaegis chattertoni, Ptomaspis sp.
**References.** (12), (113), (125), (126)
**Age References. (113), (125), (126)**
**Env References. (12)**
**Taxa References. (113), (125)**

**46. Changxing**
**Stratigraphy/Location.** Lower Maoshan Formation/ Changxing, Zhejiang, China.
**Age.** Llandovery (Telychian–Sheinwoodian).
**Environment.** BA0–2
**Taxa.**
Galeaspida: Anjiaspis reticularis, Changxingaspis gui, Hanyangaspis sp., Meishanaspis lehmani, Shuyu zhejiangensis, Sinogaleaspis zhejiangensis.
Chondrichthyes: Sinacanthus sp.
**References.** (74), (85), (96), (127), (128)
**Age References. (74), (127)**
**Env References. (74)**
**Taxa References. (74), (85), (96), (127), (128)**

**47. Ellesmere Island**
**Stratigraphy/Location.** Whittaker and Road River formations/ North Shore of Baumann Fiord section, Ellesmere Island, Queen Elizabeth Islands, Nunavut, Canada.
**Age.** Uppermost Telychian to Early Sheinwoodian.
**Environment.** BA3
**Taxa.**
Thelodonti: Archipelepis turbinata.
**References.** (12), (126)
**Age References. (126)**
**Env References. (12)**
**Taxa References. (126)**

**48. Mount Tchagartay**
**Stratigraphy/Location.** Khutsynbulak Beds, Chargat Formation/ Mount Tchagartay, Mongolia.
**Age.** Llandovery (Telychian–Sheinwoodian, Salhit Regional Stage).
**Environment.** BA2–3?
**Taxa.**
Thelodonti: Talimaalepis kadvoiensis, Talimaalepis rimae.
Chondrichthyes: Mongolepis rozmanae, Sodolepis lucens, Teslepis jucunda.
**References.** (61), (129), (130), (131)
**Age References. (61)**
**Env References. (129)**
**Taxa References. (61), (130), (131)**

**C. Wenlock Sites**

**1. Chargat Locality**
**Stratigraphy/Location.** Chargat Formation/ Chargat locality, Lake Khar-Us, Northwestern Mongolia.
**Age.** Wenlock (Early Sheinwoodian), Salhit Regional Stage.
**Environment.** BA2–3?.
**Taxa.**
Thelodonti: Loganellia asiatica, Loganellia sibrica, Loganellia tuvaensis, Talimaalepis kadvoiensis, Talimaalepis rimae.
Chondrichthyes: Deltalepis parva, Deltalepis magna.
**References.** (19), (61)
**Age References. (19), (61)**
**Env References. (61)**
**Taxa References. (19), (61)**

**2. Cornwallis Island**
**Stratigraphy/Location.** Allen Bay Formation/ Cornwallis Island, Canada.
**Age.** Wenlock (Lower Sheinwoodian).
**Environment.** BA3.
**Taxa.**
Heterostraci: Tolypelepis sp.
**References.** (12), (132)
**Age References. (12)**
**Env References. (12)**
**Taxa References. (132)**

**3. Thorsteinsson Quarry**
**Stratigraphy/Location.** Cape Phillips Formation/ Thorsteinsson Quarry, Cape Phillips, Cornwallis Island, Nunavut, Canada.
**Age.** Wenlock (Lower Sheinwoodian).
**Environment.** BA5.
**Taxa.**
Anaspida: Birkenia elegans, Ha ha.
Thelodonti: Archipelepis bifurcata, Archipelepis turbinate, Eestilepis prominens, Erepsilepis margaritifera, Illoganellia colossa, Loganellia avonia, Loganellia colossea, Loganellia einari, Loganellia grossi, Loganellia prolata, Loganellia sulcata, Phillipsilepis cornuta, Phillipsilepis pusilla, Phillipsilepis crassa, Shielia gibba, Shielia parca, Shielia multispinata, Shielia taiti, Thelodus inauditus, Thelodus calvus.
Chondrichthyes: Frigorilepis caldwelli, Wellingtonella gagnieri.
**References.** (12), (66), (76), (113), (126), (133), (134), (135), (136), (137), (138)
**Age References. (66), (76), (113), (126), (134)**, (135), (136)
**Env References. (12), (133)**
**Taxa References. (76), (113), (135), (136), (137), (138)**

**4. Asker2**
**Stratigraphy/Location.** Malmoya Formation/ Gjettum Station, Asker, Oslo, Norway.
**Age.** Wenlock (Sheinwoodian).
**Environment.** BA0–3?.
**Taxa.**
Thelodonti: Loganellia einari.
**References.** (70), (139)
**Age References. (70)
Env References. (70)
Taxa References. (139)**

**5. Brinkmarsh Quarry**
**Stratigraphy/Location.** Brinkmarsh Beds/ Brinkmarsh Quarry, Whitefield, UK.
**Age.** Wenlock (Sheinwoodian).
**Environment.** BA3.
**Taxa.**
Thelodonti: Loganellia avonia.
**References.** (12), (79)
**Age References. (79)
Env References. (12)
Taxa References. (79)**

**6. Clew Bay**
**Stratigraphy/Location.** Bunnamohaun Siltstone/ Clew Bay, County Mayo, Ireland.
**Age.** Wenlock (Sheinwoodian).
**Environment.** BA0.
**Taxa.**
Anaspida: Birkenia elegans.
**References.** (113), (140), (141)
**Age References. (113)
Env References. (140), (141)
Taxa References.** (113)

**7. Elegest and Kadvoi2**
**Stratigraphy/Location.** Pichishui horizon, Baytal Formation/ Elegest and Kadvoi Localities, Central Tuva, Russia.
**Age.** Wenlock (Sheinwoodian).
**Environment.** BA1.
**Taxa.**
Thelodonti: Helenolepis multicostata, Helenolepis obruchevi, Helenolepis sp., Loganellia tuvaensis, Talimaalepis kadvoiensis.
Osteostraci: indet.
Acanthodii: Tchunacanthus sp.
Chondrichthyes: Elegestolepis grossi, Udalepis sp.
**References.** (63), (142), (143)
**Age References. (63)
Env References. (63)
Taxa References. (63), (142), (143)**

**8. Tortworth Inlier**
**Stratigraphy/Location.** Brinkmarsh Beds/ Tortworth Inlier, Gloucestershire, UK.
**Age.** Wenlock (Sheinwoodian).
**Environment.** BA3–4.
**Taxa.**
Thelodonti: Loganellia avonia, Loganellia colossea, Loganellia einari, Loganellia prolata, Loganellia sulcata, Logania sp. cf. cuneata, Logania cruciformis, Logania ludlowiensis, Logania scotica, Thelodus sp. cf. traquairi.
**References.** (12), (133), (144)
**Age References. (133)
Env References. (12)
Taxa References. (144)

9. Avalanche Lake2**
**Stratigraphy/Location.** Road River Formation, Delorme Group/ Avalanche Lake Sections, Mackenzie Mountains, Northwestern Canada.
**Age.** Wenlock (Sheinwoodian and Homerian).
**Environment.** BA3.
**Taxa.**
Heterostraci: Archegonaspis sp., Ariaspis sp., Vernonaspis sp., gen. indet.
Anaspida: Birkenia elegans?, Birkenia sp.
Thelodonti: Archipelepis bifurcata, Archipelepis turbinate, Overia adraini, Paralogania consimilis, Paralogania martinssoni, Pezopallichthys ritchiei.
**References.** (12), (126), (133), (145), (146), (147), (148)
**Age References. (133), (145), (146), (147)
Env References. (12), (126)
Taxa References. (133), (145), (146), (147), (148)**

**10. Balturino District2**
**Stratigraphy/Location.** Barmo Formations/ Balturino, Siberia, Russia.
**Age.** Wenlock (Sheinwoodian–Homerian), Khakomian Regional Stage.
**Environment.** BA0–1.
**Taxa.**
Thelodonti: Loganellia sp. indet., Talimaalepis rimae.
**References.** (58), (149)
**Age References. (58), (149)
Env References. (149)
Taxa References. (58)**

**11. Cape Lucie Marie**
**Stratigraphy/Location.** Kap Morton Formation/ Cape Lucie Marie, Washington Land, Northern Greenland.
**Age.** Wenlock (Sheinwoodian and Homerian).
**Environment.** BA3–.
**Taxa.**
Thelodonti: Loganellia avonia, Loganellia colossea, Loganellia einari, Loganellia exilis, Loganellia grossi, Loganellia prolata, Loganellia sulcata.
**References.** (76), (106), (133), (150), (151)
**Age References. (76), (106), (133), (150), (151)
Env References. (151)
Taxa References. (151)**

**12. East Baltic**
**Stratigraphy/Location.** Maasi, Tagavere, Viita, and Kuusnomme Beds/ Estonia and Latvia, East Baltic.
**Age.** Wenlock (Sheinwoodian–Homerian).
**Environment.** BA1–.
**Taxa.**
Anaspida: Birkenia elegans, Birkenia sp., Schidiosteus mustelensis.
Heterostraci: Cyathaspis integer.
Osteostraci: Tremataspis sp., Witaaspis shrenkii.
Thelodonti: Loganellia avonia, Loganellia colossea, Loganellia einari, Loganellia exilis, Loganellia grossi, Loganellia sulcata, Phlebolepis elegans, Paralogania consimilis, Paralogania martinssoni, Thelodus calvus, Thelodus carinatus, Thelodus laevis, Oeselia mosaica.
**References.** (12), (53), (62), (66), (67), (76), (78), (79), (113), (146), (152), (153), (154), (155), (156), (157), (158), (159)
**Age References. (53), (67), (76), (113), (146), (155)
Env References. (12), (157)
Taxa References. (53), (63), (66), (67), (78), (79), (113), (146), (152), (153), (154), (156), (158), (159)**

**13. Gotland2**
**Stratigraphy/Location.** Halla, Mulde, Klinteberg, and Slite Formation/ Gotland, Sweden.
**Age.** Wenlock (Sheinwoodian and Homerian).
**Environment.** BA3–5.
**Taxa.**
Anaspida: Birkeniida sp. 1, Birkeniida sp. 2, Birkeniida sp. 3, Pterygolepis nitida, Rhyncholepis parvula, Rhyncholepis butriangula, Rytidolepis quenstedti, Schidiosteus mustelensis.
Osteostraci: Oeselaspis sp., Tremataspis sp., gen. indet.
Thelodonti: Loganellia avonia, Loganellia colossea, Loganellia einari, Loganellia exilis, Loganellia grossi, Loganellia martinssoni, Loganellia prolata, Loganellia sulcate, Logania martinssoni, Paralogania consimilis, Paralogania ex gr. martinssoni, Paralogania martinssoni, Paralogania sp. cf. ludlowiensis, Thelodus calvus, Thelodus carinatus, Thelodus laevis, Thelodus parvidens, Thelodus schmidti, Thelodus visvaldi.
**References.** (12), (53), (76), (102), (103), (113), (133), (146), (150), (160), (161)
**Age References. (102), (103)
Env References. (12), (76)
Taxa References. (53), (103), (113), (133), (146), (150), (160), (161)**

**14. Hagshaw Hills**
**Stratigraphy/Location.** Fish Bed and Smithy Burn Formations, Glenbuck Group/ Hagshaw Hills, Old Red Sandstone, Midland Valley, Scotland.
**Age.** Wenlock (Homerian and Sheinwoodian).
**Environment.** BA0.
**Taxa.**
Anaspida: Birkenia elegans.
Thelodonti: Lanarkia horrida, Lanarkia lanceolata, Lanarkia sp., Lanarkia spinulosa, Shielia taiti.
**References.** (76), (107)
**Age References. (76)
Env References. (76)
Taxa References. (76), (107)**

**15. Jaagarahu**
**Stratigraphy/Location.** Jaagarahu Formation, Vilsandi, Sa and Massi Beds/ Saaremaa Island, Estonia.
**Age.** Wenlock (Sheinwoodian–Homerian).
**Environment.** BA1–.
**Taxa.**
Anaspida: Pterygolepis nitida, Rhyncholepis parvula.
Osteostraci: Ateleaspis sp. cf. tessellate.
Thelodonti: Loganellia grossi, Loganellia einari.
**References.** (113), (159), (162), (163), (164)
**Age References. (113), (159), (162)
Env References. (159)
Taxa References. (113), (159), (163), (164)**

**16. Kochumdek District2**
**Stratigraphy/Location.** Usas Formation/ Kochumdek District, Pretunguska, East Siberia, Russia.
**Age.** Wenlock, Khakomian Regional Stage.
**Environment.** BA1–.
**Taxa.**
Thelodonti: Talimaalepis rimae.
**References.** (58), (61), (149)
**Age References. (58), (61), (149)
Env References. (149)
Taxa References. (61)**

**17. Lesmahagow2**
**Stratigraphy/Location.** Slot Burn and Birkenhead Burn Formations/ Lesmahagow, Lanarkshire, Midland Valley, Scotland.
**Age.** Wenlock (Sheinwoodian and Homerian).
**Environment.** BA0–1.
**Taxa.**
Anaspida: Birkenia elegans.
Thelodonti: Lanarkia horrida, Lanarkia lanceolata, Lanarkia sp., Lanarkia spinulosa.
Osteostraci: Ateleaspis tesselata.
**References.** (76), (107), (113), (165), (166), (167)
**Age References. (76), (113), (166)
Env References. (76)
Taxa References. (107), (113), (165), (167)**

**18. Lyne Water**
**Stratigraphy/Location.** Fish Bed and Henshaw Formations/ Lyne Water, Pentland Hills, Midland Valley, Scotland.
**Age.** Wenlock (Sheinwoodian and Homerian).
**Environment.** BA0–1.
**Taxa.**
Anaspida: Birkenia elegans, Lasanius problematicus.
Thelodonti: Shielia gibba, Shielia multispinata, Shielia parca, Shielia taiti.
**References.** (12), (76), (113), (133), (135), (165)
**Age References. (76), (113)
Env References. (12), (76)
Taxa References. (76), (113), (133), (135), (165)**

**19. Matusevich River**
**Stratigraphy/Location.** Samojlovich Formation/ Matusevich River, October Revolution Island, Severnaya Zemlya Archipelago, Russia.
**Age.** Wenlock (Sheinwoodian & Homerian).
**Environment.** BA1–.
**Taxa.**
Anaspida: Birkenia elegans, Birkenia robusta, Birkenia sp.
Thelodonti: Thelodus calvus, Thelodus carinatus, Thelodus laevis, Loganellia avonia, Loganellia colossea, Loganellia einari, Loganellia exilis, Loganellia grossi, Loganellia prolata, Loganellia sulcata.
**References.** (53), (66), (75), (133), (146)
**Age References. (53), (75), (146)
Env References. (75)
Taxa References. (53), (66), (133), (146)**

**20. Pioneer Island2**
**Stratigraphy/Location.** Upper Samojlovich Formation/ Pioneer Island, Severnaya Zemlya Archipelago, Russia.
**Age.** Wenlock (Sheinwoodian and Homerian).
**Environment.** BA1–.
**Taxa.**
Anaspida: Rhyncholepis parvula, Rytidolepis quenstedti.
Thelodonti: Paralogania consimilis, Paralogania martinssoni, Thelodus calvus, Thelodus carinatus, Thelodus laevis, Thelodus visvaldi.
**References.** (53), (66), (75), (133), (146)
**Age References. (53), (75), (146)
Env References. (75)
Taxa References. (53), (66), (133), (146)**

**21. Spokoinaya-Ushakov River1**
**Stratigraphy/Location.** Samojlovich Formation/ Spokoinaya River and Ushalov River, October Revolution Island, Severnaya Zemlya Archipelago, Russia.
**Age.** Wenlock (Sheinwoodian & Homerian).
**Environment.** BA1–3?.
**Taxa.**
Anaspida: Birkenia elegans?, Pterygolepis nitida, Schidiosteus mustelensis.
Thelodonti: Shielia multispinata, Paralogania consimilis.
**References.** (53), (66), (75), (113), (133), (135), (146)
**Age References. (53), (75), (113), (146)
Env References. (75)
Taxa References. (53), (66), (113), (133), (135), (146)**

**22. Strojnaya River3**
**Stratigraphy/Location.** Samojlovich Formation/ Strojnaya River, October Revolution Island, Severnaya Zemlya Archipelago, Russia.
**Age.** Wenlock (Sheinwoodian & Homerian).
**Environment.** BA1–.
**Taxa.**
Thelodonti: Thelodus calvus, Thelodus carinatus, Thelodus inauditus, Thelodus laevis, Thelodus visvaldi, Loganellia avonia, Loganellia colossea, Loganellia einari, Loganellia exilis, Loganellia grossi, Loganellia prolata, Loganellia sulcata, Shielia gibba, Shielia parca, Shielia taiti.
**References.** (53), (66), (75), (133), (146)
**Age References. (66), (75), (146)
Env References. (75)
Taxa References. (53), (133), (146)**

**23. Baillie-Hamilton Island2**
**Stratigraphy/Location.** Cape Phillips Formation/ Baillie-Hamilton Island, Nunavut, Canada.
**Age.** Wenlock (Sheinwoodian and Homerian).
**Environment.** Deep sub-tidal marine BA5.
**Taxa.**
Anaspida: Birkeniida gen. et sp. indet. 1, Birkeniida gen. et sp. indet. 2, Birkeniida gen. et sp. indet. 3, Birkenia elegans, Birkenia elegans?, Birkenia sp., Ha et sp.
Heterostraci: Eriptychiida gen. et sp. indet., Traquairaspidida gen. et sp. indet.
Thelodonti: Archipelepis bifurcata, Archipelepis turbinate, Loganellia sp. 1, Loganellia sp. 2, Loganellia einari, Loganellia grossi, Paralogania consimilis, Paralogania martinssoni, Phillipsilepis cornuta, Phillipsilepis crassa, Phillipsilepis pusilla.
Chondrichthyes: Kannathalepis milleri.
**References.** (12), (76), (92), (113), (126), (133), (138), (146)
**Age References. (76), (113), (126)
Env References. (12)
Taxa References. (92), (113), (133), (138), (146)**

**24. Grodno Region**
**Stratigraphy/Location.** Ostrovets Formation/ Grodno Region, Belarus.
**Age.** Wenlock (Homerian).
**Environment.** BA1–.
**Taxa.**
Thelodonti: Loganellia einari, Loganellia grossi, Thelodus sp.
**References.** (168)
**Age References. (168)
Env References. (168)
Taxa References. (168)**

**25. Qinling Mountains**
**Stratigraphy/Location.** Miaogou Formation/ Gansu, Zoige and Tewo County, Sichuan Province, West Qinling Mountains, China.
**Age.** Wenlock (Homerian).
**Environment.** BA1–.
**Taxa.**
Acanthodii: Nostolepis tewonensis.
**References.** (74), (94), (169), (170), (171)
**Age References. (74), (94), (170), (171)
Env References. (170)
Taxa References. (74), (169)**

**26. Ringerike1**
**Stratigraphy/Location.** Ranberget Member, Steinsfjorden, Sundvollen Formation/ Norway.
**Age.** Wenlock (Late Homerian).
**Environment.** BA0–3?.
**Taxa.**
Anaspida: Birkenia elegans?, Pterygolepis nitida, Rhyncholepis parvula, Pharyngolepis oblonga.
Thelodonti: Loganellia avonia, Loganellia einari, Loganellia exilis, Loganellia grossi, Loganellia colossea, Loganellia sulcate, Loganellia prolata.
Osteostraci: cf. Tyriaspis whitei, gen. indet.
**References.** (70), (113), (172)
**Age References. (70), (113), (172)
Env References. (70)
Taxa References. (113)**

**27. Derenjal Mountains**
**Stratigraphy/Location.** Niur Formation/ Derenjal Mountains, Iran.
**Age.** Late Wenlock–Ludlow (Homerian–Gorstian).
**Environment.** BA1–2.
**Taxa.**
Thelodonti: Niurolepis susanae.
**References.** (173)
**Age References. (173)
Env References. (173)
Taxa References. (173)**

**28. Longwood Shale**
**Stratigraphy/Location.** Longwood Shale Formation/ New Jersey, USA.
**Age.** Wenlock–Ludlow (Homerian–Gorstian).
**Environment.** BA1.
**Taxa.**
Heterostraci: Vernonaspis sp.
**References.** (12), (132), (174)
**Age References. (174)
Env References. (12)
Taxa References. (132), (174)**

**29. Rootsikula**
**Stratigraphy/Location.** Rootsikula Formation, Iide, Viita, Kuusnomme, Vesiku, Anikaitse and Soeginina Beds/ Saaremaa Island, Estonia.
**Age.** Wenlock–Ludlow (Homerian–Gorstian).
**Environment.** BA1–.
**Taxa.**
Anaspida: Birkenia robusta, Pterygolepis nitida, Rhyncholepis parvula, Rhyncholepis butriangula, Rytidolepis quenstedti, Vesikulepis funiforma.
Osteostraci: Meelaidaspis gennadii, Aestiaspis viitaensis, Tremataspis perforata, Tremataspis rohoni, Tremataspis schmidti, Tremataspis milleri, Tremataspis schrenkii, Tremataspis sp., Witaaspis schrenkii.
Thelodonti: Paralogania martinssoni.
**References.** (113), (159), (162), (163), (164)
**Age References. (159), (162),
Env References. (159)
Taxa References. (113), (163), (164)**

**30. Shawangunk Formation**
**Stratigraphy/Location.** Medina Formation, Shawangunk Formation/ New York, USA.
**Age.** Wenlock–Ludlow (Homerian–Gorstian).
**Environment.** BA1.
**Taxa.**
Heterostraci: Eoarchegonaspis wardelli, Vernonaspis sp., Vernonaspis vaningeni.
**References.** (12), (67), (76), (132), (175), (176), (177), (178)
**Age References. (67), (76), (178)
Env References. (12), (76)
Taxa References. (67), (132), (175), (176), (177)**

**D. Ludlow Sites

1. Appalachian Mountains**
**Stratigraphy/Location.** Ludlow-Bloomsburg Formation/ Appalachian Mountains, US.
**Age.** Ludlow (Gorstian).
**Environment.** BA0
**Taxa.** Anaspida: Septentrionia dissimilis.
**References.** (66), (178)
**Age References. (178)**
**Env References. (178)**
**Taxa References. (66)

2. Bachu-Kalpin2**
**Stratigraphy/Location.** Upper Keziertag Formation/ Kalpin Bachu, Tarim Basin, China.
**Age.** Ludlow (Gorstian).
**Environment.** BA0.
**Taxa.** Galeaspida: Jiaoyu indet., Microphymaspis pani, Xiyuaspis zhangi. Placodermi: Arthorodira indet.
**References.** (86), (87), (89), (90)
**Age References. (87), (89), (90)**
**Env References. (86)**
**Taxa References. (87), (89)**

**3. Chance’s Pitch**
**Stratigraphy/Location.** Chance’s Pitch, Malvern Hills, Herefordshire, UK.
**Age.** Ludlow (Gorstian).
**Environment.** BA3
**Taxa.** Thelodonti: Phlebolepis elegans, Phlebolepis ornata.
**References.** (12), (66), (67), (79), (153), (156), (182)
**Age References. (66), (67), (79), (182)**
**Env References. (12)**
**Taxa References. (66), (67), (79), (153), (156), (182)**

**4. Cwar Glas**
**Stratigraphy/Location.** Black Cock Beds/ Cwar Glas, Dyfed, Wales, UK.
**Age.** Ludlow (Gorstian).
**Environment.** BA1
**Taxa.** Heterostraci: Archegonaspis ludensis.
**References.** (132), (182)
**Age References. (132), (182)**
**Env References. (182)**
**Taxa References. (132), (182)**

**5. Mortimer Forest**
**Stratigraphy/Location.** Middle Elton Formation, Goggin Road/ Mortimer Forest, Shropshire, UK.
**Age.** Ludlow (Gorstian).
**Environment.** BA4–5.
**Taxa.** Thelodonti: Paralogania consimilis, Paralogania kaarmisensis, Paralogania ludlowiensis, Paralogania menneri, Thelodus admirabilis, Thelodus calvus, Thelodus carinatus, Thelodus hoskinsi, Thelodus laevis, Thelodus marginatus, Thelodus matukhini, Thelodus parvidens, Thelodus schmidti, Thelodus sculptilis, Thelodus sinensis, Thelodus sp., Thelodus traquairi, Thelodus trilobatus, Thelodus visvaldi.
**References.** (12), (146), (153), (155), (182), (183), (184)
**Age References. (92), (155), (182)**
**Env References. (12)**
**Taxa References. (146), (153), (182), (184)**

**6. Pioneer Island 3**
**Stratigraphy/Location.** Ust-Spokojnaya Formation/ Pioneer Island, Severnaya Zemlya Archipelago, Russia.
**Age.** Ludlow (Gorstian).
**Environment.** BA2–4?
**Taxa.** Thelodonti: Paralogania consimilis, Paralogania kaarmisensis, Paralogania ludlowiensis, Paralogania menneri.
**References.** (53), (66), (75), (146), (155)
**Age References. (53), (75), (155)**
**Env References. (75)**
**Taxa References. (53), (66), (146)**

**7. Ringerike2**
**Stratigraphy/Location.** Ringerike Group, Sundvollen Formation/ Ringerike, Oslo, Norway.
**Age.** Ludlow (Gorstian).
**Environment.** BA0–1
**Taxa.** Anaspida: Pharyngolepis oblonga, Pterygolepis cf. P. nitida, Rhyncholepis parvula. Thelodonti: Loganellia einari, Loganellia sp.?, Paralogania martinssoni, Thelodus sp.
**References.** (70), (113), (139), (172)
**Age References. (70), (113), (172)**
**Env References. (70)**
**Taxa References. (113), (139)**

**8. Somerset Island2**
**Stratigraphy/Location.** Peel Sound Formation/ Somerset Island, Northwest Territories, Canada.
**Age.** Ludlow (Gorstian).
**Environment.** BA0–1
**Taxa.** Heterostraci: Alainaspis platyrhina, Rhachiaspis pteriga.
**References.** (12), (179), (180), (185)
**Age References. (179), (180)**
**Env References. (12)**
**Taxa References. (185)**

**9. Wita Quarry**
**Stratigraphy/Location.** Wita Quarry/ Oesel Island, Estonia.
**Age.** Ludlow (Gorstian).
**Environment.** ?
**Taxa.** Osteostraci: Rotsikiillaspis obrutchevi.
**References.** (186)
**Age References. (186)**
**Env References. ?**
**Taxa References. (186)**

**10. Arisaig1**
**Stratigraphy/Location.** Mcadam Brook and Moydart Formation/ Arisaig, Cape George, Nova Scotia, Canada.
**Age.** Ludlow (Gorstian and Ludfordian).
**Environment.** BA1
**Taxa.** Thelodonti: Thelodus admirabilis, Thelodus calvus, Thelodus carinatus, Thelodus hoskinsi, Thelodus laevis, Thelodus marginatus, Thelodus matukhini, Thelodus parvidens, Thelodus schmidti, Thelodus sculptilis, Thelodus sinensis, Thelodus sp., Thelodus traquairi, Thelodus trilobatus, Thelodus visvaldi.
**References.** (12), (67), (76), (146), (153), (155), (157), (183), (184), (187)
**Age References. (67), (76), (155), (157), (183), (184)**
**Env References. (12), (184)**
**Taxa References. (67), (146), (153), (184), (187)**

**11. Baillie-Hamilton Island3**
**Stratigraphy/Location.** Cape Phillips Formation/ Baillie-Hamilton Island, Nunavut, Canada.
**Age.** Ludlow (Ludfordian and Gorstian).
**Environment.** BA4–6.
**Taxa.** Anaspida: Birkeniidae gen. et sp. indet. Thelodonti: Loganellia asiatica, Loganellia sp. cf. grossi, Loganellia cuneata, Loganellia incompta, Loganellia sp4., Nunavutia fasciata.
**References.** (12), (78), (92)
**Age References. (92)**
**Env References. (12)**
**Taxa References. (78), (92)**

**12. Derenjal Mountains2**
**Stratigraphy/Location.** Niur Formation/ Derenjal Mountains, Iran.
**Age.** Ludlow (Gorstian–Ludfordian).
**Environment.** BA1–2.
**Taxa.** Thelodonti: Loganellia sp. cf. L. grossi. Acanthodii: indet.
**References.** (173)
**Age References. (173)**
**Env References. (173)**
**Taxa References. (173)**

**13. East Baltic2**
**Stratigraphy/Location.** Vesiku, Soeginina, Himmiste, Tahula, Uduvere, Aigu, and Kudjape Beds, Kuressaare, Ohesaare, and Paadla Formations/ Estonia and Latvia, East Baltic.
**Age.** Ludlow (Gorstian–Ludfordian), Kuressaare, Paadla, Kaugatuma Regional Stages.
**Environment.** BA2–3
**Taxa.** Anaspida: Pharyngolepis sp., Tahulalepis elongituberculata, Schidiosteus mustelensis, Silmalepis erinacea. Heterostraci: Archegonaspis sp., Archegonaspis sp. B, Cyathaspis integer. Osteostraci: Tremataspis sp. Thelodonti: Loganellia cuneata, Loganellia sp. cf. L. grossi, Loganellia incompta, Oeselia mosaica, Paralogania consimilis, Paralogania kaarmisensis, Paralogania ludlowiensis, Paralogania menneri, Paralogania sp. cf. P. ludlowiensis, Phlebolepis elegans, Phlebolepis ornata, Thelodus sculptilis, Thelodus parvidens, Thelodus schmidti, Thelodus admirabilis, Thelodus calvus, Thelodus carinatus, Thelodus hoskinsi, Thelodus laevis, Thelodus marginatus, Thelodus matukhini, Thelodus sinensis, Thelodus sp., Thelodus traquairi, Thelodus trilobatus, Thelodus visvaldi.
**References.** (12), (78), (113), (152), (153), (154), (159)
**Age References. (78), (113)**
**Env References. (12)**
**Taxa References. (78), (113), (152), (153), (154), (159)**

**14. Elegest and Kadvoi 3**
**Stratigraphy/Location.** Taungateli Horizon, Baytal Formation, Khondergei Formation/ Elegest and Kadvoi Rivers, Tuva, Russia.
**Age.** Ludlow (Gorstian–Ludfordian).
**Environment.** BA1–3?
**Taxa.** Thelodonti: Helenolepis navicularis, Loganellia sp. cf. L. grossi, Loganellia cuneata, Loganellia incompta, Loganellia cf. L. tuvaensis, Loganellia unispinata.
**References.** (12), (63), (78), (104)
**Age References. (63), (78)**
**Env References. (12)**
**Taxa References. (63), (78), (104)**

**15. Gotland3**
**Stratigraphy/Location.** Hemse Group, Eke, Burgsvik, Hamra, and Sundre Formation/ Gotland, Sweden.
**Age.** Ludlow (Ludfordian and Gorstian).
**Environment.** BA2–3.
**Taxa.** Anaspida: Liivilepis curvata, Pharyngolepis sp., Schidiosteus mustelensis, Septentrionia dissimilis, Septentrionia mucronata, Tahulalepis elongituberculata. Heterostraci: Archegonaspis lindstromi, Cyathaspis integer. Osteostraci: gen. indet., Tahulaspis sp. Thelodonti: Loganellia cuneata, Loganellia sp. cf. L. grossi, Loganellia incompta, Paralogania consimilis, Paralogania kaarmisensis, Paralogania ludlowiensis, Paralogania menneri, Paralogania perensae, Phlebolepis elegans, Phlebolepis ornata, Thelodus parvidens, Thelodus carinatus, Thelodus admirabilis, Thelodus calvus, Thelodus hoskinsi, Thelodus laevis, Thelodus marginatus, Thelodus matukhini, Thelodus schmidti, Thelodus sculptilis, Thelodus sinensis, Thelodus sp., Thelodus traquairi, Thelodus trilobatus, Thelodus visvaldi. Osteichthyes: Andreolepis hedei, Andreolepis petri.
**References.** (12), (53), (62), (66), (67), (76), (78), (80), (103), (104), (113), (137), (146), (153), (154), (155), (158), (160), (183), (184), (188), (189), (190), (191), (192), (193)
**Age References. (53), (62), (66), (67), (76), (78), (80), (103), (104), (113), (137), (146), (153), (154), (155), (158), (183), (184), (189), (190), (193)**
**Env References. (12)**
**Taxa References. (53), (62), (66), (67), (78), (103), (113), (137), (146), (153), (154), (158), (160), (183), (184), (188), (189), (190), (191), (192), (193)**

**16. Kenwood**
**Stratigraphy/Location.** Vernon Formation/ Kenwood, New York, USA.
**Age.** Ludlow (Gorstian–Ludfordian).
**Environment.** BA1
**Taxa.** Heterostraci: Archegonaspis drummondi, Vernonaspis sp., Vernonaspis allenae, Vernonaspis leonardi.
**References.** (12), (76), (174), (176)
**Age References. (76), (174), (176)**
**Env References. (12), (76)**
**Taxa References. (174), (176)**

**17. Leopold Formation1**
**Stratigraphy/Location.** Cape Clarence Member, Leopold Formation/ Nunavut, Canada.
**Age.** Ludlow (Gorstian–Ludfordian).
**Environment.** BA1?–2
**Taxa.** Heterostraci: Archegonaspis sp.
**References.** (76)
**Age References. (76)**
**Env References. (76)**
**Taxa References. (76)**

**18. Longwood Shale**
**Stratigraphy/Location.** Longwood Shale Formation/ New Jersey, USA.
**Age.** Ludlow (Gorstian–Ludfordian).
**Environment.** BA0–1
**Taxa.** Heterostraci: Americaspis claypolei.
**References.** (12), (132)
**Age References. (12), (132)**
**Env References. (12)**
**Taxa References. (132)**

**19. Matusevich River2**
**Stratigraphy/Location.** Ust'-Spokojnaya Formation/ Matusevich River, Pioneer Island, Severnaya Zemlya Archipelago, Russia.
**Age.** Ludlow (Gorstian–Ludfordian).
**Environment.** BA2–4?
**Taxa.** Anaspida: Schidiosteus mustelensis, Spokoinolepis alternans. Thelodonti: Paralogania kaarmisensis, Paralogania menneri, Paralogania martinssoni, Phlebolepis elegans, Thelodus sp., Thelodus visvaldi, Valiukia flabellata, Valiukia sp.
**References.** (53), (75), (113), (146)
**Age References. (75)**
**Env References. (75)**
**Taxa References. (53), (113), (146)**

**20. Monroe County**
**Stratigraphy/Location.** Bloomsburg Formation/ Mt. Union, Monroe, Pennsylvania, US.
**Age.** Ludlow (Ludfordian and Gorstian).
**Environment.** BA0
**Taxa.** Heterostraci: Vernonaspis bryanti, Vernonaspis vaningeni, Vernonaspis sp., Americaspis americana. Thelodonti: Thelodus admirabilis, Thelodus calvus, Thelodus carinatus, Thelodus hoskinsi, Thelodus laevis, Thelodus marginatus, Thelodus matukhini, Thelodus parvidens, Thelodus schmidti, Thelodus sculptilis, Thelodus sinensis, Thelodus sp., Thelodus traquairi, Thelodus trilobatus, Thelodus visvaldi.
**References.** (12), (62), (67), (76), (132), (153), (155), (157), (174), (183), (194)
**Age References. (62), (67), (76), (155), (157), (183)**
**Env References. (12)**
**Taxa References. (62), (67), (132), (153), (157), (174), (194)**

**21. Novaya Zemlya**
**Stratigraphy/Location.** Samojlovich and Ust' Spokoinaya Formation/ Novaya Zemlya Archipelago, Russia.
**Age.** Ludlow (Ludfordian and Gorstian).
**Environment.** ?
**Taxa.** Thelodonti: Thelodus admirabilis, Thelodus calvus, Thelodus carinatus, Thelodus hoskinsi, Thelodus laevis, Thelodus marginatus, Thelodus matukhini, Thelodus parvidens, Thelodus schmidti, Thelodus sculptilis, Thelodus sinensis, Thelodus sp., Thelodus traquairi, Thelodus trilobatus, Thelodus visvaldi.
**References.** (12), (76), (155), (157), (183)
**Age References. (12), (76), (155), (157), (183)**
**Env References. ?**
**Taxa References. (76), (157), (183)**

**22. October Revolution Island**
**Stratigraphy/Location.** Ust-Spokojnaya Formation/ October Revolution Island, Severnaya Zemlya Archipelago, Russia.
**Age.** Ludlow (Ludfordian and Gorstian).
**Environment.** BA2–4?
**Taxa.** Anaspida: Birkeniida sp., Pterygolepis nitida, Rytidolepis quenstedti. Thelodonti: Loganellia sp. cf. L. grossi, Loganellia incompta, Thelodus admirabilis, Thelodus calvus, Thelodus carinatus, Thelodus hoskinsi, Thelodus laevis, Thelodus marginatus, Thelodus matukhini, Thelodus parvidens, Thelodus schmidti, Thelodus sculptilis, Thelodus sinensis, Thelodus sp., Thelodus traquairi, Thelodus trilobatus, Thelodus visvaldi, Paralogania kaarmisensis, Paralogania ludlowiensis, Paralogania martinssoni, Paralogania consimilis, Paralogania ex gr. martinssoni, Paralogania sp. cf. P. ludlowiensis, Valiukia sp. 1.
**References.** (12), (53), (62), (66), (67), (75), (78), (146), (153), (155), (157), (158), (183), (184)
**Age References. (53), (62), (67), (75), (155), (157), (158), (183), (184)**
**Env References. (12), (184)**
**Taxa References. (53), (62), (66), (67), (78), (146), (153), (158), (183), (184)**

**23. Oesel Island**
**Stratigraphy/Location.** Paadla Formation/ Oesel Island, Estonia.
**Age.** Ludlow (Gorstian–Ludfordian).
**Environment.** BA3
**Taxa.** Anaspida: Rhyncholepis (Saarolepis) oeselensis. Osteostraci: Cephalaspis oeselensis, Dartmuthia gemmifera, Oeselaspis pustulata, Saaremaaspis mickwitzi, Thyestes verrucosus, Tremataspis mamillata, Tremataspis milleri, Tremataspis panderi, Tremataspis patteni, Tremataspis rohoni, Tremataspis scalaris, Tremataspis schmidti, Witaaspis patteni, Witaaspis schrenkii. Thelodonti: Coelolepis luhai. Heterostraci: Tolypelepis undulata.
**References.** (12), (175), (195), (196), (197), (198), (199)
**Age References. (198), (199)**
**Env References. (12), (184)**
**Taxa References. (175), (195), (196), (197), (198)**

**24. Paadla**
**Stratigraphy/Location.** Paadla Formation, Sauvere, Himmiste and Uduvere Beds/ Saaremaa Island, Estonia.
**Age.** Ludlow (Gorstian–Ludfordian).
**Environment.** BA3
**Taxa.** Anaspida: Rytidolepis quenstedti, Saarolepis (Rhyncholepis) oeselensis, Schidiosteus mustelensis, Silmalepis erinacea, Vesikulepis funiforma. Osteostraci: Dartmuthia gemmifera, Dartmuthia procera, Oeselaspis pustulata, Procephalaspis aff. oeselensis, Procephalaspis oeselensis, Saaremaaspis mickwitzi, Tahulaspis praevia, Thyestes verrucosus, Tremataspis mamillata, Tremataspis milleri, Tremataspis obruchevi, Tremataspis patteni, Tremataspis perforata, Tremataspis rohoni, Witaaspis schrenkii. Thelodonti: Longodus acicularis, Paralogania consimilis, Paralogania kaarmisensis, Paralogania martinssoni, Paralogania menneri, Phlebolepis elegans, Phlebolepis ornata, Thelodus sculptilis. Osteichthyes: Andreolepis herdei.
**References.** (12), (113), (137), (159), (162), (163), (164)
**Age References. (159), (162)**
**Env References. (12)**
**Taxa References. (137), (159), (163), (164)**

**25. Spokoinaya-Ushakov River2**
**Stratigraphy/Location.** Ust-Spokojnaya Formation, Spokoinaya River and Ushalov River/ October Revolution Island, Severnaya Zemlya Archipelago, Russia.
**Age.** Ludlow (Gorstian–Ludfordian).
**Environment.** BA3?
**Taxa.** Anaspida: Birkenia elegans?, Birkenia sp.?, Rhyncholepis parvula, Schidiosteus mustelensis, Spokoinolepis alternans, Vesikulepis funiforma. Heterostraci: indet.
**References.** (75), (113)
**Age References. (75), (113)**
**Env References. (75)**
**Taxa References. (113)**

**26. Sunnyhill Quarry**
**Stratigraphy/Location.** Elton, Bringewood, and Leintwardine Formation/ Sunnyhill Quarry, Sunnydingle Cottage, Shropshire, UK.
**Age.** Ludlow (Gorstian–Ludfordian).
**Environment.** BA3
**Taxa.** Thelodonti: Paralogania ludlowiensis, Paralogania martinssoni, Paralogania kaarmisensis, Phlebolepis ornata, Phlebolepis elegans, Thelodus sp.
**References.** (12), (53), (66), (67), (79), (146), (153), (155), (156), (188), (191)
**Age References. (53), (67)**, (155)
**Env References. (12),**
**Taxa References. (53), (66), (67), (79), (146), (153), (156), (188), (191)**

**27. Yukon1**
**Stratigraphy/Location.** Road River Formation/ Yukon Territory, Canada.
**Age.** Ludlow (Gorstian–Ludfordian).
**Environment.** BA2–3?
**Taxa.** Heterostraci: Ariaspis ornata, Dikenaspis yukonensis, Homalaspis borealis, Ptomaspis canadensis, Vernonaspis epitegosa, Vernonaspis major, Vernonaspis sekwiae.
**References.** (12), (132), (174)
**Age References. (12)**
**Env References. (12)**
**Taxa References. (132), (174)**

**28. Baojing**
**Stratigraphy/Location.** Xiaoxi Formation/ Baojing, Northwestern Hunan Province, China.
**Age.** Ludlow (Ludfordian).
**Environment.** BA1
**Taxa.** Galeaspida: Dunyu xiushanensis, Dunyu cf. xiushanensis, Eugaleaspis cf. xiushanensis.
**References.** (93), (200), (201)
**Age References. (93), (200)**
**Env References. (200), (201)**
**Taxa References. (200)**

**29. Church Hill**
**Stratigraphy/Location.** Lower Leintwardine Formation/ Church Hill, Herefordshire, UK.
**Age.** Ludlow (Ludfordian).
**Environment.** BA2–3
**Taxa.** Heterostraci: Archegonaspis ludensis.
**References.** (12), (182)
**Age References. (12), (182)**
**Env References. (12)**
**Taxa References. (182)**

**30. Irian Jaya**
**Stratigraphy/Location.** Kemum Formation?/ Lorentz River, Irian Jaya, Papua, Western New Guinea.
**Age.** Ludlow (Ludfordian).
**Environment.** BA1
**Taxa.** Thelodonti: Thelodus sp. cf. T. trilobatus, Turinia sp., indet. Acanthodii: Gomphonchus sp. cf. G. sandelensis, Nostolepis sp. cf. N. striata, fam. gen. et sp. indet., indet. Chondrichthyes: gen. et sp. indet.
**References.** (202), (203), (204), (205), (206), (207)
**Age References. (202), (204), (206), (207)**
**Env References. (12), (204), (205)**
**Taxa References. (202), (204)**

**31. Lixian**
**Stratigraphy/Location.** Xiaoxi Formation/ Shanmen, Reservoir, Lixian County, Hunan Province, China.
**Age.** Ludlow (Ludfordian).
**Environment.** BA1
**Taxa.** Placodermi: Shimenolepis graniferus.
**References.** (93), (200), (201), (208), (209)
**Age References. (93), (200)**
**Env References. (12), (200)**
**Taxa References. (200), (208), (209)**

**32. Much Wenlock**
**Stratigraphy/Location.** Whitcliffe (Overton) Formation and Downton Castle Formation/ Ludlow Much Wenlock District, Shropshire, UK.
**Age.** Ludlow–Pridoli (Ludfordian–Pridoli).
**Environment.** BA1
**Taxa.** Thelodonti: Thelodus admirabilis, Thelodus calvus, Thelodus carinatus, Thelodus hoskinsi, Thelodus marginatus, Thelodus matukhini, Thelodus schmidti, Thelodus sculptilis, Thelodus sinensis, Thelodus sp., Thelodus traquairi, Thelodus trilobatus, Thelodus parvidens, Nethertonodus prodigialis.
**References.** (62), (66), (155), (157), (158), (183), (210)
**Age References. (62), (66), (183), (210)**
**Env References. (12), (157),**
**Taxa References. (62), (66), (155), (158), (183)**

**33. Mount Sekwi**
**Stratigraphy/Location.** Delorme Group/ Mount Sekwi, Keele River, Canada.
**Age.** Ludlow (Ludfordian).
**Environment.** BA3
**Taxa.** Heterostraci: Vernonaspis sekwiae.
**References.** (12), (132)
**Age References. (132)**
**Env References. (12)**
**Taxa References. (132)**

**34. Onibury**
**Stratigraphy/Location.** Leintwardine Formation/ Onibury, Shropshire, UK.
**Age.** Ludlow (Ludfordian).
**Environment.** BA3
**Taxa.** Thelodonti: Paralogania kaarmisensis, Paralogania consimilis, Paralogania ludlowiensis, Paralogania menneri, Paralogania perensae.
**References.** (12), (66), (76), (155), (191)
**Age References. (66), (76), (155), (191)**
**Env References. (12),**
**Taxa References. (66), (76)**

**35. Chongqing2**
**Stratigraphy/Location.** Xiaoxi Formation/ Chongqing, Hunan, China.
**Age.** Ludlow (Late Ludfordian).
**Environment.** BA1
**Taxa.** Placodermi?: Bianchengichthys micros.
**References.** (93), (200), (201)
**Age References. (93), (200)
Env References. (200), (201)**
**Taxa References. (93), (200)**

**36. East Yunnan**
**Stratigraphy/Location.** Kuanti, Miaogao and Yulungssu Formations/ Qujing, East Yunnan, China.
**Age.** Ludlow (Ludfordian–Pridoli).
**Environment.** BA1–3
**Taxa.** Galeaspida: Dunyu longiforus, Polybranchiaspis sp. Thelodonti: Thelodus sinensis, Thelodus admirabilis, Thelodus calvus, Thelodus carinatus, Thelodus hoskinsi, Thelodus laevis, Thelodus marginatus, Thelodus matukhini, Thelodus parvidens, Thelodus schmidti, Thelodus sculptilis, Thelodus sp., Thelodus traquairi, Thelodus trilobatus, Thelodus visvaldi. Acanthodii: Gomphonchus sp., Hanilepis wangi, Nostolepis sinica, Nostolepis sp., Nostolepis striata, Poracanthodes qujingensis. Osteichthyes: Guiyu oneiros, Kawalepis comptus, Ligulalepis yunnanensis, Megamastax amblyodus, Naxilepis gracilis, Sparalepis tingi. Placodermi: Entelognathus primordialis, Silurolepis platydorsalis, Qilinyu rostrata.
**References.** (122), (128), (146), (153), (155), (157), (170), (183), (211), (212), (213), (214), (215), (216), (217), (218)
**Age References. (155), (157), (170), (183), (211), (214)
Env References. (128), (157)**
**Taxa References. (122), (128), (146), (153), (183), (212), (213), (214), (215), (216), (217), (218)**

**37. Kureessaare-Kaugatuma**
**Stratigraphy/Location.** Kuressaare and Kaugatuma Formation, Tahula, Kudjape and Aigu Beds/ Saaremaa Island, Estonia.
**Age.** Ludlow–Pridoli (Late Ludfordian–).
**Environment.** BA3
**Taxa.** Anaspida: Liivilepis curvata, Tahulalepis elongituberculata, Trimpleylepis concatenate. Osteostraci: Dartmuthia procera, Ohesaareaspis ponticulata, Tahulaspis aff. ordinata, Tahulaspis cf. praevia, Tahulaspis ordinata. Thelodonti: Longodus acicularis, Nethertonodus laadjalaensis, Paralogania consimilis, Paralogania kaarmisensis, Paralogania ludlowiensis, Paralogania menneri, Paralogania perensae, Thelodus admirabilis, Thelodus sculptilis. Acanthodii: Nostolepis gracilis.
**References.** (12), (80), (113), (159), (163), (164), (219), (220)
**Age References. (80), (113)
Env References. (12)**
**Taxa References. (113), (159), (163), (164), (219), (220)**

**38. Netherton**
**Stratigraphy/Location.** Whitcliffe Formation and Downton Castle Sandstone Formation/ Netherton, UK.
**Age.** Late Ludlow to Early Pridoli.
**Environment.** BA0–2?
**Taxa.** Thelodonti: Nethertonodus laadjalaensis, Nethertonodus prodigialis, Paralogania borealis, Paralogania cf. P. borealis, Paralogania foliala, Paralogania kachanovi, Paralogania consimilis, Paralogania kaarmisensis, Paralogania ludlowiensis, Paralogania menneri, Paralogania tarranti, Paralogania wilsoni, Thelodus parvidens.
**References.** (12), (66), (76), (182)
**Age References. (182)
Env References. (12), (76)**
**Taxa References. (66), (182)**

**39. Rzepin**
**Stratigraphy/Location.** Upper Winnica Formation/ Rzepin Section, Holy Cross Mountains, Poland.
**Age.** Late Ludlow (Ludfordian–Pridoli).
**Environment.** BA1
**Taxa.** Anaspida: indet. cf. Liivilepis. Thelodonti: Thelodus parvidens, Thelodus trilobatus, Paralogania ludlowiensis. Osteostraci: Tahulaspis cf. ordinata. Acanthodii: indet. cf. Gomphonchus, Nostolepis gracilis, Radioporacanthodes biblicus, Radioporacanthodes sp.
**References.** (221)
**Age References. (221)
Env References. (221)
Taxa References. (221)**

**40. Skane**
**Stratigraphy/Location.** Klinta and Oved Sandstone Formation, Oved Ramsasa Group/ Katina, Helvetesgraben, Rinnebacks bro, Ramsasa, Skane, South Sweden.
**Age.** Ludlow–Pridoli (Ludfordian–Pridoli).
**Environment.** BA2
**Taxa.** Anaspida: Liivilepis curvata, Ramsaalepis porosa, Tahulalepis elongituberculata. Thelodonti: Goniporous alatus, Loganellia asiatica, Loganellia cruciformis, Loganellia cuneata, Loganellia incompta, Loganellia sp., Loganellia sp. cf. L. grossi, Loganellia tuvaensis, Loganellia unispinata, Paralogania consimilis, Paralogania kaarmisensis, Paralogania ludlowiensis, Paralogania martinssoni, Paralogania menneri, Paralogania perensae, Thelodus admirabilis, Thelodus bicostatus, Thelodus calvus, Thelodus costatus, Thelodus hoskinsi, Thelodus laevis, Thelodus marginatus, Thelodus matukhini, Thelodus parvidens, Thelodus schmidti, Thelodus sculptilis, Thelodus sinensis, Thelodus sp., Thelodus traquairi, Thelodus trilobatus, Thelodus visvaldi, Trimerolepis tricava. Acanthodii: Nostolepis sp. Osteichthyes: Andreolepis sp.
**References.** (12), (53), (62), (66), (67), (76), (78), (80), (113), (146), (153), (155), (157), (158), (183), (191), (222)
**Age References. (66), (67), (76), (80), (113), (155), (183), (191), (222)
Env References. (12), (157)
Taxa References. (53), (62), (66), (67), (78), (113), (146), (153), (158), (183), (222)**

**41. Strojnaya River4**
**Stratigraphy/Location.** Ust'-Spokojnaya Formation and Krasnaya Bukhta/ Strojnaya River, October Revolution Island, Severnaya Zemlya Archipelago, Russia.
**Age.** Ludlow–Pridoli (Ludfordian–Pridoli).
**Environment.** BA2–4?
**Taxa.** Thelodonti: Paralogania ludlowiensis, Paralogania kaarmisensis, Paralogania menneri, Paralogania perensae, Paralogania borealis, Paralogania cf. borealis, Paralogania foliala, Paralogania kachanovi, Paralogania kummerowi, Paralogania tarranti, Paralogania wilsoni.
**References.** (53), (66), (75), (155), (191)
**Age References. (75), (155), (191)
Env References. (75)
Taxa References. (53), (66)**

**42. Tabuska Beds**
**Stratigraphy/Location.** Tabuska Beds/ Tabuska River, Central Ural Mountains, Russia.
**Age.** Ludlow–Pridoli (Ludfordian–Pridoli), Upper Paadla Regional Stage.
**Environment.** BA3?
**Taxa.** Anaspida: Liivilepis curvata, Schidiosteus mustelensis, Septentrionia dissimilis?, Septentrionia mucronata. Heterostraci: Archegonaspis sp., Cyathaspis alexanderi, Tolypelepis? Thelodonti: Thelodus parvidens, Thelodus sculptilis. Acanthodii: Nostolepis striata, Poracanthodes porosus. Osteichthyes: Andreolepis petri.
**References.** (56), (66), (113), (158)
**Age References. (56), (113)
Env References. (56)
Taxa References. (56), (66), (113), (158)**

**43. Tite’s Point**
**Stratigraphy/Location.** Whitcliffe Formation and Ludlow Bone Bed/ River Severn, Tite’s Point, Gloucestershire, UK.
**Age.** Ludlow–Pridoli (Ludfordian–Pridoli).
**Environment.** BA2?
**Taxa.** Heterostraci: Cyathaspis banksi, Cyathaspis sp. Thelodonti: Thelodus bicostatus, Thelodus parvidens, Thelodus pugniformis, Thelodus trilobatus. Acanthodii: Onchus sp., indet.
**References.** (66), (76), (182)
**Age References. (66), (76), (182)
Env References. (76)
Taxa References. (66), (182)**

**44. Winnica**
**Stratigraphy/Location.** Upper Winnica Formation/ Winnica section, Holy Cross Mountains, Poland.
**Age.** Late Ludlow (Ludfordian–Pridoli).
**Environment.** BA1
**Taxa.** Anaspida: indet. cf. Liivilepis. Thelodonti: Thelodus parvidens, Thelodus trilobatus. Acanthodii: Radioporacanthodes biblicus, Radioporacanthodes sp.
**References.** (103)
**Age References. (103)
Env References. (103)
Taxa References. (103)**

**E. Pridoli**

**1. Arisaig2**
**Stratigraphy/Location.** Stonehouse Formation/ Arisaig, Cape George, Nova Scotia, Canada.
**Age.** Pridoli.
**Environment.** BA1.
**Taxa.** Thelodonti: Thelodus cf. parvidens. Acanthodii: Machaeraporus stonehousensis.
**References.** (12), (184), (223)
**Age References. (184)
Env References. (12)
Taxa References. (184), (223)**

**2. Baillie-Hamilton Island4**
**Stratigraphy/Location.** Cape Phillips Formation/ Baillie-Hamilton Island, Nunavut, Canada.
**Age.** Pridoli.
**Environment.** BA5.
**Taxa.** Thelodonti: Trimerolepis lithuanica, Trimerolepis timanica, Trimerolepis triangulus, Trimerolepis tricava.
**References.** (12), (92), (153)
**Age References. (92)
Env References. (12)
Taxa References. (153)**

**3. Bertie Formation**
**Stratigraphy/Location.** Bertie Formation/ Konservat-Lagerstatte, southern Ontario, Canada.
**Age.** Pridoli.
**Environment.** BA1.
**Taxa.** Acanthodii: Nerepisacanthus denisoni.
**References.** (224)
**Age References. (224)
Env References. (224)
Taxa References. (224)**

**4. East Baltic3**
**Stratigraphy/Location.** Himmiste, Tahula, Uduvere, Aigu, and Kudjape Beds, Oesel, Jura, and Tilze Formations/ Estonia and Latvia, East Baltic.
**Age.** Pridoli.
**Environment.** BA3.
**Taxa.** Heterostraci: Oniscolepis dentata, Strosipherus indentatus, Tolypelepis undulata, Tylodus deltoides. Thelodonti: Goniporous alatus, Katoporodus tricavus, Loganellia asiatica, Loganellia cf. tuvaensis, Loganellia cuneata, Loganellia incompta, Loganellia tuvaensis, Loganellia unispinata, Logania kummerowi, Paralogania borealis, Paralogania cf. borealis, Paralogania foliala, Paralogania kachanovi, Paralogania kummerowi, Paralogania tarranti, Paralogania wilsoni, Thelodus admirabilis, Thelodus bicostatus, Thelodus carinatus, Thelodus costatus, Thelodus parvidens, Thelodus pugniformis, Thelodus sp., Thelodus traquairi, Thelodus trilobatus, Trimerolepis lithuanica, Trimerolepis triangulus, Trimerolepis tricava. Acanthodii: Onchus wheathillensis, Nostolepis alta, Nostolepis gracilis, Nostolepis kozhymica, Nostolepis paravolborthi, Nostolepis striata, Nostolepis zinaidae.
**References.** (12), (53), (62), (66), (67), (76), (78), (79), (113), (146), (152), (153), (155), (156), (157), (158), (159), (162), (183), (225)
**Age References. (67), (76), (113), (155), (162), (183)
Env References. (12), (157)
Taxa References. (53), (62), (66), (67), (78), (79), (113), (146), (152), (153), (156), (158), (159), (183), (225)**

**5. Gotland4**
**Stratigraphy/Location.** Burgsvik Sandstone/ Gotland, Sweden.
**Age.** Pridoli.
**Environment.** BA2.
**Taxa.** Thelodonti: Thelodus bicostatus, Thelodus carinatus, Thelodus costatus, Thelodus pugniformis, Thelodus sculptilis, Thelodus sp., Thelodus traquairi, Thelodus trilobatus. Osteichthyes: Andreolepis hedei, Andreolepis petri.
**References.** (12), (53), (62), (66), (67), (76), (78), (103), (113), (137), (146), (153), (155), (158), (161), (188), (189), (190), (191)
**Age References. (67), (76), (155), (189), (190), (191)
Env References. (12)
Taxa References. (53), (62), (66), (67), (78), (103), (113), (137), (146), (153), (158), (161), (188)**

**6. Holmestrand**
**Stratigraphy/Location.** Ringerike Group, Steinsfjorden, Upper Sundvollen Formation/ Holmestrand, Oslo, Norway.
**Age.** Pridoli.
**Environment.** BA0–1.
**Taxa.** Osteostraci: Hemicyclaspis kiaeri, Hemicyclaspis murchinsoni.
**References.** (12), (113), (172), (226)
**Age References. (113), (172), (226)
Env References. (12)
Taxa References. (113), (226)**

**7. Kenwood2**
**Stratigraphy/Location.** Vernon-Pittsford Formation/ Kenwood, New York, USA.
**Age.** Pridoli.
**Environment.** BA1.
**Taxa.** Heterostraci: Archaegonaspis sp., Vernonaspis sp.
**References.** (12), (67), (76)
**Age References. (67), (76)
Env References. (12)
Taxa References. (67)**

**8. Ledbury**
**Stratigraphy/Location.** Downton Castle Sandstone Formation and Ledbury Formation/ Ledbury, Herefordshire, UK.
**Age.** Pridoli.
**Environment.** BA0–1.
**Taxa.** Anaspida: Tahulalepis kingi. Heterostraci: Kallostrakon sp.. Osteostraci: Auchenaspis egertoni, Auchenaspis sp., Didymaspis grindrodi, Hemicyclaspis lightbodii, Hemicyclaspis murchisoni. Acanthodii: Onchus mirabilis, Onchus sp., Plectrodus mirabilis, Plectrodus sp..
**References.** (12), (113), (182)
**Age References. (113), (182)
Env References. (12)
Taxa References. (113), (182)**

**9. Leopold Formation2**
**Stratigraphy/Location.** Cape Clarence Member, Leopold Formation/ Nunavut, Canada.
**Age.** Pridoli.
**Environment.** BA1–2.
**Taxa.** Heterostraci: Corvaspis cf. arctica, Kallostrakon? sp. indet., Ptomaspis n.sp., Tolypelepis cf. leopoldensis, Tolypelepis leopoldensis.
**References.** (76), (227), (228)
**Age References. (76)
Env References. (76)
Taxa References. (227), (228)**

**10. Nerepis Hills**
**Stratigraphy/Location.** Clinton Member, Cunningham Creek Formation/ Nerepis Hills, New Brunswick, Canada.
**Age.** Pridoli.
**Environment.** BA1.
**Taxa.** Anaspida: Ctenopleuron nerepisense. Thelodonti: Thelodus macintoshi. Heterostraci: Cyathaspis acadica. Acanthodii: Nerepisacanthus denisoni.
**References.** (84), (184), (195), (229), (230), (231), (232)
**Age References. (232)
Env References. (232)
Taxa References. (84), (184), (195), (229), (231)**

**11. Pioneer Island 4**
**Stratigraphy/Location.** Krasnaya Bukhta Formation/ Pioneer Island, Severnaya Zemlya Archipelago, Russia.
**Age.** Pridoli.
**Environment.** BA2–3.
**Taxa.** Anaspida: Spokoinolepis alternans. Thelodonti: Loganellia asiatica, Loganellia cf. tuvaensis, Loganellia incompta, Loganellia tuvaensis, Loganellia unispinata, Paralogania borealis, Paralogania cf. borealis, Paralogania foliala, Paralogania kachanovi, Paralogania kummerowi, Paralogania tarranti, Paralogania wilsoni, Thelodus sp..
**References.** (12), (53), (66), (67), (75), (113), (146), (155)
**Age References. (67), (75), (113), (155)
Env References. (12), (75)
Taxa References. (53), (66), (67), (113), (146)**

**12. Somerset Island3**
**Stratigraphy/Location.** Somerset Formation/ Somerset Island, Northwest Territories, Canada.
**Age.** Pridoli.
**Environment.** BA1–2?3?.
**Taxa.** Heterostraci: Alainaspis platyrhina, Anchipteraspis crenulate, Archegonaspis schmidti, Ariaspis arctata, Boothiaspis alata, Boothiaspis ovata, Capitaspis giblingi, Corvaspis arctica, Pionaspis sp., Torpedaspis elongate. Osteostraci: Hemicyclaspis murchinsoni.
**References.** (12), (179), (180), (181), (233), (234), (235), (236)
**Age References. (179), (233), (235)
Env References. (12), (233)
Taxa References. (180), (181), (233), (234), (235), (236)**

**13. Timan2**
**Stratigraphy/Location.** Eptarma Formation and Ovinparma Formation 1-2 and Velikaya River Formation, Eptarma Group/ Northern Timan (Timan-Pechora Region), Russia.
**Age.** Pridoli.
**Environment.** BA0–1.
**Taxa.** Osteostraci: Timanaspis kossovoii. Heterostraci: Oniscolepis dentata. Thelodonti: Goniporous alatus, Paralogania consimilis, Paralogania kaarmisensis, Paralogania ludlowiensis, Paralogania menneri, Phlebolepis elegans, Trimerolepis timanica. Acanthodii: Gomphonchus abruptus, Gomphonchus minicostaus, Monospina erecta, Nostolepis alta, Nostolepis gracilis, Nostolepis kozhymica, Nostolepis paravolborthi, Nostolepis striata, Nostolepis zinaidae.
**References.** (12), (53), (56), (66), (67), (146), (155), (225), (237)
**Age References. (67), (155), (237)
Env References. (12)
Taxa References. (53), (56), (66), (67), (146), (225), (237)**

**14. Yukon2**
**Stratigraphy/Location.** Road River and Gossage Formation/ Yukon Territory, Canada.
**Age.** Pridoli.
**Environment.** BA1–3?.
**Taxa.** Heterostraci: Vernonaspis allenae, Vernonaspis bamberi, Vernonaspis bryanti, Vernonaspis epitegosa, Vernonaspis leonardi, Vernonaspis major.
**References.** (12), (132), (174), (238)
**Age References. (174)
Env References. (12), (238)
Taxa References. (132), (174)**

**15. Hall Land2**
**Stratigraphy/Location.** Chester Bjerg Formation/ Halls Grav locality and Monument, Hall Land, North Greenland.
**Age.** Pridoli–Lochkovian.
**Environment.** BA2–.
**Taxa.** Anaspida: Septentrionia seducta. Thelodonti: Loganellia sp. cf. L. tuvaensis, Nikolivia auriculata, Nikolivia sp., Paralogania denisoni, Paralogania foliala, Praetrilogania grabion, Talivalia elongata, Talivalia sp.. Heterostraci: Cyathaspidiformes indet., Oniscolepis sp., Poraspis sp.. Osteostraci: Indet.. Acanthodii: Climatiida indet., Gomphonchus cf. G. sandelensis, Nostolepis halli, Poracanthodes cf. P. punctatus, Indet.. Chondrichthyes: Indet..
**References.** (104), (113), (239)
**Age References. (104), (113)
Env References. (104)
Taxa References. (104), (113), (239)**

**Reference**

1. G. C. Young, Ordovician Microvertebrate Remains from the Amadeus Basin, Central Australia. J. Vert. Paleontol. 17, 1–25 (1997).

2. I. J. Sansom, P. W. Haines, P. Andreev, R. S. Nicoll, A New Pteraspidomorph From the Nibil Formation (Katian, Late Ordovician) of the Canning Basin, Western Australia. J. Vert. Paleontol. 33, 764–769 (2013).

3. N. S. Davies, I. J. Sansom, Ordovician vertebrate habitats: A Gondwanan perspective. Palaios 24, 717–722 (2009).

4. C. J. Edgoose, T. J. Munson, Geology and mineral resources of the Northern Territory: Warburton Basin. (2013).

5. B.-D. Erdtmann, Bernd Weber, H.-P. Schultze, Sven Egenhoff, A Possible Agnathan Plate from the Lower Arenig (Lower Ordovician) of South Bolivia. J. Vert. Paleontol. 20, 394–399 (2000).

6. I. J. Sansom, C. G. Miller, A. Heward, N. S. Davies, G. A. Booth, R. A. Fortey, F. Paris, Ordovician fish from the Arabian peninsula. Palaeontology 52, 337–342 (2009).

7. A. P. Heward, R. A. Fortey, C. G. Miller, G. A. Booth, New Middle Ordovician (Darriwilian) faunas from the Sultanate of Oman. Proc. Geol. Assoc. 134, 251–268 (2023).

8. A. P. Heward, G. A. Booth, R. A. Fortey, C. G. Miller, I. J. Sansom, Darriwilian shallow-marine deposits from the Sultanate of Oman, a poorly known portion of the Arabian margin of Gondwana. Geol. Mag. 155, 59–84 (2018).

9. A. Ritchie, J. Gilbert-Tomlinson, First Ordovician vertebrates from the southern hemisphere. Alcheringa Australas. J. Palaeontol. 1, 351–368 (1977).

10. I. J. Sansom, N. S. Davies, M. I. Coates, R. S. Nicoll, A. Ritchie, Chondrichthyan‐like scales from the Middle Ordovician of Australia. Palaeontology 55, 243–247 (2012).

11. N. S. Davies, I. J. Sansom, R. S. Nicoll, A. Ritchie, Ichnofacies of the Stairway Sandstone fish-fossil beds (Middle Ordovician, Northern Territory, Australia). Alcheringa: An Australasian Journal of Palaeontology 35, 553–569 (2011).

12. A. J. Boucot, C. Janis, Environment of the early Paleozoic vertebrates. Palaeogeogr. Palaeoclimatol. Palaeoecol. 41, 251–287 (1983).

13. I. J. Sansom, P. S. Andreev, “The Ordovician Enigma” in Evolution and Development of Fishes, Z. Johanson, C. Underwood, M. Richter, Eds. (Cambridge University Press, Cambridge, England, 2019), pp. 59–66.

14. J. Dzik, T. A. Moskalenko, Problematic scale-like fossils from the Ordovician of Siberia with possible affinities to vertebrates. Neues Jahrb. Geol. Palaontol. Abh. 279, 251–260 (2016).

15. G. C. Young, An Ordovician vertebrate from western New South Wales, with comments on Cambro-Ordovician vertebrate distribution patterns. Alcheringa: An Australasian Journal of Palaeontology 33, 79–89 (2009).

16. B. D. Webby, Lower Ordovician arthropod Trace Fossils from western New South Wales. Proceedings of the Linnean Society of New South Wales 107, 59––74 (1983).

17. H. P. Liu, R. M. McKay, J. N. Young, B. J. Witzke, K. J. McVey, X. Liu, A new Lagerstätte from the Middle Ordovician St. Peter Formation in northeast Iowa, USA. Geology 34, 969–972 (2006).

18. L. I. U. Huai-bao, Others, The Winneshiek Lagerstatte, Iowa, USA and Its Depositional Environments. Geological Journal of China Universities 15, 285 (2009).

19. P. S. Andreev, M. I. Coates, V. Karatajūtė-Talimaa, R. M. Shelton, P. R. Cooper, I. J. Sansom, Elegestolepis and its kin, the earliest monodontode chondrichthyans. J. Vert. Paleontol. 37, e1245664 (2017).

20. F. G. Aceñolaza, H. Miller, A. J. Toselli, Proterozoic–Early Paleozoic evolution in western South America—a discussion. Tectonophysics 354, 121–137 (2002).

21. G. Albanesi, J. L. Benedetto, P.-Y. Gagnier, Scabambaspis janvieri (vertebrata) y conodontes del Llandeiliano temprano en la formación La Cantera, Precordillera de San Juan, Argentina. Boletín de la Academia Nacional de Ciencias, 519–543 (1995).

22. T. Ørvig, Pycnaspis splendens, new genus, new species, a new ostracoderm from the upper Ordovician of north America. Proc. U. S. Natl. Mus. 108, 1–23 (1958).

23. R. J. Ross Jr, Ordovician Fossils From Wells in the . Williston Basin Eastern Montana. U.S. Geol. Survey, Bull. 1021-M, 439–510 (1957).

24. I. J. Sansom, M. P. Smith, Late Ordovician vertebrates from the Bighorn Mountains of Wyoming, USA. Palaeontology 48, 31–48 (2005).

25. M. D. Fahrenbach, F. V. Steece, J. F. Sawyer, K. A. McCormick, G. L. McGillivray, L. D. Schulz, J. A. Redden, South Dakota Stratigraphic Correlation Chart (2017). https://www.nrc.gov/docs/ml1224/ML12240A243.pdf.

26. Y.-A. Zhu, Q. Li, J. Lu, Y. Chen, J. Wang, Z. Gai, W. Zhao, G. Wei, Y. Yu, P. E. Ahlberg, M. Zhu, The oldest complete jawed vertebrates from the early Silurian of China. Nature 609, 954–958 (2022).

27. G. Houée, J. Bardin, D. Germain, P. Janvier, N. Goudemand, Developmental models shed light on the earliest dental tissues, using Astraspis as an example. Palaeontology 66 (2023).

28. S. Turner, A. Blieck, G. S. Nowlan, “30. Vertebrates (Agnathans and Gnathostomes)” in The Great Ordovician Biodiversification Event, B. D. Webby, F. Paris, M. L. Droser, I. G. Percival, Eds. (Columbia University Press, New York Chichester, West Sussex, 2004), pp. 327–335.

29. B. Cameron, S. Mangion, Depositional environments and revised stratigraphy along the Black River-Trenton boundary in New York and Ontario. Am. J. Sci. 277, 486–502 (1977).

30. K. A. Lehtola, Ordovician Vertebrates from Ontario. Museum of paleontology the unversity of Michigan 24, No. 4, 23–30 (1973).

31. I. Sansom, D. Elliott, A thelodont from the Ordovician of Canada. Journal of Vertebrate Paleontology 22, 867–870 (2003).

32. J. C. Lamsdell, P. A. Isotalo, D. M. Rudkin, M. J. Martin, A new species of the Ordovician horseshoe crab Lunataspis. Geol. Mag. 160, 167–171 (2023).

33. C. D. Walcott, Preliminary notes on the discovery of a vertebrate fauna in Silurian (Ordovician) strata. Geol. Soc. Am. Bull. 3, 153–172 (1892).

34. C. R. Eastman, Devonic Fishes of the New York Formation (New York State Education Dept, Albany, 1907).

35. Greg Graffin, A New Locality of Fossiliferous Harding Sandstone: Evidence for Freshwater Ordovician Vertebrates. J. Vert. Paleontol. 12, 1–10 (1992).

36. I. Sansom, M. M. Smith, M. P. Smith, Scales of thelodont and shark-like fishes from the Ordovician of Colorado. Nature 379, 628–630 (1996).

37. M. M. Smith, I. J. Sansom, Exoskeletal micro-remains of an Ordovician fish from the Harding Sandstone of Colorado. Palaeontology 40, 645–658 (1997).

38. I. J. Sansom, M. Paul Smith, M. M. Smith, P. Turner, Astraspis - The anatomy and histology of an Ordovician fish. Palaeontology 40, 625–643 (1997).

39. J. L. Allulee, The sequence stratigraphic and environmental context of primitive vertebrates: Harding sandstone, upper Ordovician, Colorado, USA. PALAIOS 20, 518–533 (2005).

40. A. Lemierre, D. Germain, A new mineralized tissue in the early vertebrate Astraspis. J. Anat. 235, 1105–1113 (2019).

41. I. J. Sansom, M. M. Smith, M. P. Smith, The Ordovician radiation of vertebrates. SYSTEMATICS ASSOCIATION SPECIAL VOLUME 61, 156–171 (2001).

42. M. P. Smith, P. C. J. Donoghue, I. J. Sansom, The spatial and temporal diversification of Early Palaeozoic vertebrates. Geol. Soc. Spec. Publ. 194, 69–83 (2002).

43. L. P. Alberstadt, Articulate brachiopods of the Viola Formation (Ordovician) in the Arbuckle Mountains, Oklahoma. Oklahoma. Oklahoma Geological Survey Bulletin 117, 1–90 (1973).

44. C. R. Ossian, M. A. Halseth, Discovery of Ordo- vician vertebrates in the Arbuckle Mountains of Oklahoma. Journal of Paleontology 50, 773–777 (1976).

45. T. W. Amsden, The Late Ordovician brachiopod genera Lepidocyclus and Hiscobeccus. Oklahoma Geological Survey Bulletin 132, 36–44 (1983).

46. G. Gao, S. I. Dworkin, L. S. Land, R. D. Elmore, Geochemistry of late Ordovician viola limestone, Oklahoma: Implications for marine carbonate mineralogy and isotopic compositions. J. Geol. 104, 359–367 (1996).

47. W. Sinclair, Occurrence of fish in the Ordovician of Canada: Bull. Bull. Geol. Soc. America, v 69 (1958).

48. O. S. Hersi, G. R. Dix, Blackriveran (lower Mohawkian, Upper Ordovician) lithostratigraphy, rhythmicity, and paleogeography: Ottawa Embayment, eastern. Can. J. Earth Sci. 36, 2033–2050 (1999).

49. P.-Y. Gagnier, Fosiles y facies de Bolivia. I. Vertebrados. Revista Técnica de YPFB 12, 371–379 (1992).

50. C. Emig C, Dignomia munsterii (Brachiopoda, Lingulata) from the Ordovician of Bolivia, with redescription of the genus. Geodiversitas, 227–237 (2006).

51. A. Pradel, I. J. Sansom, P.-Y. Gagnier, R. Ces- Pedes, P. Janvier, The tail of the Ordovi- cian fish Sacabambaspis. Biology Letters 3, 72–75 (2007).

52. L. S. Eliuk, Middle Ordovician fish-bearing beds from the st. Lawrence lowlands of Quebec. Can. J. Earth Sci. 10, 954–960 (1973).

53. T. Märss, V. Karatajūtė-Talimaa, Ordovician and Lower Silurian thelodonts from Severnaya Zemlya Archipelago (Russia). Geodiversitas 24, 381–404 (2002).

54. P. Mannik, O. K. Bogolepova, A. Pıldvere, A. P. Gubanov, New data on Ordovician–Silurian conodonts and stratigraphy from the Severnaya Zemlya archipelago. Russian Arctic. Geological Magazine, 497–519 (2009).

55. V. Karatajtite-Talimaa, Taxonomy of loganiid thelodonts. Modern Geology 21, 1–15 (1997).

56. T. Märss, Silurian cyathaspidid heterostracans of Northern Eurasia. Est. J. Earth Sci. 68, 113 (2019).

57. V. Karatajute-Talimaa, M. M. Smith, Tesakoviaspis concentrica: microskeletal remains of a new order of vertebrate from the Upper Ordovician and Lower Silurian of Siberia. Recent Advances in the Origin and Early Radiation of Vertebrates, 53–64 (2004).

58. Ž. Žigaitė, A. Blieck, Palaeobiogeographical significance of Early Silurian thelodonts from central Asia and southern Siberia. GFF 128, 203–206 (2006).

59. V. Karatajute-Talimaa, M. M. Smith, Early acanthodians from the lower Silurian of Asia. Trans. R. Soc. Edinb. Earth Sci. 93, 277–299 (2002).

60. A. S. Tesakov, Biostratigraphy of the Middle Pliocene-Eopleistocene of Eastern Europe. Transactions of the Geological Institute 554, 1–247 (2004).

61. Ž. Žigaitė, Endemic thelodonts (Vertebrata: Thelodonti) from the Lower Silurian of central Asia and southern Siberia. Earth Environ. Sci. Trans. R. Soc. Edinb. 104, 123–143 (2013).

62. J. M. J. Vergoossen, Late Silurian fish microfossils from Klinta and Rinnebäcks Bro (Scania, south Sweden), with remarks on the morphology of Nostolepis striata trunk scales. Scripta Geologica 123, 71–92 (2002).

63. N. V. Sennikov, O. A. Rodina, N. G. Izokh, O. T. Obut, New data on Silurian vertebrates of southern Siberia. Palaeoworld 24, 231–242 (2015).

64. C. Burrow, J. Valiukevičius, Diversity of tissues in acanthodians with Nostolepis-type histological structure. Acta Palaeontol. Pol. 50, 635–649 (2005).

65. R. Aldridge, S. Turner, G. L. Jones, D. Harper, Late Llandovery thelodonts and conodonts from the Kilbride Formation, Co Galway, western Ireland. GEOLOGICAL JOURNAL. VOL. 31, 359–367 (1996).

66. T. Märss, C. G. Miller, Thelodonts and distribution of associated conodonts from the Llandovery–lowermost Lochkovian of the Welsh Borderland. Palaeontology 47, 1211–1265 (2004).

67. J. J. Sepkoski Jr, Rates of speciation in the fossil record. Philos. Trans. R. Soc. Lond. B Biol. Sci. 353, 315–326 (1998).

68. S. Turner, G. S. Nowlan, Early Silurian microvertebrates of eastern Canada. ulletin du Muséumnational d’Histoire naturelle, Paris, 4e sér section C,n°17, 513–529 (1995).

69. S. Turner, J. J. Kuglitsch, D. L. Clark, Llandoverian Thelodont Scales from the Burnt Bluff Group of Wisconsin and Michigan. J. Paleontol. 73, 667–676 (1999).

70. O. Bremer, S. Turner, T. Märss, H. Blom, Silurian vertebrate remains from the Oslo Region, Norway, and their implications for regional biostratigraphy. Norw. J. Geol., doi: 10.17850/njg99-1-07 (2019).

71. O. Tinn, T. Märss, The earliest osteostracan Kalanaspis delectabilis gen. et sp. nov. from the mid-Aeronian (mid-Llandovery, lower Silurian) of Estonia. J. Vertebr. Paleontol. 38 e1425212 (2018).

72. P. S. Andreev, I. J. Sansom, Q. Li, W. Zhao, J. Wang, C.-C. Wang, L. Peng, L. Jia, T. Qiao, M. Zhu, Spiny chondrichthyan from the lower Silurian of South China. Nature 609, 969–974 (2022).

73. P. S. Andreev, I. J. Sansom, Q. Li, W. Zhao, J. Wang, C.-C. Wang, L. Peng, L. Jia, T. Qiao, M. Zhu, The oldest gnathostome teeth. Nature 609, 964–968 (2022).

74. W.-J. Zhao, M. Zhu, Siluro-Devonian vertebrate biostratigraphy and biogeography of China. Palaeoworld 19, 4–26 (2010).

75. P. Männik, V. V. Menner, R. G. Matukhin, V. Kuršs, Silurian and Devonian strata on the Severnaya Zemlya and Sedov archipelagos (Russia). Geodiversitas 24, 99–122 (2002).

76. R. E. Plotnick, Habitat of Llandoverian-Lochkovian eurypterids. World and Regional Geology, 106–131 (1999).

77. J. M. Hurst, Wenlock carbonate, level bottom, brachiopod-dominated communities from Wales and the Welsh Borderland. Palaeogeogr. Palaeoclimatol. Palaeoecol. 17, 227–255 (1975).

78. V. Karatajūtė-Talimaa, “Vertebrates” in In Silur I Devon Yugo-Vostoka Zapadno-Sibirskoj Plity, N. P. Kul′kov, V. N. Tubatolov, Eds. (1990), pp. 1–15.

79. S. Turner, New Llandovery to early Pridoli microvertebrates including Early Silurian zone fossil, Loganellia avonia nov. sp., from Britain. Final Report. Courier Forschungsinstitut Senckenberg 223, 91–127 (2000).

80. T. Märss, P. Männik, Revision of Silurian vertebrate biozones and their correlation with the conodont succession. Estonian J. Earth Sci. 62, 181 (2013).

81. X. Shan, W. Zhao, Z. Gai, A new species of Jiangxialepis (Galeaspida) from the lower Telychian (Silurian) of Jiangxi and its biostratigraphic significance. Acta Geol. Sin. - Engl. Ed. 97, 393–403 (2023).

82. W. Liu, X. Shan, X. Lin, Y. Shen, Y. Liu, Z. Zhang, Z. Gai, The first Eugaleaspiforme fish from the Silurian of the Tarim Basin reveals a close relationship between the Tarim and South China blocks at 438 mya. Palaeogeogr. Palaeoclimatol. Palaeoecol. 628, 111774 (2023).

83. Y.-L. Liu, J.-Y. Yin, R.-Y. Fan, R.-W. Zong, Y.-M. Gong, New data on Silurian (Llandovery) sinacanthids from Wuhan, South China and their biostratigraphic and paleobiogeographic implications. Palaeoworld 33, 1242–1255 (2024).

84. S. Turner, Thelodus macintoshi Stetson 1928, the largest known thelodont (Agnatha: Thelodonti). Breviora 486, 1–18 (1986).

85. N. Wang, S. Zhang, J. Wang, M. Zhu, Early Silurian chondrichthyan microfossils from Bachu County, Xinjiang, China. Vertebrata PalAsiatica 36, 257–267 (1998).

86. J. Q. Wang, N. Z. Wang, G. R. Zhang, S. T. Wang, M. Zhu, Agnathans from Llandovery (Silurian) of Kalpin, Xinjiang, China. Vertebrata PalAsiatica 40, 245–256 (2002).

87. L. Liwu, P. Jiang, Z. Lijun, New findings of middle Paleozoic agnatha and fishes from Kalpin, Xinjiang. Acta Geosci. Sin 2, 143–147 (2007).

88. Z. Jiang, Y. Wang, C. Wei, Hemipelagic deposition of the Silurian Kepingtage formation in Tarim basin and its sedimentologic significance. J. Earth Sci. 20, 921–931 (2009).

89. Y.-H. Liu, M. Zhu, X.-H. Lin, L.-W. Lu, Z.-K. Gai, A reappraisal of the Silurian galeaspids (stem-Gnathostomata) from Tarim Basin, Xinjiang. Vert. PalAsiat 57, 253–273 (2019).

90. P. S. Andreev, W. Zhao, N.-Z. Wang, M. M. Smith, Q. Li, X. Cui, M. Zhu, I. J. Sansom, Early Silurian chondrichthyans from the Tarim Basin (Xinjiang, China). PLoS One 15, e0228589 (2020).

91. X. Li, Y. Zhang, X. Lin, M. Zhu, W. Zhao, L. Tang, X. Shan, Z. Gai, New findings of Changxingaspis (Xiushuiaspidae, Galeaspida) from the Silurian of Tarim Basin and Zhejiang Province, China. Acta Geol. Sin. - Engl. Ed. 98, 531–540 (2024).

92. T. Märss, P. Mannik, T. Martma, D. Goujet, “Distribution of Silurian vertebrate micro remains in Baillie-Hamilton and Cornwallis Island sections,” in Circum-Arctic Palaeozoic Vertebrates: Biological and Geological Significance (1997; http://dx.doi.org/).

93. Q. Li, Y.-A. Zhu, J. Lu, Y. Chen, J. Wang, L. Peng, G. Wei, M. Zhu, A new Silurian fish close to the common ancestor of modern gnathostomes. Curr. Biol. 31, 3613–3620.e2 (2021).

94. J. Rong, Y. Wang, R. Zhan, J. Fan, B. Huang, P. Tang, Y. Li, X. Zhang, R. Wu, G. Wang, X. Wei, Silurian integrative stratigraphy and timescale of China. Sci. China Earth Sci. 62, 89–111 (2019).

95. W. Shi-Tao, D. Zhi-Zhong, S. Turner, Discovery of Middle Devonian Turiniidae (Thelodonti:Agnatha) from western Yunnan, China. Alcheringa Australas. J. Palaeontol. 10, 315–325 (1986).

96. M. Zhu, Z. Gai, Phylogenetic relationships of galeaspids (Agnatha). Front. Biol. China 2, 151–169 (2007).

97. J. Zalasiewicz, HOLLAND, C. H. & BASSETT, M. G. 2002. Telychian Rocks of the British Isles and China (Silurian, Llandovery Series). Geological Series no. 21. 210 pp. Cardiff: National Museums & Galleries of Wales. Price £14.99 (paperback). ISBN 0 7200 0455 1. Geol. Mag. 141, 105–106 (2004).

98. Y. Chen, Z. Gai, Q. Li, J. Wang, L. Peng, G. Wei, M. Zhu, A new family of galeaspids (jawless stem‐gnathostomata) from the llandovery, Silurian of Chongqing, southwestern China. Acta Geol. Sin. - Engl. Ed. 96, 430–439 (2022).

99. C. Yang, L. I. Qiang, Z. Zheng-Da, S. Xian-Ren, Z. H. U. You-An, W. Qian, W. E. I. Guang-Biao, Z. H. U. Min, A new genus of galeaspids (jawless stem-Gnathostomata) from the early Silurian Chongqing Lagerstätte, China. Vertebrata Palasiatica 62(4), 245–261 (2024).

100. J. Pan, Notes on Silurian vertebrates of China. Bulletin of the Chinese Academy of Geological Sciences 15, 161–190 (1986).

101. J. Pan, L. Z. Chen, Geraspididae, a new family of Polybranchiaspi- dida (Agnatha) from Silurian of northern Anhui. Vertebrata PalAsiatica 31, 225–230 (1993).

102. M. Calner, L. Jeppsson, A. Munnecke, The Silurian of Gotland - Part I: Review of the stratigraphic framework, event stratigraphy, and stable carbon and oxygen isotope development. 5, 113–131 (2004).

103. O. Bremer, Silurian vertebrates of Gotland (Sweden) and the Baltic Basin. Digital Comprehensive Summaries of Uppsala Dissertations from the Faculty of Science and Technology, ISSN 1651-6214 ; 1544 (2017).

104. H. Blom, Vertebrate remains from Upper Silurian – Lower Devonian beds of Hall Land, North Greenland. ggub 182, 1–80 (1999).

105. Ž. Žigaitė, D. Goujet, New observations on the squamation patterns of articulated specimens of Loganellia scotica (Traquair, 1898) (Vertebrata: Thelodonti) from the Lower Silurian of Scotland. geod 34, 253–270 (2012).

106. R. K. Pickerill, T. L. Harland, Trace fossils from Silurian slope deposits, North Greenland. rapggu 137, 119–133 (1988).

107. T. Märss, A. Ritchie, Articulated thelodonts (agnatha) of Scotland. Trans. R. Soc. Edinb. Earth Sci. 88, 143–195 (1997).

108. R. H. Traquair, H. Ramsay, “XXXII.—Report on Fossil Fishes Collected by the Geological Survey of Scotland in the Silurian Rocks of the South of Scotland.” Transactions of the Royal Society of Edinburgh 39, no. 3 (1900): 827–64. https://doi.org/10.1017/S0080456800035237.

109. L. D. Chen, X. H. Chen, Z. H. Li, "Silurian." Protection of Precise Geological Remains in the Yangtze Gorges Area, China with the Study of the Archean-Mesozoic Multiple Stratigraphic Subdivision and Sea-Level Change (in Chinese). Beijing: Geological Publishing House (2002): 101-130.

110. P. Jiang, D. L. Dineley, A review of early (Silurian and Devonian) vertebrate biogeography and biostratigraphy of China. Proc. R. Soc. Lond. B Biol. Sci. 235, 29–61 (1988).

111. P. Tang, J. Wang, C.-Y. Wang, R.-C. Wu, K. Yan, Y. Liang, X. Wang, Microfossils across the Llandovery–Wenlock boundary in Ziyang–Langao region, Shaanxi, NW China. Palaeoworld 24, 221–230 (2015).

112. A. Ritchie, Ainiktozoon loganense Scourfield, a proto- chordate from the Silurian of Scotland. Alcheringa 9, 117–142 (1985).

113. H. Blom, T. Märss, C. G. Miller, Silurian and earliest Devonian birkeniid anaspids from the Northern Hemisphere. Trans. R. Soc. Edinb. Earth Sci. 92, 263–323 (2002).

114. X. Shan, M. Zhu, W. Zhao, Z. Pan, P. Wang, Z. Gai, A new genus of sinogaleaspids (Galeaspida, stem-Gnathostomata) from the Silurian Period in Jiangxi, China. PeerJ 8, e9008 (2020).

115. X. R. Shan, W. J. Zhao, X. H. Lin, Y. Chen, Q. Li, M. Zhu, Z. K. Gai. The correlations of the lower red beds of early Telychian (Llandovery, Silurian) in China from the palaeoichthyological evidence. Journal of Stratigraphy,46(2): 138-153 (2002). DOI: 10.19839/j.cnki.dcxzz.2022.0011

116. Z. Gai, L. Lu, W. Zhao, M. Zhu, New polybranchiaspiform fishes (Agnatha: Galeaspida) from the Middle Palaeozoic of China and their ecomorphological implications. PLoS One 13, e0202217 (2018).

117. A. R. Carroll, S. Graham, E. Z. Chang, C. McKnight, Sinian through Permian tectonostratigraphic evolution of the northwestern Tarim basin, China. Memoir of the Geological Society of America 194 (2001).

118. W. Zhao, S. Wang, J. Wang, M. Zhu, The subdivision and correlation of the Silurian fish-bearing strata and Caledonian movement in Kalpin and Bachu regions, the Tarim Basin, Xinjiang. Journal of Stratigraphy, 33(3): 225–240 (in Chinese with English abstract) (2009).

119. M. Zhu, J.-Q. Wang, Silurian vertebrate assemblages of China. Cour. Forschungsinst. Senckenb. 223, 161–168 (2000).

120. Y. Wang, A. J. Boucot, J. Y. Rong, Community paleoecology as a geologic tool-the Chinese Ashgillian-Eifelian (latest Ordovician through early Middle Devonian) as an example. Spec Pap Geol Soc Amer 211, 1–100 (1987).

121. R.-W. Zong, Q. Liu, Y.-M. Gong, Fossil association and sedimentary environments of the Lower Silurian Fentou Formation in Wuhan, Hubei Province. J. Palaeogeog. 13, 299–308 (2011).

122. M. En-zhi, A. J. Boucot, C. Xu, R. Jia-yu, A. J. Boucot, W. B. N. Berry, “Correlation of the Silurian rocks of China” in Geological Society of America Special Papers (Geological Society of America, 1986)Geological Society of America Special Papers, pp. 1–81.

123. J. Pan, S. T. Wang, Xiushuiaspidae, a new family of Polybranchiaspi- formes from Xiushui of Jiangxi Province. Acta Palaeontologica Sinica 22, 505–509 (1983).

124. M. G. Bassett, R. Jiayu, “Brachiopods. 124-135” in Telychian Rocks of the British Isles and China, C. H. Holland, M. G. Bassett, Eds. (Silurian, Llandovery Series, 2002)vol. 21.

125. Wilson, M. V. H., Soehn, K. L., Hanke, G. F. & Märss, T. Preliminary vertebrate biostratigraphy of the Silurian Avalanche Lake sections, Mackenzie Mountains, Canada. in Circum-Arctic Palaeozoic Vertebrates: Biological and Geological Significance. (Ed. Wilson, V. H.). vol. 2 26–27 (1997).

126. K. L. Soehn, T. Märss, M. W. Caldwell, M. V. H. Wilson, New and biostratigraphically useful thelodonts from the Silurian of the Mackenzie Mountains, Northwest Territories, Canada. J. Vertebr. Paleontol. 21, 651–659 (2001).

127. I. J. Sansom, R. J. Aldridge, M. M. Smith, A microvertebrate fauna from the Llandovery of South China. Trans. R. Soc. Edinb. Earth Sci. 90, 255–272 (1999).

128. W.-J. Zhao, M. Zhu, A review of Silurian fishes from Yunnan, China and related biostratigraphy. Palaeoworld 24, 243–250 (2015).

129. K. H. S. Rozman, “Silurian Brachiopod Communities Mongolia” in Paleocommunities: Case Study from Silurian Lower Devonian, J. Boucot, J. D. Lawson, Eds. (Cambridge University Press, Cambridge, 1999), pp. 164–176.

130. Ž. Žigaitė, “A new thelodont from Lower Silurian of Tuva and north-west Mongolia” in Acta Universitatis Latviensis (2004), pp. 679:158–165.

131. P. Andreev, M. I. Coates, V. Karatajūtė-Talimaa, R. M. Shelton, P. R. Cooper, N.-Z. Wang, I. J. Sansom, The systematics of the Mongolepidida (Chondrichthyes) and the Ordovician origins of the clade. PeerJ 4, e1850 (2016).

132. R. H. Denison, Cyathaspididae. family Silurian Devonian jawless vertebrates. Fieldiana 13, 309–473 (1964).

133. T. Märss, R. Thorsteinsson, M. V. Wilson, New thelodont (Agnatha) and possible chondrichthyan (Gnathostomata) Taxa. established in the Silurian and Lower Devonian of the Canadian Arctic Archipelago. Estonian J. Earth Sci. 51, 88 (2002).

134. P. Chen, J. Jin, A. C. Lenz, Palaeoecology of transported brachiopod assemblages embedded in black shale, Cape Phillips Formation (Silurian), Arctic Canada. Palaeogeogr. Palaeoclimatol. Palaeoecol. 367-368, 104–120 (2012).

135. H. C. Stetson, Studies on the morphology of the heterostraci. J. Geol. 39, 141–154 (1931).

136. V. Karatajūtė-Talimaa, T. Märss, Upper Silurian thelodonts from Severnaya Zemlya Archipelago (Russia). Geodiversitas 24, 405–443 (2002).

137. T. Märss, Andreolepis (Actinopterygii) in the upper Silurian of northern Eurasia. Estonian J. Earth Sci. 50, 174 (2001).

138. R. Su, “New Birkeniid Anaspid from the Silurian of the Canadian Arctic,” thesis, Uppsala University, Disciplinary Domain of Science and Technology, Biology, Biology Education Centre. (2022).

139. T. Märss, Loganellia (thelodonti, agnatha) from the jaagarahu stage, Wenlock, Estonia. Estonian J. Earth Sci. 45, 189 (1996).

140. D. Palmer, J. D. Johnston, T. Dooley, K. Maguire, Short paper: The Silurian of Clew Bay, Ireland: part of the Midland Valley of Scotland? J. Geol. Soc. London 146, 385–388 (1989).

141. C. K. Maguire, J. R. Graham, Sedimentation and palaeogeographical significance of the Silurian rocks of the Louisburgh–Clare Island succession, western Ireland. Trans. R. Soc. Edinb. Earth Sci. 86, 123–136 (1995).

142. V. Karatajūtė-Talimaa, Elegestolepis grossi gen. et sp.nov., ein neuer Typ der Placoidschuppe aus dem oberen Silur der Tuwa. Palaeontographica, Abt 143, 35–50 (1973).

143. E. V. Vladimirskaya, N. P. Kulkov, Silurian brachiopod communities Tuva Paleocommunities Case Study from Silurian Lower Devonian. Press 11, 545–548 (1999).

144. D. Siveter, S. Turner, A new Silurian microvertebrate assemblage from the Tortworth inlier, Avon, England. Alcheringa Australas. J. Palaeontol. 6, 35–41 (1982).

145. T. Märss, M. V. H. Wilson, R. Thorsteinsson, Silurian and Lower Devonian thelodonts and putative chondrichthyans from the Canadian Arctic Archipelago. Spec. Pap. Palaeontol. 75, 1–144 (2006).

146. A. R. M. Blieck, V. N. Karatajūtē-Talimaa, E. Mark-Kurik, Upper Silurian and Devonian heterostracan pteraspidomorphs (Vertebrata) from Severnaya Zemlya (Russia): a preliminary report with biogeographical and biostratigraphical implications. Geodiversitas 24, 805–820 (2002).

147. Mark V. H. Wilson, M. W. Caldwell, The Furcacaudiformes: A New Order of Jawless Vertebrates with Thelodont Scales, Based on Articulated Silurian and Devonian Fossils from Northern Canada. J. Vert. Paleontol. 18, 10–29 (1998).

148. M. V. H. Wilson, K. L. Soehn, Discovery of complete Silurian fish. Sci. Nat. 77, 328–330 (1990).

149. Ž. Žigaitė, V. Karatajūtė-Talimaa, A. Blieck, Vertebrate microremains from the Lower Silurian of Siberia and Central Asia: palaeobiodiversity and palaeobiogeography. J. Micropalaeontol. 30, 97–106 (2011).

150. L. R. M. Cocks, W. S. McKerrow, J. Verniers, The Silurian of Avalonia. New York State Museum Bulletin 493, 35–53 (2003).

151. S. Turner, J. S. Peel, Silurian thelodont scales from North Greenland. rapggu 132, 79–88 (1986).

152. T. Märss, The upper Silurian cyathaspid archegonaspis from the east Baltic. Eesti NSV Teaduste Akadeemia Toimetised. Keemia. Geoloogia 26, 129 (1977).

153. E. Mark-Kurik, Distribution of vertebrates in the Silurian of Estonia. Lethaia 2, 145–152 (1969).

154. T. Märss, Paralogania from the rootsiküla (Wenlock) and paadla (Ludlow) stages of Estonia. Estonian J. Earth Sci. 52, 98 (2003).

155. P. Musteikis, J. J. Paskevicius, “Brachiopod communities of the Lithuanian Silurian” in Paleocommunities - a Case Study from the Silurian and Lower Devonian, A. J. Boucot, J. D. Lawson, Ed. (Cambridge Univ. Press, 1999), pp. 305–326.

156. T. Märss, Squamation of the thelodont agnathanPhlebolepis. J. Vertebr. Paleontol. 6, 1–11 (1986).

157. D. D. J. Antia, Temeside Bone-Bed associated sediments from Wales Welsh Borderlands. Wales Welsh Borderlands. Mercian Geologist 8, 163–215 (1981).

158. J. M. J. Vergoossen, Fish microfossils from Ramsåsa, site E, Scania, southern Sweden (mid Palaeozoic). Scripta Geologica 127, 1–70 (2004).

159. T. Märss, O. Afanassieva, H. Blom, Biodiversity of the Silurian osteostracans of the East Baltic. Earth Environ. Sci. Trans. R. Soc. Edinb. 105, 73–148 (2014).

160. D. Fredholm, Agnathan vertebrates in the lower Silurian of Gotland, Sweden. Geol. Foereningan Stockh. Foerhandlingar 112, 61–80 (1990).

161. A. Martinsson, Beyrichiacean Ostracodes Associated Earliest Silurian Vertebrates from Gotland, Stockholm. Geol. Foren. Stockholm Forh. 88. (1966).

162. T. Kiipli, R. Einasto, T. Kallaste, V. Nestor, H. Perens, S. Siir, Geochemistry and correlation of volcanic ash beds from the Rootsiküla Stage (Wenlock–Ludlow) in the eastern Baltic. Est. J. Earth Sci. 60, 207 (2011).

163. P. Janvier, H. Lelievre, A new tremataspid osteostracan Aestiaspis viitaensis n.g., n.sp., from the Silurian od Saaremaa, Estonia. Estonian J. Earth Sci. 43, 122 (1994).

164. Q. Qu, H. Blom, S. Sanchez, P. Ahlberg, Three-dimensional virtual histology of Silurian osteostracan scales revealed by synchrotron radiation microtomography. J. Morphol. 276, 873–888 (2015).

165. R. H. Traquair, IX.—Notes on Paleozoic fishes.—no. II. Ann. Mag. Nat. Hist. 2, 67–70 (1898).

166. E. K. Walton, G. J. H. Oliver, Lower Palaeozoic stratigraphy. In G. Y. Craig, Ed., Geology of Scotland, 3rd ed. (Scottish Academic Press, Edinburgh, 1991), pp. 161–193.

167. C. H. Wellman, J. B. Richardson, Terrestrial plant micro- fossils from Silurian Inliers Midland Valley Scotland. Palaeontology 36, 155–193 (1993).

168. D. Plax, T. Märss, Thelodonts (Agnatha) from the Lower Silurian (Wenlock) deposits of the northwest of Belarus. Lithosphere (Belarus) 34, 69–81 (2018).

169. N. Wang, J. Wang, G. Zhang, S. Wang, The first discovery of Silurian and early Devonian acanthodians from zoige and tewo counties, west Qinling mountains. Vertebrata Palasiatica 36, 268–281 (1998).

170. Z. Chen, Q. Chen, G. Wang, X. Fang, P. Tang, G. Yan, W. Yuan, B. Huang, X. Zhang, K. Yan, Y. Zhang, Y. Wang, Silurian integrative stratigraphy, biotas, and paleogeographical evolution of the Qinghai-Tibetan Plateau and its surrounding areas. Sci. China Earth Sci. 67, 1005–1035 (2024).

171. Z. Wen-Jin, W. Nian-Zhong, Z. H. U. Min, J. I. A. Lian-Tao, The microvertebrate remains and assemblage sequences across the Silurian/Devonian transition in West Qinling, China. Vertebrata Palasiatica 50, 309 (2012).

172. N. Davies, P. Turner, I. Sansom, A revised stratigraphy for the Ringerike Group, Norwegian Journal of Geology, Vol. 85, pp. 193-201. Trondheim (2005). ISSN 029-196X.

173. V. Hairapetian, H. Blom, C. Miller, Silurian thelodonts from the niur formation, central Iran. Acta Palaeontol. Pol. 53, 85–95 (2008).

174. D. S. Broad, A. Lenz, A new upper Silurian species of Vernonaspis (Heterostraci) from Yukon Territory, Canada. Journal of Paleontology 46, 415–420 (1972).

175. J. Kiaer, “The Downtonian and Devonian vertebrates of Spitsbergen. IV, Suborder Cyathaspida” (1932); http://hdl.handle.net/11250/173597.

176. R. H. Flower, R. Wayland-Smith, Cyathaspid fishes from the Vernon shale of New York. Bulletin of the Museum of Comparative Zoology 107, 353–387 (1952).

177. W. L. Bryant, On the Structure of Plaeaspis and on the Occurrence in the United States of Fossil Fishes Belonging to the Family Pteraspidae. Proc. Amer. Phil. Sk 65, 256–271 (1926).

178. D. L. Fillmore, E. L. Simpson, S. G. Lucas, M. J. Szajna, S. Ireland, A. J. Bouknight, Continental ichnofossil assemblage from the upper Silurian of Laurentia: The Bloomsburg Formation of eastern Pennsylvania. Palaeogeogr. Palaeoclimatol. Palaeoecol. 547, 109693 (2020).

179. B. Jones, O. A. Dixon, Stratigraphy and sedimentology of Upper Silurian rocks, northern Somerset Island, Arctic Canada. Can. J. Earth Sci. 14, 1427–1452 (1977).

180. D. K. Elliott, D. L. Dineley, New Species of Protopteraspis (Agnatha, Heterostraci) from the (?) Upper Silurian to Lower Devonian of Northwest Territories, Canada. J. Paleontol. 57, 474–494 (1983).

182. D. K. Elliott, A new subfamily of the Pteraspididae (Agnatha, Heterostraci) from the Upper Silurian and Lower Devonian of Arctic Canada. Palaeontology 27, 169–197 (1984).

181. D. S. Broad, D. L. Dineley, New Upper Silurian Lower Devonian Genus Cyathaspididae (Ostracodermi) from Arctic Canada. Geological Survey of Canada Bulletin 222: 53–90 (1973).

182. D. Dineley, S. Metcalf, Fossil Fishes of Great Britain. Geological Conservation Review Series 16 (JNCC, Peterborough, 1999).

183. T. Klaos, T. Märss, H. Perens, Sedimentation of the Himmiste-Kuigu fish bed (Ludlow of Estonia) and taphonomy of the Phlebolepis elegans Pander (Thelodonti) shoal. Estonian J. Earth Sci. 52, 239 (2003).

184. S. Turner, C. J. Burrow, Microvertebrates from the Silurian–Devonian boundary beds of the Eastport Formation, Maine, eastern USA. Atl. Geol. 54, 171–187 (2018).

185. D. Elliott, New pteraspididae (agnatha, heterostraci) from the lower Devonian of Northwest Territories, Canada. J. Vert. Paleontol. 2, 389–406 (1983).

186. G. M. Robertson, New genera of ostracoderms from the Upper Silurian of Oesel. Journal of Paleontology 12, 486–493 (1938).

187. D. L. Dineley, The “red stratum” of the Silurian arisaig series, Nova Scotia, Canada. J. Geol. 71, 523–524 (1963).

188. W. Gross, Fragliche actinopterygier‐schuppen aus Dem silur gotlands. Lethaia 1, 184–218 (1968).

189. O. Bremer, H. Blom, An updated stratigraphic and environmental framework for the distribution of Silurian vertebrates on Gotland. Estonian J. Earth Sci. 64, 13 (2015).

190. M. E. Eriksson, E. K. Nilsson, L. Jeppsson, Vertebrate extinctions and reorganizations during the Late Silurian Lau Event. Geology 37, 739–742 (2009).

191. L. Cherns, Silurian chitons as indicators of rocky shores and lowstand on Gotland, Sweden. Palaios 14, 172 (1999).

192. O. Bremer, E. Jarochowska, T. Märss, Vertebrate remains and conodonts in the upper Silurian Hamra and Sundre formations of Gotland, Sweden. GFF 142, 52–80 (2020).

193. A. Kunth, Ueber Pteraspis (Geologischen Gesellschaft, Zeitschrift der Deutschen, 1872)vol. 24.

194. E. B. Giffin, Silurian vertebrates from Pennsylvania. Journal Paleontology 53, 438–445 (1979).

195. G. M. Robertson, The Ostracoderm order Anaspida, with descrip- tion of some Upper Silurian material. Trans. Kans. Acad. Sc 44, 314–317 (1941).

196. G. M. Robertson, The status of cephalaspis schrenckii pander from the upper Silurian of oesel. J. Geol. 47, 649–657 (1939).

197. G. M. Robertson, Cephalaspids from Upper Silurian Oesel, discussion cephalaspid genera: AM. Jour. Sci 243, 169–191 (1945).

198. A. Ritchie, The late Silurian anaspid genus Rhyncholepis from Oesel, Estonia, and Ringerike, Norway. American Museum novitates; no. 2699. American Museum of Natural History 2699 (1980).

199. G. M. Robertson, Some Paleoecoloical Speculations Regarding the Earliest Vertebrates. Proceedings of the Iowa Academy of Science 57, 491–497 (1950).

200. W. J. Zhao, M. Zhu, Z. K. Gai, Z. H. Pan, X. D. Cui, J. C. Cai, A review Silurian fishes from north-western Hunan, China related biostratigraphy. Acta Geologica Polonica 68, 475–1468 (2018).

201. J. Rong, Y. Wang, X. Zhang, Tracking shallow marine red beds through geological time as exemplified by the lower Telychian (Silurian) in the Upper Yangtze Region, South China. Sci. China Earth Sci. 55, 699–713 (2012).

202. S. Turner, J. M. J. Vergoossen, G. C. Young, Fish microfossils from Irian Jaya. Memoirs Association Australasian Palae- ontologists 18, 165–178 (1995).

203. W. A. Oliver Jr, A. E. H. Pedder, R. J. Weiland, A. Q. van Ufford, Middle Palaeozoic corals from the southern slope of the Central Ranges of Irian Jaya, Indonesia. Alcheringa Australas. J. Palaeontol. 19, 1–15 (1995).

204. C. J. Burrow, S. Turner, G. C. Young, Middle Palaeozoic microvertebrate assemblages and biogeography of East Gondwana (Australasia, Antarctica). Palaeoworld 19, 37–54 (2010).

205. L.-Z. Shi, Z.-Z. Wang, Silurian intermediate–felsic complex in the Xiangtaohu area of central Qiangtang, northern Tibet: Evidence for southward subduction of the Longmuco–Shuanghu Prototethys oceanic plate: Comment. Lithos 410-411, 106559 (2022).

206. J. A. Talent, M. R., S. A., “Silurian of Australia and New Guinea: Biostratigraphic Correlationsand Paleogeography” in Silurian Lands and Seas, L. E., M. E. Johnson, Eds. (University of State of New York, USA, 2003)New York State Museum Bulletin, pp. 181–220.

207. C. N. Bowman, S. A. Young, D. Kaljo, M. E. Eriksson, T. R. Them II, O. Hints, T. Martma, J. D. Owens, Linking the progressive expansion of reducing conditions to a stepwise mass extinction event in the late Silurian oceans. Geology 47, 968–972 (2019).

208. N.-Z. Wang, Two new Silurian galeaspids (jawless craniates) from Zhejiang Province, China, with a discussion of galeaspid-gnathostome relationships. In M.-M. Chang, Y.-H. Liu, G.-R. Zhang, Eds., Early vertebrates and related problems of evolutionary biology (Science Press, Beijing, 1991), pp. 41–66.

209. W. J. Zhao, M. Zhu, S. Liu, Z. H. Pan, L. T. Jia, A new look Silurian fish-bearing strata around Shanmen reservoir Lixian, Hunan Province. *Journal Stratigraphy* 40, 349–358 (2016).

210. D. D. J. Antia, Shell laminae and shell orientation in the upper Silurian, Overton Formation, U.K. Palaeogeogr. *Palaeoclimatol. Palaeoecol.* 32, 119–133 (1980).

211. J.-C. Cai, W.-J. Zhao, M. Zhu, Subdivision and age of the Silurian fish-bearing Kuanti Formation in Qujing, Yunnan Province. *Vert. PalAsiat* 58, 249–266 (2020).

212. N.-Z. Wang, Z.-Z. Dong, Discovery of Late Silurian microfossils of Agnatha and fishes from Yunnan, China. *PalAsiat* 28, 201–206 (1989).

213. W. Zhao, The trip on tracing the Silurian fishes. China Nature (5), 66–70 (2010).

214. M. Zhu, W. Zhao, L. Jia, J. Lu, T. Qiao, Q. Qu, The oldest articulated osteichthyan reveals mosaic gnathostome characters. Nature 458, 469–474 (2009).

215. B. Choo, M. Zhu, Q. Qu, X. Yu, L. Jia, W. Zhao, A new osteichthyan from the late Silurian of Yunnan, China. PLoS One 12, e0170929 (2017).

216. M. Zhu, X. Yu, P. E. Ahlberg, B. Choo, J. Lu, T. Qiao, Q. Qu, W. Zhao, L. Jia, H. Blom, Y. ’an Zhu, A Silurian placoderm with osteichthyan-like marginal jaw bones. Nature 502, 188–193 (2013).

217. M. Zhu, P. E. Ahlberg, Z. Pan, Y. Zhu, T. Qiao, W. Zhao, L. Jia, J. Lu, A Silurian maxillate placoderm illuminates jaw evolution. Science 354, 334–336 (2016).

218. Y.-A. Zhu, J. Lu, M. Zhu, Reappraisal of the Silurian placoderm Silurolepis and insights into the dermal neck joint evolution. R. Soc. Open Sci. 6, 191181 (2019).

219. C. Miller, T. Märss, H. Blom, New anaspid material from the Late Silurian of Britain and Estonia. Acta Universitatis Latviensis 679, 46–56 (2004).

220. R. Einasto, “Locality 7:3 Kaugatuma cliff” in Field Meeting Estonia 1990. An Excursion Guidebook, D. Kaljo, H. Nestor, Eds. (Estonian Academy of Sciences, 1990), pp. 174–175.

221. O. Bremer, G. Niedźwiedzki, B. H., M. Dec, W. Kozłowski, The First Vertebrate Microremains from the Upper Silurian of the Holy Cross Mountains, Poland (2017).

222. K. Mehlqvist, K. Larsson, V. Vajda, Linking upper Silurian terrestrial and marine successions—Palynological study from Skåne, Sweden. Rev. Palaeobot. Palynol. 202, 1–14 (2014).

223. C. J. Burrow, S. Turner, G. S. Nowlan, R. H. Denison, Vertebrate microremains from the late Silurian of Arisaig, Nova Scotia, Canada. J. Paleontol. 87, 1041–1059 (2013).

224. C. J. Burrow, D. Rudkin, Oldest near-complete acanthodian: the first vertebrate from the Silurian Bertie Formation Konservat-Lagerstätte, Ontario. PLoS One 9, e104171 (2014).

225. T. Märss, V. Karatajūtė-Talimaa, Late Silurian–Early Devonian tessellated heterostracanOniscolepisPander, 1856 from the East Baltic and North Timan. Estonian J. Earth Sci. 58, 43 (2009).

226. N. S. Davies, I. J. Sansom, P. Turner, Trace fossils and paleoenvironments of a late Silurian marginal-marine/alluvial system: The ringerike group (lower old red sandstone), Oslo region, Norway. Palaios 21, 46–62 (2006).

227. E. J. Loeffler, B. Jones, Additional late Silurian ostracoderms from the Leopold Formation of Somerset Island, North West Territories, Canada. Palaeontology 20_part34, 661–667 (1977).

228. A. D. Miall, J. W. Kerr, Phanerozoic stratigraphy and sedimentology of Somerset Island and northeastern Boothia Peninsula. Geological Survey of Canada Paper (1977).

229. G. F. Matthew, “On Some Remarkable Organisms of the Silurian and Devonian Rocks in Southern New Brunswick” in Ott. Nat., A. H. (Henry Marc), Ed. (Ottawa, Ottawa Field-Naturalists’ Club, 1889)vol. 3, pp. 74–77.

230. G. F. Matthew, A new genus and a new species of Silurian fish. Transactions of the Royal Society of Canada 3rd series, 7–11 (1907).

231. R. H. Denison, S. K. Roy, L. A. Ross, A Review of the Habitat of the Earliest Vertebrates (Chicago Natural History Museum Press, 1956)vol. 11:no.8 (1956).

232. C. J. Burrow, A partial articulated acanthodian from the Silurian of New Brunswick, Canada. Can. J. Earth Sci. 48, 1329–1341 (2011).

233. D. K. Elliott, S. Swift, A new species ofAriaspis(Agnatha, Heterostraci) from the Late Silurian of the Canadian Arctic. J. Vertebr. Paleontol. 30, 1874–1878 (2010).

234. E. J. Loeffler, D. L. Dineley, ncw specics Corvaspis from Peel Sound Formation Somerset Island. Palaeontology 19, 757–766 (1976).

235. D. Miall, J. W. Kerr, M. R. Gibling, Somerset Is- land Formation: Upper Silurian ?Lower Devonian inter- tidal/supratidal succession, Boothia Uplift region, Arctic Canada. Arctic Canada. Canadian Journal Earth Sciences 15, 81–189 (1978).

236. L. I. Novitskaya, Evolution of generic and species diversity in agnathans (Heterostraci: Orders Cyathaspidiformes, Pteraspidiformes). Paleontological Journal 41, 268–280 (2007).

237. J. Valiukevicius, New Late Silurian Middle Devonian Acanthodians Timan-Pechora Region (Acta Geologica Polonica, 2003)vol. 53. 3. pp209–245.

238. A. C. Lenz, Llandoverian Graptolites of the Northern Canadian Cordillera : Petalograptus, Cephalograptus, Rhaphidograptus, Dimorphograptus, Retiolitidae, and Monograptidae (Royal Ontario Museum, Toronto, 1982).

239. A. Blieck, P. Janvier, Silurian vertebrates. Spec. Pap. Palaeontol. 44, 345–389 (1991).
